# Natural product inspired optimization of a selective TRPV6 calcium channel inhibitor†
†Electronic supplementary information (ESI) available: Tables S1–S3, Fig. S1–S4, protocols for FLIPR assays, confocal imaging and antiproliferative assays, preparation, crystal structure reports, HRMS results, ^1^H, ^13^C and ^19^F NMR spectra of all new compounds, and purity of final compounds. CCDC 1997201–1997205. For ESI and crystallographic data in CIF or other electronic format see DOI: 10.1039/d0md00145g


**DOI:** 10.1039/d0md00145g

**Published:** 2020-07-16

**Authors:** Micael Rodrigues Cunha, Rajesh Bhardwaj, Aline Lucie Carrel, Sonja Lindinger, Christoph Romanin, Roberto Parise-Filho, Matthias A. Hediger, Jean-Louis Reymond

**Affiliations:** a Department of Chemistry and Biochemistry , University of Bern , Freiestrasse 3 , 3012 Bern , Switzerland . Email: jean-louis.reymond@dcb.unibe.ch; b Department of Pharmacy , University of São Paulo , Prof. Lineu Prestes Avenue 580 , 05508-000 São Paulo , Brazil . Email: roberto.parise@usp.br; c Department of Nephrology and Hypertension , University Hospital Bern , Inselspital , 3010 Bern , Switzerland . Email: matthias.hediger@ibmm.unibe.ch; d Institute of Biophysics , Johannes Kepler University Linz , Gruberstrasse 40 , 4020 Linz , Austria

## Abstract

Transient receptor potential vanilloid 6 (TRPV6) is a calcium channel implicated in multifactorial diseases and overexpressed in numerous cancers. We recently reported the phenyl-cyclohexyl-piperazine *cis*-**22a** as the first submicromolar TRPV6 inhibitor. This inhibitor showed a seven-fold selectivity against the closely related calcium channel TRPV5 and no activity on store-operated calcium channels (SOC), but very significant off-target effects and low microsomal stability. Here, we surveyed analogues incorporating structural features of the natural product capsaicin and identified 3OG, a new oxygenated analog with similar potency against TRPV6 (IC_50_ = 0.082 ± 0.004 μM) and ion channel selectivity, but with high microsomal stability and very low off-target effects. This natural product-inspired inhibitor does not exhibit any non-specific toxicity effects on various cell lines and is proposed as a new tool compound to test pharmacological inhibition of TRPV6 mediated calcium flux in disease models.

## Abstract 2

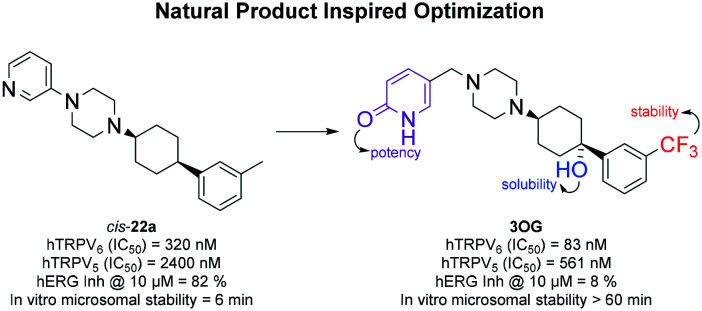
Natural product derived analogues were surveyed, and an oxygenated analog was identified as a potent and selective TRPV6 inhibitor, with high microsomal stability and low off-target effects.

## Introduction

TRPV6 is a Ca^2+^-selective member of the transient receptor potential vanilloid (TRPV) family, referred to as the gatekeeper of transepithelial Ca^2+^ transport.^1^–^3^ The channel is primarily found in the human intestine, kidney and placenta and in a number of exocrine organs such as pancreas, prostate and mammary gland.^4^,^5^ It is known that TRPV6 has an important contribution to multifactorial diseases.^6^,^7^ For instance, TRPV6-deficient mice have diminished fertility, osteopenia and reduced body weight,^5^,^8^ whilst human TRPV6 mutations, which render the channel less functional, cause transient neonatal hyperparathyroidism (TNH) and skeletal abnormalities.^9^,^10^ These pathological findings are related to tissues in which TRPV6-expression at normal levels is essential for Ca^2+^ homeostasis. On the other hand, TRPV6 expression was found to be abnormally upregulated in numerous cancers of breast^11^–^13^ and prostate tissues,^14^ compared to normal tissues.^15^,^16^

**Fig. 1:**
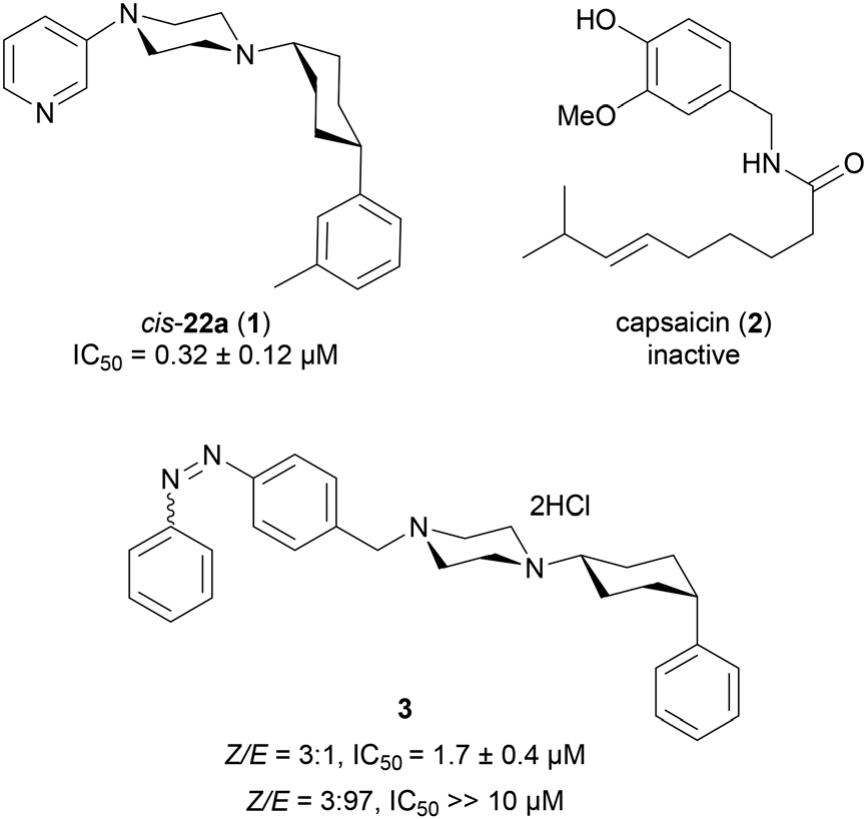
Chemical structure and *h*TRPV6 inhibition potency of *cis*-**22a** (**1**), capsaicin (**2**), and the photoswitchable inhibitor **3** (shown as observed in X-ray structures).^17^–^19^

We recently reported *cis*-**22a** (**1**, Fig. 1) as the first submicromolar small molecule inhibitor of *h*TRPV6 mediated Ca^2+^ flux.^17^ Compound **1** was highly selective for *h*TRPV6 against other Ca^2+^ channels, and showed a 7-fold selectivity against the closely related *r*TRPV5. Inhibitor **1** furthermore decreased the cell viability of a tumor cell line overexpressing TRPV6 as reported with siRNA knockdown experiments; however the effect only occurred at high concentrations (IC_50_ ≈ 25 μM). However, **1** also inhibited other targets such as *h*ERG, dopamine and muscarinic receptors, and was highly unstable against microsomal degradation, implying that this inhibitor only had limited applicability as a tool compound to study *h*TRPV6 inhibition.

Herein, we set out to search for new analogs with similar potency and selectivity but an improved pharmacological profile. To this end, we introduced the structural features of the natural product capsaicin (**2**),^20^–^22^ a well-known TRPV1 ligand also reported as a *h*TRPV6 modulator.^23^,^24^ Our aim was to modify the 3-pyridine and *m*-phenyl appendages of **1**, which are frequent drug-type substituents and might be responsible for the undesirable off-target effects of **1**. Previous structure–activity relationship studies had shown that variations in both groups were often compatible with *h*TRPV6 inhibition,^17^ such as introducing a phenyl diazo group to obtain the photoswitchable *h*TRPV6 inhibitor **3**.^18^ On the other hand, modifications of the central *cis*-1,4-cyclohexane and piperazine groups mostly abolished the activity.^17^ Therefore, we maintained this central core in all derivatives of **1** investigated in the present study. We also reinvestigated **2** itself to verify the claimed activity of this natural product on *h*TRPV6 and extended the investigation to a series of new analogues.

## Results and discussion

### 1. Design and synthesis

**Scheme 1:**
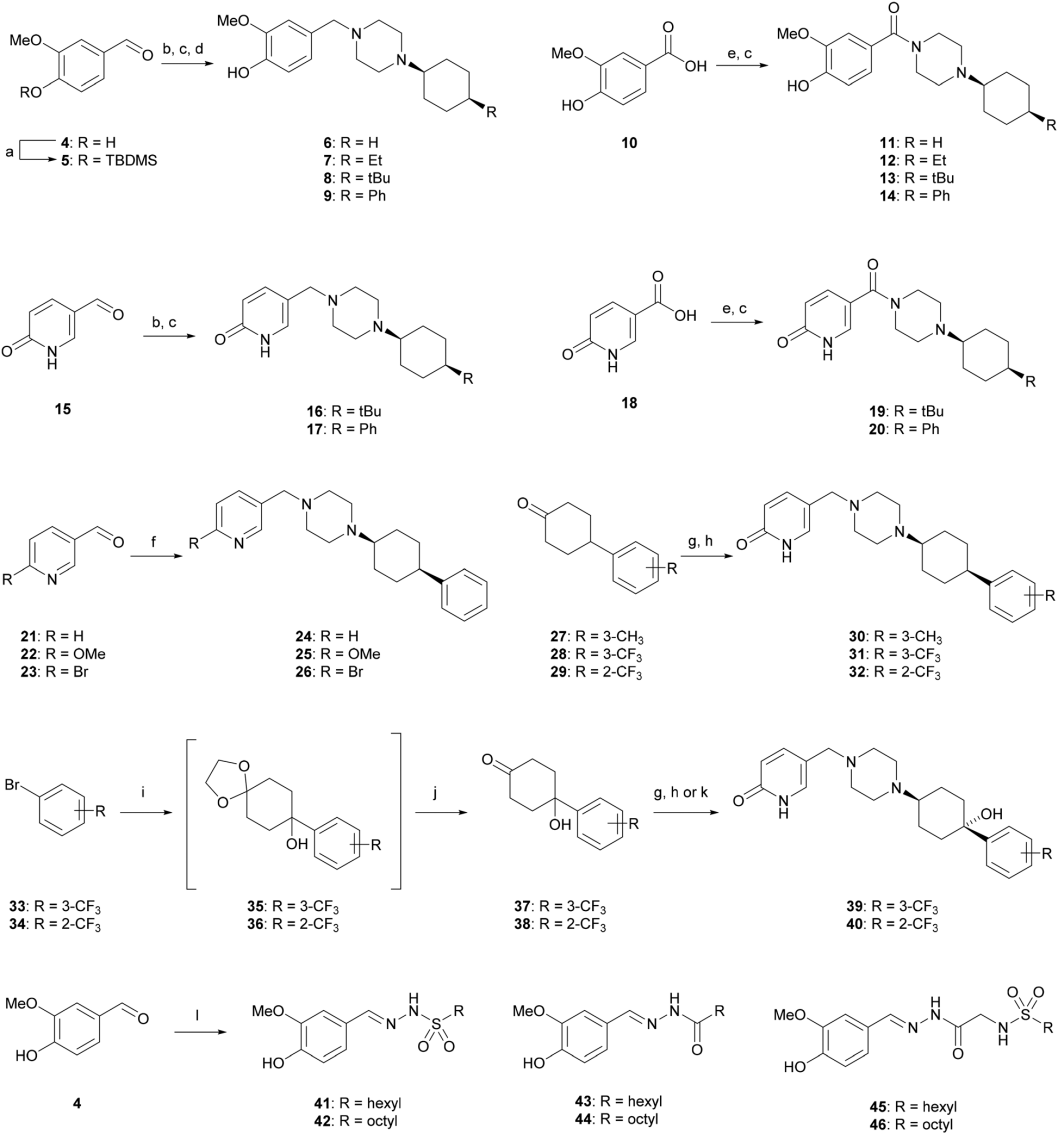
Synthesis of TRPV6 inhibitors and capsaicin analogues. Reagents and conditions. (a) TBDMSCl, DMAP, imidazole, DCM, r.t., 2 h (quant.); (b) (i) *tert*-butyl piperazine-1-carboxylate, AcOH, NaBH(OAc)_3_, DCE, r.t., 48 h; (ii) TFA, DCM, r.t., 1 h (78–45%, over 2-steps); (c) 4-*R*-cyclohexanones, NaBH(OAc)_3_, Et_3_N, DCE, r.t., 48 h (6–45%); (d) TBAF, THF, r.t., 3 h (30–59%, over 2-steps); (e) (i) *tert*-butyl piperazine-1-carboxylate, EDCl, DMAP, CH_2_Cl_2_, r.t., on; (ii) TFA, DCM, r.t., 1 h, (60%, over 2-steps); (f) 4-phenylcyclohexyl-piperazine, AcOH, NaBH(OAc)_3_, DCE, r.t., 48 h (6–68%); (g) (i) 1-benzylpiperazine, NaBH(OAc)_3_, DCE, r.t., 48 h; (ii) Pd/C, H_2_, AcOH, MeOH, r.t., on. (18–38%, over 2-steps); (h) **38**, **15**, AcOH, NaBH(OAc)_3_, DCE, r.t., 48 h (81%); (i) Mg, THF, Ar., r.t. to rf., 30 min; then 1,4-dioxaspiro[4.5]decan-8-one, THF, Ar., r.t. to rf., 30 min; (j) PPTS, acetone, H_2_O, 60 °C, 6 h (72–90%); (k) **37**, **15**, NaBH_3_CN, Et_3_N, MeOH, r.t., 24 h (48%); (l) hexyl- or octyl-sulfonyl-hydrazide, MeOH, r.t., 2 h (56–73%).

The synthesis of all compounds investigated in this study is presented in Scheme 1. First, we replaced the pyridine ring in **1** with the *O*-methyl-catechol group of capsaicin while either preserving the phenyl appendage or substituting it with aliphatic groups resembling the fatty acyl group of capsaicin. Reductve alkylation of silyl protected vanillin **5** with Boc-piperazine and Boc removal, followed by a second reductive alkylation with cyclohexanone and subsequent deprotection yielded analog **6**. The same reaction sequence with 4-ethyl-, 4-*tert*-butyl- or 4-phenyl-cyclohexanone gave the corresponding analogues **7–9**. Condensation of vanillic acid (**10**) with *N*-Boc-piperazine, Boc deprotection and reductive alkylation with the same four cyclohexanones afforded analogues **11–14** including an amide linkage related to capsaicin. In a similar approach starting with 6-hydroxynicotinaldehyde (**15**) and 6-hydroxynicotinic acid (**18**), we obtained 4-phenyl- and 4-*tert*-butyl-cyclohexyl analogues **16–17** and **19–20** displaying a pyridone group. Pyridone can be considered as a pyridine analog containing a hydrogen-bond donor group related to the phenolic hydroxyl group of capsaicin.

Due to the activity of **17** (see below), we prepared further analogues of this compound. First, we modified the pyridone carbonyl group with a methoxy (**25**) or bromo (**26**) substituent by double reductive alkylation of the corresponding pyridine-carboxaldehydes **21–23**, and similarly prepared a new sample of the previously reported *meta*-pyridine analog **24**.^17^ We also synthesized analogues **30–32** by combining the pyridone group as a piperazine substituent with a *meta*-xylyl group as a cyclohexyl substituent as in **1**, or with an *ortho*- or *meta*-trifluoromethyl phenyl group, by using a similar synthetic route starting from the corresponding cyclohexanones. For the particularly lipophilic trifluoromethyl derivatives, we additionally synthesized analogues **39** and **40** preserving the tertiary alcohol at the cyclohexane, which is present as an intermediate for cyclohexanone synthesis, such as to enhance water solubility.

Inspired by a report that **2** itself inhibits TRPV6,^23^,^24^ we finally prepared a series of capsaicin analogues reproducing the essential pharmacophoric features of the natural product (a methyl-catechol and a hydrophobic tail connected *via* an amide bond linker) by connecting vanillin with aliphatic chains *via* a sulfonylhydrazone (**41** and **42**),^25^*N*-acyl hydrazone (**43** and **44**)^26^ or sulfonylglycine hydrazone (**45** and **46**)^27^,^28^ as an amide replacement.^29^,^30^

### 2. X-ray crystallography

**Fig. 2:**
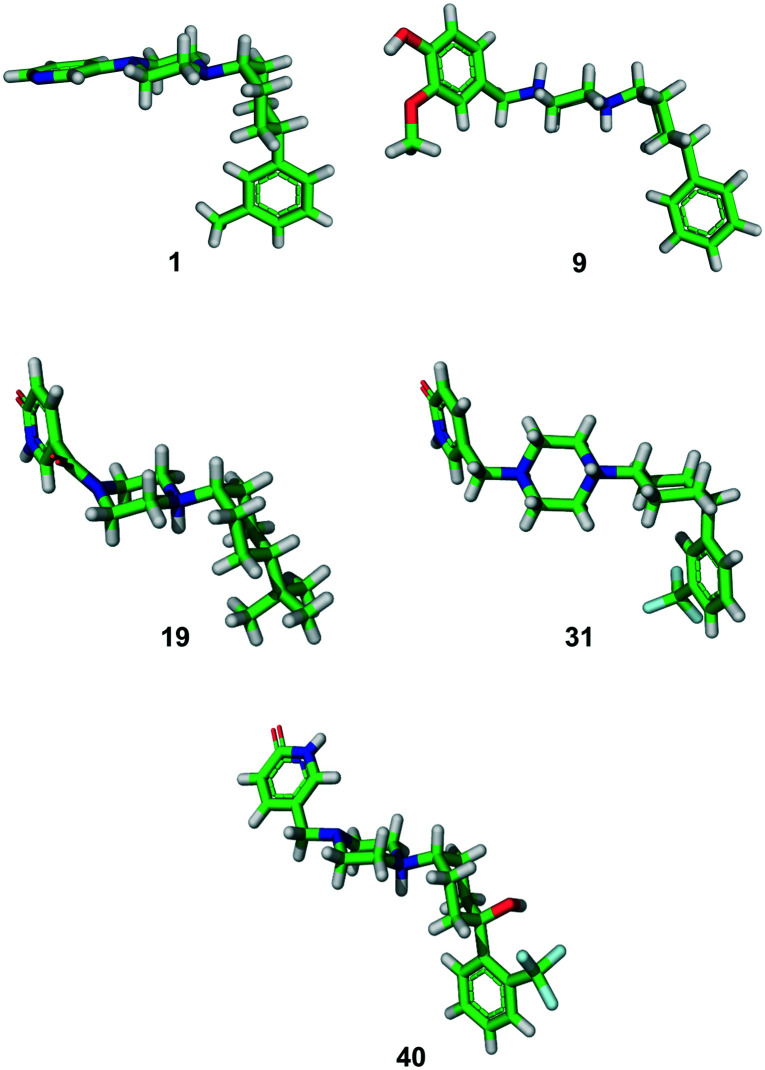
X-ray structures of compounds **1** (free base), **9** and **31** (di-HCl salts), and **19** and **40** (mono-HCl salts).

In all the above syntheses, the *cis*-1,4-cyclohexyl stereoisomer was isolated either by column chromatography or by RP-HPLC. All the compounds obtained as free bases were finally precipitated as hydrochloride salts. In the case of a freshly synthesized sample of the original inhibitor **1** and of analogues **9**, **19**, **31** and **40**, we obtained X-ray crystal structures confirming the 1,4-*cis*-cyclohexane stereochemistry (Fig. 2). In these structures, the cyclohexane and piperazine rings adopt a chair conformation. Furthermore, the piperazinyl group is an axial substituent of the cyclohexane ring for **1**, **9**, **19**, and **40**, but an equatorial substituent in **31** as observed previously with the photoswitchable inhibitor **3**,^18^ possibly reflecting crystal packing effects (Fig. S1†).

### 3. TRPV6 activity screening

**Fig. 3:**
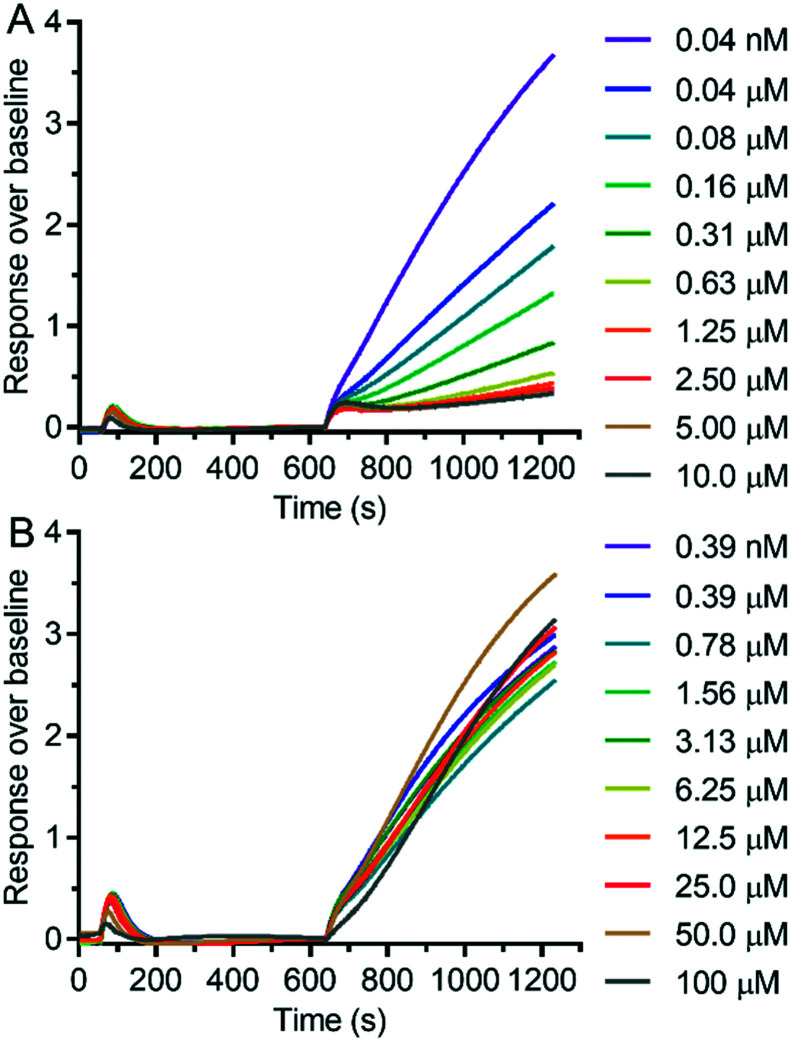
Dose–response curves of **1** (A) and **2** (B) in HEK-*h*TRPV6 cells, measuring Cd^2+^ influx.

We measured the possible modulation of TRPV6 activity by detecting the uptake of Cd^2+^ into HEK293 cells stably overexpressing human TRPV6 (HEK-*h*TRPV6) using the fluorescence reporter Calcium-5 (Molecular Devices LLC) as described previously.^17^ The HEK-*h*TRPV6 cells were previously reported in our group^31^–^33^ and are routinely used for screening campaigns. We first measured the inhibition potency of reference compounds **1** and **2** in this assay (Fig. 3). The assay confirmed the submicromolar activity of our cyclohexylpiperazine inhibitor **1**, showing an even slightly stronger inhibition than the originally reported value. On the other hand, we could not detect any modulation of *h*TRPV6 activity by **2** in this assay up to 100 μM, in contrast to the reported activity, which was determined indirectly.^23^,^24^

**Fig. 4:**
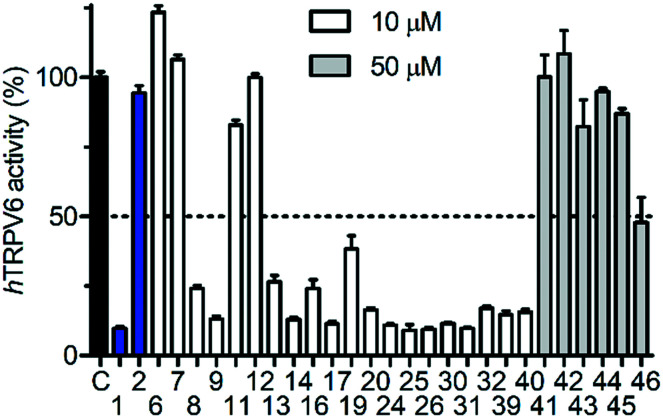
Activity screening for *h*TRPV6 mediated Cd^2+^ uptake in HEK-*h*TRPV6 cells. Data shown are mean + SEM (*n* = 3).

We then proceeded to test the initial series of analogues **6–9**, **11–14**, **19–20**, **24–26**, and **41–46** (Fig. 4 and Table 1). None of the capsaicin analogues **41–46** showed any activity, in line with the lack of *h*TRPV6 inhibition observed with **2**. Nevertheless, the *O*-methyl-catechol group borrowed from capsaicin was suitable as a replacement for the *meta*-pyridine group of **1** when combined with a *tert*-butyl (**8** and **13**) or phenyl (**9** and **14**) group as a cyclohexyl substituent, in particular with **14** featuring a vanillic amide group, illustrating that a dibasic piperazine is not essential for activity. We also observed good *h*TRPV6 inhibitory activity with three of the four analogues for which the *meta*-pyridine group was replaced by a pyridone group (**16**, **17**, and **20**). The lower activity with **19** however showed that the *tert*-butyl group, although potentially interesting as a non-aromatic group, was not a very favourable replacement of the aromatic group as a cyclohexane substituent. We selected pyridone **17** (IC_50_ = 0.37 μM) for further optimization due to its good activity combined with a favorable ligand efficiency (LE = 0.34) and lipophilic ligand efficiency (LLE = 3.5)^34^ compared to all other tested compounds.

**Table 1:** Activity on *h*TRPV6

Cpd	IC_50_ (μM) (95% CI)^*a*^	HAC^*b*^	LE^*c*^	LLE^*d*^
**1**	0.050 ± 0.003	25	0.40	1.7
**8**	2.4 (1.4–4.2)	26	0.30	0.3
**9**	1.6 (1.1–2.3)	28	0.28	0.9
**13**	2.1 (1.5–2.9)	27	0.29	1.0
**14**	0.43 (0.31–0.59)	29	0.30	2.1
**16**	2.4 (0.30–19.0)	24	0.32	2.1
**17**	0.37 (0.25–0.57)	26	0.34	3.5
**20**	0.60 (0.45–0.80)	27	0.32	3.9
**24**	0.55 (0.35–0.87)	25	0.34	2.0
**25**	0.55 (0.40–0.77)	27	0.32	1.3
**26**	0.17 (0.14–0.22)	26	0.36	1.6
**30**	0.15 ± 0.04	27	0.35	3.2
**31**	0.064 ± 0.007	30	0.33	3.2
**32**	0.69 ± 0.18	30	0.28	2.2
**39** (3OG)	0.082 ± 0.004	31	0.31	4.8
**40**	0.13 ± 0.03	31	0.30	4.6

^*a*^Data shown are mean ± SEM (*n* = 9/concentration) for at least 2 independent experiments or mean and 95% confidence interval (*n* = 6/concentration) of a single experiment.

^*b*^HAC: heavy atom count.

^*c*^LE: ligand efficiency = (1.37 × pIC_50_)/HAC, where pIC_50_ = –log(IC_50_) in the molar range.^34^

^*d*^LLE: lipophilic ligand efficiency = pIC_50_ – clog *P*, where clog *P* was obtained from ChemDraw v. 19.0.1.28 (PerkinElmer Informatics, Inc.).^34^

### 4. Optimization of pyridone **17**

**Fig. 5:**
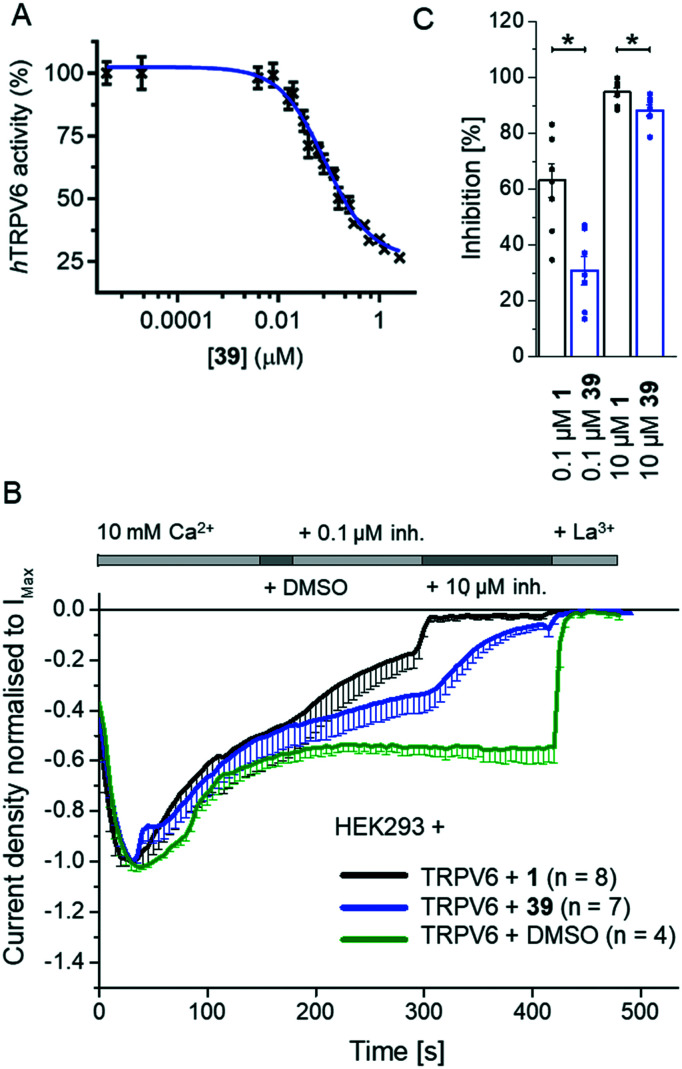
Evaluation of **39** (3OG) as a TRPV6 inhibitor. (A) Dose–response curve of **39** on Cd^2+^ influx into HEK-*h*TRPV6 cells. Data shown are mean ± SEM (*n* = 6/concentration) of 4 independent experiments. (B) and (C) Electrophysiological characterisation of **39**. (B) Averaged time course of whole-cell current densities (mean ± SEM) from YFP-TRPV6 transfected HEK293 cells. The experiment started at 10 mM Ca^2+^, subsequently containing equivalent amounts of DMSO as the control (green) or 0.1 and 10 μM **39** (blue) or **1** (black), followed by La^3+^. (C) Bar graph (mean ± SEM and individual values) of inhibition by 0.1 and 10 μM **39** (blue) and **1** (black) on TRPV6 current densities. * indicates significant *p* values < 0.05.

We investigated several changes around the phenyl group as a means to improve the activity of **17**. Inhibition increased when adding a *meta*-CH_3_ group similar to **1** to form **30** (IC_50_ = 0.15 ± 0.04 μM). The effect was even stronger with a *meta*-CF_3_ group (**31**, IC_50_ = 0.064 ± 0.007 μM), while the inhibition decreased slightly with an *ortho*-CF_3_ group (**32**, IC_50_ = 0.69 ± 0.18 μM). Introducing a hydroxyl group at the aromatic attachment point on the cyclohexane caused a slight decrease in potency for the *meta*-CF_3_ analog **39** (IC_50_ = 0.082 ± 0.004 μM, Fig. 5A), but an increase in potency for the *ortho*-CF_3_ analog **40** (IC_50_ = 0.13 ± 0.03 μM). In both cases, the addition of the hydroxyl group also increased water solubility. Analyzing all the analogues in terms of LE and LLE led us to select **39**, named 3OG, as a promising analog for further investigation.

Electrophysiological experiments on YFP-TRPV6 transiently transfected HEK293 cells confirmed the activity of **39** on TRPV6. Initially, 10 mM Ca^2+^ solution was applied and after the currents reached a plateau, the inhibitors at 0.1 and 10 μM were added. Finally, the calcium currents were fully inhibited with La^3+^ (Fig. 5B). **39** induced an inhibition of 30.8 ± 5.1% and 88.2 ± 2.0%, respectively, at 0.1 μM and 10 μM (Fig. 5C). In comparison to the previously described **1**, the inhibition by **39** occurred at a slower rate and was less pronounced.

### 5. Blocking TRPV6 transport function with pyridone **39**

**Fig. 6:**
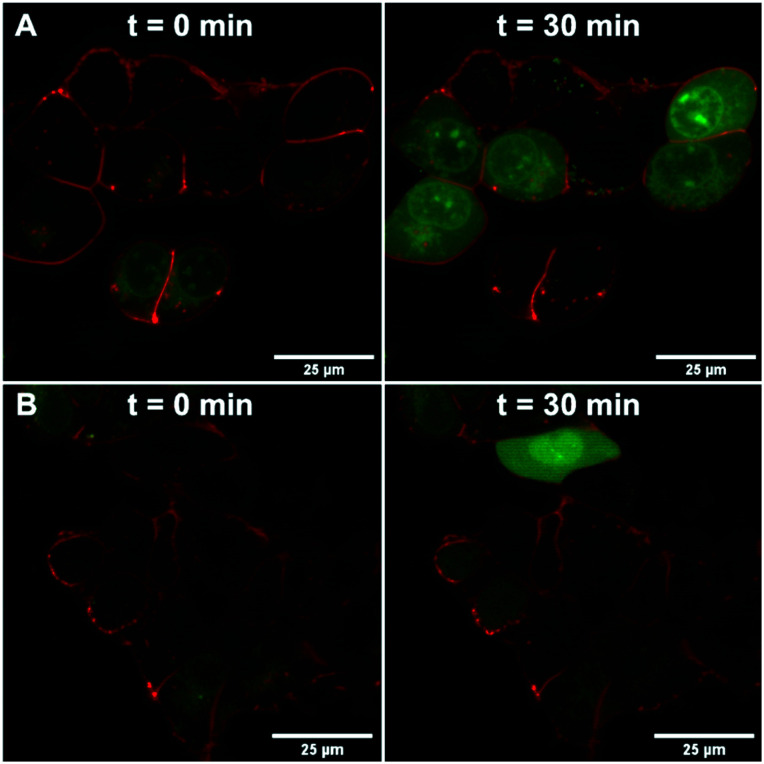
HEK-*h*TRPV6 cells co-stained with Leadmium™ Green and wheat germ agglutinin Alexa Fluor™ 594 conjugate. Images were collected at time 0 min and 30 min after treatment with the vehicle (A) or 10 μM **39** (B) followed by the addition of Cd^2+^ (50 μM) using confocal microscopy (Nikon Eclipse TE2000-E, 100×). White bars denote 25 μm scale.

We further confirmed the TRPV6 inhibitory activity of **39** by confocal microscopy of HEK-*h*TRPV6 cells preincubated with Leadmium™ Green, which is a Ca^2+^-insensitive intracellular green fluorogenic dye revealing Pb^2+^ and Cd^2+^ within the cytosol, allowing TRPV6-mediated cellular uptake independent of intracellular Ca^2+^ fluxes to be tracked. The cells were also stained with the wheat germ agglutinin Alexa Fluor™ 594 conjugate to mark the cytoplasmic membrane.^35^,^36^ In control cells (treated with the vehicle), the application of Cd^2+^ (50 μM) revealed an increase in green fluorescence after 30 min, indicating Cd^2+^ transport through *h*TRPV6 (Fig. 6A). When cells were treated with 10 μM **39** prior to the addition of Cd^2+^, the fluorescence was significantly less pronounced (Fig. 6B). Time-lapse imaging of Cd^2+^ uptake over 30 minutes furthermore showed that cells treated with inhibitor **39** had significantly reduced uptake compared to non-treated cells over the first 12 minutes, after which a plateau was reached (Fig. S2†).

**Fig. 7:**
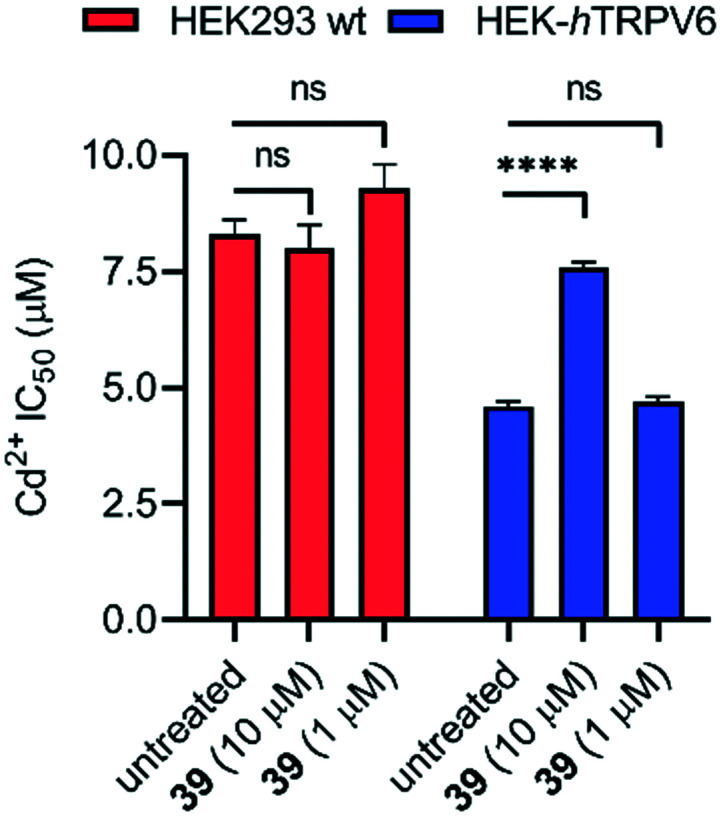
IC_50_ values of Cd^2+^ toxicity to HEK293 wt and HEK-*h*TRPV6 cells. Data shown are mean + SEM (*n* = 4/concentration) of 2 independent experiments. ****P < 0.0001; n.s., *P* > 0.05.

To assess whether pharmacological blockage of TRPV6 transport function by **39** could trigger a cellular effect, we determined the ability of **39** to reduce the Cd^2+^ toxicity towards HEK293 wt and HEK-*h*TRPV6 cell lines. Prolonged Cd^2+^ exposure is known to produce toxic effects on human cells, and eventually culminates in cell death.^37^ When we tested the viability of these cells after 24 h of treatment, we found that the Cd^2+^ dose–response in HEK-*h*TRPV6 was ∼2-fold greater than that in HEK293 wt (4.6 ± 0.1 μM and 8.3 ± 0.3 μM, respectively). These results indicate that the overexpression of *h*TRPV6 increased the transport of Cd^2+^ and consequently, the toxicity towards these cells (Fig. 7 and S3†).^38^ While **39** at 10 μM or 1 μM had no effect on the Cd^2+^ sensitivity of HEK293 wt, treating HEK-*h*TRPV6 cells with **39** at 10 μM significantly reduced the toxic effect of Cd^2+^ (Fig. 7). The reduction of Cd^2+^ toxicity occurring only in HEK-*h*TRPV6 but not in HEK293 wt indicates that **39** inhibits *h*TRPV6-mediated Cd^2+^ transport.

### 6. Ion channel selectivity and off-target profiling of pyridone **39**

**Fig. 8:**
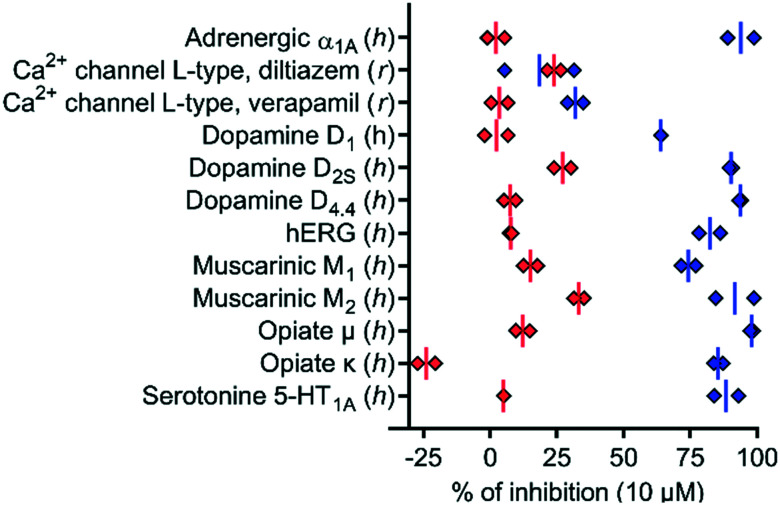
*In vitro* polypharmacology for selected targets of **1** (blue diamonds) and **39** (red diamonds). Data are shown for each replicate (*n* = 2). The data for **1** were extracted from ref. 17. The experiments were conducted by Eurofins Cerep SA, France.

**Fig. 9:**
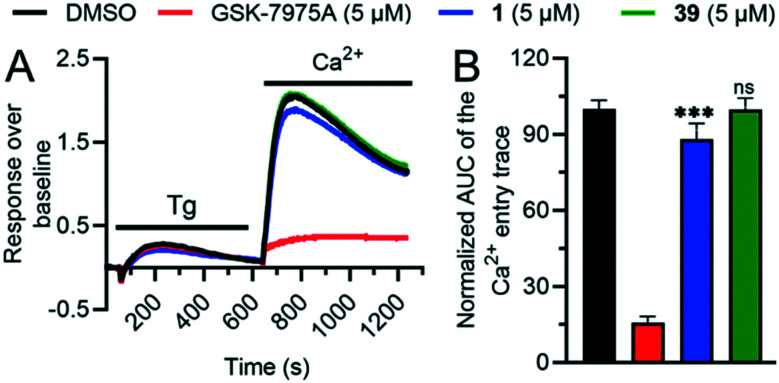
A. Recording of store-operated Ca^2+^ entry (SOCE) in MDA-MB-231 cells pretreated for 20 min with either DMSO or SOCE inhibitor GSK-7975A, or **1***vs.***39**. Ca^2+^ store-depletion was achieved by 10 min treatment of cells with 1 μM thapsigargin (Tg) in a nominally Ca^2+^ free buffer. B. SOCE quantification as the area under the curve (AUC) of the 2 mM CaCl_2_ (Ca^2+^) add-back trace is shown as normalized values to the DMSO control (mean ± SD; *n* = 18). The *P*-value of the **1***vs.***39** pretreated cells is indicated above the respective bar as *** for *p* ≤ 0.001 or non-significant (n.s.) for *p* > 0.05.

To test if the higher polarity and LLE of **39** compared to those of **1** translated into a better selectivity profile, we investigated the activity of both compounds on the closely related calcium channels. Similar to **1**, **39** showed a 7-fold selectivity for *h*TRPV6 (IC_50_ = 83 nM) against *h*TRPV5 (IC_50_ = 560 nM), low activity on L-type calcium channels at 10 μM (Fig. 8), and no detectable activity on store-operated calcium channels at 5 μM (Fig. 9). Similar to **1**,^17^**39** did not activate nor inhibit TRPV1, which is the only known TRP target of capsaicin (Fig. S2†).^39^,^40^

We also characterized the off-targets predicted to be potentially problematic for both compounds using the web-based target prediction tool PPB2.^41^ Remarkably, **39** did not show any significant off-target effects compared to **1**, indicating that its higher LLE translated into reduced polypharmacology (Fig. 8). It is worth noting that the *h*ERG activity observed with **1** was completely abolished with **39**. Furthermore, we found that the half-life in human liver microsomes, which was relatively short for **1** (*t*_1/2_ = 6 min), was significantly extended for **39** (*t*_1/2_ > 60 min), which we attribute to the replacement of the aromatic *meta*-methyl substituent with a trifluoromethyl group.^42^,^43^ Taken together, these data showed that compound **39** had a much better selectivity and stability profile compared to the original *h*TRPV6 inhibitor **1**.

### 7. Antiproliferative activity

In our initial discovery of **1**, we reported that the inhibitor significantly reduced the growth rate in T47D breast cancer cells, which express high levels of TRPV6, at micromolar concentration (IC_50_ = 25 ± 10 μM), while SKOV3 ovarian carcinoma cells, where TRPV6 expression was not detected, were less affected (IC_50_ > 50 μM).^17^ However, these values were much higher than the submicromolar levels sufficient to block TRPV6, and occurred in the range of the off-target effects of **1** (Fig. 8).

Here, we compared the effects of **1** and **39** on SKOV3 and T47D, as well as on MCF-7 and MDA-MB-231 as two additional breast cancer cell lines with low levels of TRPV6.^13^,^44^ Previous studies have shown that RNA silencing (siRNA) of TRPV6 reduces T47D cell proliferation.^11^,^13^ On the other hand, siRNA knock-down of TRPV6 does not reduce the proliferation of MCF-7 and MDA-MB-231 cells.^12^ We also investigated the non-cancer derived HEK293 immortalized cell line which does not express TRPV6,^45^,^46^ as well as the HEK-*h*TRPV6 overexpressing cell line used for activity assays.

**Fig. 10:**
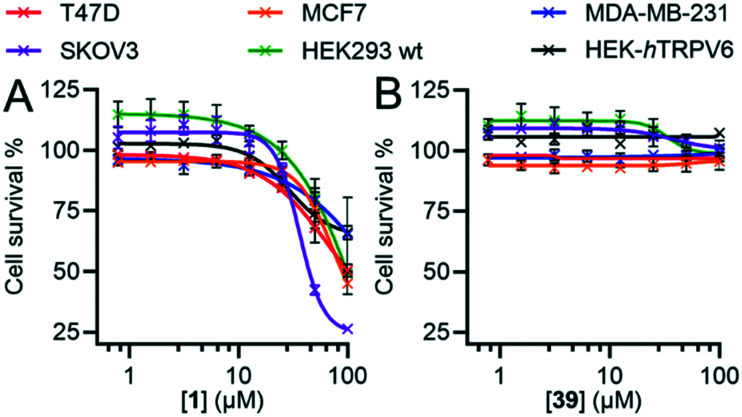
Dose–response curves of **1** (A) and **39** (B) in different breast and ovarian cancer cells and HEK293. Relative cell survival compared to the DMSO control is shown as a function of inhibitor concentration. Data shown are mean ± SEM (*n* = 8/concentration) of 3 independent experiments.

Inhibitor **1** over 6 days caused a significant reduction in cell proliferation for all six cell lines at high concentrations and not just limited to SKOV3, T47D and HEK-*h*TRPV6 cells (Fig. 10A). **1** also induced changes in cell morphology for T47D cells (Fig. S4†). By contrast, we did not observe any significant decrease in proliferation with **39** (Fig. 10B) or changes in cell morphology (Fig. S4†) under similar conditions despite its comparable IC_50_ for TRPV6 channel function inhibition. The positive control doxorubicin (10 μM), as expected, significantly decreased the growth of all six cell lines (data not shown). Note that HEK-*h*TRPV6 was more susceptible to **1** toxicity than HEK293 wt cells, which might be related to the fact that HEK-*h*TRPV6 overexpressing cells proliferate faster than HEK293 wt cells in a Ca^2+^-dependent manner (Table S3†).^46^ These results suggest that the cytotoxic effects observed with **1** but not with **39** do not reflect TRPV6 inhibition but probably result from non-specific or off-target effects. Our findings that the pharmacological inhibition of TRPV6 channel function by **39** did not affect the viability of TRPV6 expressing cell lines is intriguing. This highlights the need for future studies to uncover the precise role of TRPV6 in cancer progression.

### 8. Overview of TRPV6 inhibitor development

**Fig. 11:**
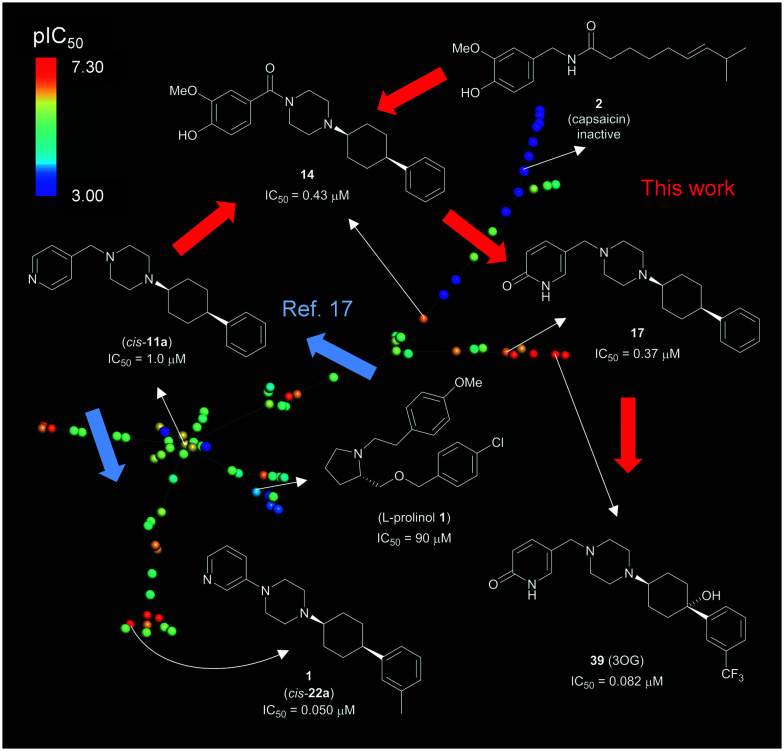
Overview of TRPV6 inhibitor development. The interactive version of the map is accessible at http://tm.gdb.tools/trpv6-inhibitors/. For color-coding, IC_50_ values were used as reported, or estimated from reported percentage inhibition at 5 μM or 10 μM. Inactive compounds were assigned IC_50_ = 1 mM. Compound numbers in parentheses are from ref. 17. The map was prepared using the public website and instructions at ; https://try-tmap.gdb.tools/.

The overall development of our inhibitors is illustrated here with an interactive tree-map (TMAP)^47^ representing each molecule as a point color-coded by TRPV6 inhibitory potency (Fig. 11). In this map, molecules are connected by a minimum spanning tree to their most similar analogs as measured by the extended connectivity fingerprint MHFP6.^48^

The lower left portion of the TMAP illustrates our initial study (blue arrows),^17^ in which we searched for scaffold-hopping analogues of known, weakly active TRPV6 inhibitors including an l-prolinol derivative. We discovered a first hit compound (*cis*-**11a**), which we optimized to **1** (*cis*-**22a**). The upper right branch contains capsaicin and its analogs **41–48**, which were inactive. This branch also contains **14** which combines elements from capsaicin and *cis*-**22a**. The further optimization of **14** by introducing a pyridone to form **17** and its further optimization to inhibitor **39** (3OG) appears as an additional side branch.

Throughout these explorations, we found that a (4-arylcyclohexyl)-piperazine with *cis*-stereochemistry on the cyclohexane was critical to give strong TRPV6 inhibition. On the other hand, TRPV6 inhibition was compatible with variations in the second piperazine substituent and to a certain extent with substitutions on the aromatic cyclohexane substituent. This suggests that further improvements in inhibitory potency and in compound properties might be achievable with further variations at these positions.

## Conclusions

To improve the properties of the previously reported TRPV6 inhibitor **1**, we surveyed analogues incorporating structural features inspired by the natural product capsaicin such as aliphatic and oxygen-containing functional groups. Although we found that, contrary to previous reports, capsaicin does not have any inhibitory effect on TRPV6, our strategy led us to identify the new inhibitor **39** (3OG), which incorporates a pyridone group and a tertiary alcohol as typical natural product-like features. Inhibitor **39** shows similar potency against TRPV6 and ion channel selectivity to **1** but much better microsomal stability and much lower off-target effects, in particular suppressed *h*ERG inhibition. Inhibitor **39** blocks TRPV6 transport function in cells as assessed by the reduction of Cd^2+^ toxicity in HEK-*h*TRPV6. However, even at high concentration, **39** does not display any measurable cellular toxicity on various cell lines, expressing TRPV6 or not. Structural and mutagenesis studies based on the recently published structure of *h*TRPV6 (ref. 3 and 49) and showing how **1** and **39** inhibit TRPV6 will be reported in the near future. This new tool compound should be useful to decipher the role of TRPV6 mediated calcium flux in various disease models.

## Conflicts of interest

There are no conflicts to declare.

## Supplementary Material

Supplementary information

Crystal structure data
