# Supplementary material for: Natural product inspired optimization of a selective TRPV6 calcium channel inhibitor
Source: RSC Med Chem. 2020 Jul 16;11(9):1032–40. doi: 10.1039/d0md00145g (PMC7513592; doi:10.1039/d0md00145g)
Supplement: Supplementary file 1 [file MD-011-D0MD00145G-s001.pdf]

Electronic Supplementary Material (ESI) for RSC Medicinal Chemistry.
This journal is © The Royal Society of Chemistry 2020

Supplementary Information

Natural Product Inspired Optimization of a Selective TRPV6 Calcium Channel Inhibitor

Micael R. Cunha,[a,b] Rajesh Bhardwaj,[c] Aline L. Carrel,[a] Sonja Lindinger,[d] Christoph Romanin,[d] Roberto Parise-Filho,[b]* Matthias A. Hediger,[c]* Jean-Louis Reymond.[a]*

[a]Department of Chemistry and Biochemistry, University of Bern, Freiestrasse 3, 3012 Bern, Switzerland.

[b]Department of Pharmacy, University of São Paulo, Prof. Lineu Prestes Avenue 580, 05508-000 São Paulo, Brazil.

[c]Department of Nephrology and Hypertension, University Hospital Bern, Inselspital, 3010 Bern, Switzerland.

[d]Institute of Biophysics, Johannes Kepler University, 4020 Linz, Austria.

*To whom correspondence should be addressed. (R.P.F.) E-mail: roberto.parise@usp.br, Telephone +55 11 3091 3793. (M.A.H.) E-mail: matthias.hediger@ibmm.unibe.ch, Telephone . +41 31 632 94 39. (J.L.R.) E-mail: jean-louis.reymond@dcb.unibe.ch. Telephone +41 31 631 43 25.

Abbreviations: ADMET, Absorption, Distribution, Metabolism, Excretion, Toxicity; Akt, Protein Kinase B; DMEM, Dulbecco's Modified Eagle Medium; ESI, Electron Spray Ionization; FBS, Fetal Bovine Serum; FLIPR, Fluorescent Imaging Plate Reader; HA Heavy Atom; HEK293, Human Embryotic Kidney; *h*ERG, Human Ether-a-Go-Go-Related Gene; IC_{50}, Half Maximal Inhibitory Concentration; IGF1R, Insulin-Like Growth Factor 1 Receptor; LE, Ligand Efficiency; LLE, Lipophilic Ligand Efficiency; mRNA, Messenger Ribonucleic Acid; NFAT, Nuclear Factor of Activated T-Cells; NMR, Nuclear Magnetic Resonance; NCF, Nominally Calcium Free; PI3K, Phosphoinositide 3-Kinase; PMS, Phenazine Methosulfate; PPTS, Pyridinium *Para*-Toluenosulfonate; RP-UHPLC, Reversed Phase Ultra High Performance Liquid Chromatography; SAR, Structure-Activity Relationship; TBDMS, tert-Butyldimethylsilyl; TBAF, Tetrabutylammonium Fluoride; TFA, Trifluoroacetic Acid; TRPV, Transient Receptor Potential Vanilloid channels; TLC, Thin Layer Chromatography; TNH, Transient Neonatal Hyperparathyroidism; TRPA, Transient Receptor Potential Cation channels; TRPC, Transient Receptor Potential Canonical channels; TRPM, Transient Receptor Potential Melastatin channels; UV, Ultraviolet.

Table of contents

1. **Chemical synthesis** **3**

2. **Copy of ^{1}H and ^{13}C NMR spectra** **13**

3. **X-Ray crystal deposition** **57**

4. **Analytical purity for tested compounds** **58**

5. **TRPV6 FLIPR assay** **59**

6. **TRPV5 FLIPR assay** **60**

7. **TRPV1 FLIPR assay** **61**

8. **Electrophysiology** **62**

9. **SOCE FLIPR assay** **63**

10. **Confocal microscopy** **64**

11. **Cadmium toxicity** **65**

12. **Antiproliferative activity** **66**

13. **References** **68**

List of figures

Fig S1. X-ray crystal structures of **1** (CCDC 1997204)**, 9** (CCDC 1997203) **19** (CCDC 1997202)**, 31** (CCDC 1997201)**,** and **40** (CCDC 1997205) shown as ORTEP with ellipsoids drawn at the 50% probability level. Hydrogen atoms (white spheres, arbitrary radius) were located in the difference Fourier map and refined freely. CCDC codes contains the supplementary crystallographic data for this paper. 57

Fig S2. Average recording of Ca^{2+} entry in HEK-hTRPV1 cells pretreated for 5 min with either DMSO, capsazepine (CPZ) or **39**. Data shown is mean (n = 3) of a single experiment. Ca^{2+} influx was achieved by 10 min treatment of cells with 100 nM capsaicin in 1.8 mM $CaCl_2$ containing Krebs buffer. Quantification reveals that 10 µM of **39** inhibited 2% the Ca^{2+} influx through capsaicin-activated hTRPV1. The positive control CPZ inhibited 100% at 10 µM. 61

Fig S3. Time-course of Cd^{2+} uptake in HEK-hTRPV6 cells with Leadmiun Green (LG), wheat germ agglutinin Alexa Fluor® 594 conjugate (AF), and Hoechst 33258 (H) along 30 min. Images were collected using confocal microscopy (Nikon Eclipse TE2000-E, 100X). HEK-hTRPV6 cells were incubated with fluorescent dyes for 30 min at 37 °C. To these cells DMSO (A) or **39** (10 µM, C) was applied followed by a solution of Cd^{2+} (50 µM). The total fluorescence intensity for each channel was plotted in graphs (B) and (D), respectively for DMSO and compound **39**. Images were collected with excitation for LG at λ_{ex} = 488 nm and emission at λ_{em} = 520 nm, H at λ_{ex} = 352 nm and emission at λ_{em} = 461 nm, and AF at λ_{ex} = 590 nm and emission at λ_{em} = 617 nm. White bars denote 20 µm. 64

Fig S4. XTT cell viability curve of HEK293 wt and HEK-hTRPV6 under Cd^{2+} in presence or not of **39** (1.0 and 10 µM). Data shown represent each replicate (n = 4/concentration) from 2 independent experiments. 65

Fig S5. Photomicrographs show the morphological aspects of the control cultures of T47D cells (A), and treated with **1** (100 µM, B); **39** (100 µM, C); doxorubicin (10 µM, D). Cells were imaged at 20X with an inverted microscope (Nikon Eclipse TiU). 67

List of tables

Table S1. Analytical RP-UHPLC purity compounds **6-40** 58

Table S2. Analytical RP-HPLC purity of compounds 41-46 58

Table S3. IC_{50} activity of **1** and **39** against several breast cancer and HEK293 cell lines 66

1. Chemical synthesis

Chemistry. *General Methods*. All commercial reagents were used without further purification. Dry solvents were obtained directly from a drying solvent system. Chromatographic purifications were performed using silica gel (Sigma-Aldrich, 230-400 mesh). Automated chromatographic purification was performed with Puriflash 430 system (Interchim) using Teledyne Isco normal phase RediSepRf cartridge and detection by UV absorption (214 nm). High-resolution mass spectra were obtained electron spray ionization (ESI), positive mode (Thermo Scientific LTQ OritrapXL or MicroToF Bruker Daltonics). Preparative RP-HPLC was performed with Waters Prep LC4000 Chromatography System using a Reprospher 100 (C18-DE, 100 mm x 30 mm, particle size 5 µM, 100 Å pore size) column from Dr. Maisch GmbH and a Waters 489 Tunable Absorbance Detector operating at 214 nm. ^{1}H, ^{13}C, and ^{19}F-NMR spectra were recorded at 300MHz, 75MHz, and 376MHz respectively (Bruker AVANCE III HD 300 or DPX-300). For very small amounts, ^{1}H and ^{13}C-NMR spectra were recorded at 400MHz and 100MHz, respectively (Bruker AVANCE II 400). Chemical shifts are quoted relative to solvent signals. MestreNova was used for further analysis of the spectra. The following abbreviations for multiplicities were used: s = singlet, d = doublet, t = triplet, q = quartet, p = pentet, m = multiplet, dd = doublet of doublets, dt = doublet of triplets, tt = triplet of triplets, and br s = broad singlet. TLC plates (Merck silica gel 60 F_{254}) were used to monitor the reaction progress, and spots were visualized under UV (254 nm). The purity of all tested compounds was > 95% (Table S1, S2). The chromatographic purity of the bioisosteric capsaicinoids was determined using a High-Performance Liquid Chromatograph (Shimadzu®-PROMINENCE) coupled to a C18 column (Waters®-µBondpak C18, 3.9 x 300 mm). For the hybrid capsaicinoids the purity was confirmed by analytical RP-UHPLC with detection at 214nm, on a Dionex Ultimate 3000 RSLC System (DAD-3000 RS Photodiode Array Detector) and Dionex Acclaim RSLC 120 column (C18, 3.0 x 50 mm, particle size 2.2 µm, 120 Å pore size) at a flow rate of 1.2 mL/min. Data recording and processing was done with Dionex Chromelon Management System (v. 6.8), and Xcalibur (v. 2.2, Thermo Scientific). Eluents for analytical HPLC were as follows: A: miliQ-deionized water with 0.05% TFA and D: HPLC-grade acetonitrile with 0.05% TFA. Conditions for analytical HPLC were as follow: the flow stays in 90% A and 10% D for 4.0 min, then in 25 min from 90% A and 10 % D to 0 % and 100 % D, then staying on 100% D. Eluents for analytical and preparative RP-UHPLC were as follow: A: miliQ-deionized water with 0.05% TFA and D: HPLC-grade acetonitrile/miliQ-deionized water (9/1) with 0.05% TFA. Conditions for analytical RP-UHPLC were as follow: in 4.5 min from 100% A to 100% D, then staying on 100% D, or in 7.5 min from 100% A to 100% D, then staying on 100% D. Conditions for preparative RP-UHPLC were described after compound characterization. Chemical names were generated using ChemDraw Professional 17.0 (PerkinElmer Informatics).

4-((tert-butyldimethylsilyl)oxy)-3-methoxybenzaldehyde (**5**). In a round-bottom flask containing a solution of **4** (2.1 g, 1 eq.), imidazole (1,9 g, 2 eq.), and DMAP (84.3 mg, 0.05 eq.) in DCM (50 mL) was added dropwise a solution of tert-butyldimethylsilyl chloride (2.5 g, 1.2 eq.) in DCM (50 mL). The reaction mixture was stirred for 2 h, at r.t. The reaction was quenched by the addition of saturated solution of NH_4Cl (50.0 mL). The organic phase was extracted. Another 50.0 mL of saturated solution of NH_4Cl was added and extracted. The organic phase was washed with Brine (2 x 50.0 mL) and water (2 x 50.0 mL) and dried over $MgSO_4$. The solvent was evaporated under vacuum and the crude was column chromatographed (hexanes:EtOAc, 9:1) to afford the desired compound as a colorless oil (3.7 g, quant.). ^{1}H NMR (300 MHz, $CDCl_3$): δ 9.65 (s, 1H), 7.21 (d, *J* = 1.9 Hz, 1H), 7.17 (dd, *J* = 8.0, 1.9 Hz, 1H), 6.77 (d, *J* = 7.9 Hz, 1H), 3.67 (s, 3H), 0.81 (s, 9H), -0.00 (s, 6H).^{13}C NMR (75 MHz, $CDCl_3$): δ 191.1, 151.8, 151.5, 131.1, 126.3, 120.8, 110.2, 55.5, 25.7, 18.6, -4.5.

4-((4-cyclohexylpiperazin-1-yl)methyl)-2-methoxyphenol (**6**). General procedure A: In a round-bottom flask containing a solution of **5** (2.7 g, 1 eq.), N_1-Boc-piperazine (2 g, 1.1 eq.) and AcOH (100 µL) in dry DCE (50 mL) was added $NaBH(OAc)_3$ (2.8 g, 1.3 eq.) and the reaction mixture was stirred for 48h h, at r.t. After completion (TLC), the reaction mixture was evaporated under vacuum. The crude was re-suspended in DCM for silica loading. After column chromatography (hexanes:EtOAc, 9:1 to 5:5 + 0.5% Et_3N) the intermediate was solubilized in a mixture of DCM/TFA (1:1; 40 mL) and was stirred for 90 min, at r.t. The solvents were removed under vacuum. The crude compound was solubilized in EtOAc and precipitated by the addition of dry Et_2O to afford the desired free-piperazine compound as a white solid, used for the synthesis of **6-9** (2.5 g, 45 %). ^{1}H NMR (300 MHz, CD_3OD): δ 7.12 (d, *J* = 1.2 Hz, 1H), 6.95 (dd, *J* = 8.0, 1.4 Hz, 1H), 6.89 (d, *J* = 8.0 Hz, 1H), 4.25 (s, 2H), 3.83 (s, 3H), 3.48 (dd, *J* = 27.9, 4.1 Hz, 8H), 1.00 (s, 9H), 0.15 (s, 6H).^{13}C NMR (75 MHz, $CDCl_3$): δ 152.8, 147.8, 124.9, 124.1, 122.1, 115.5, 61.9, 55.9, 42.4, 26.1, 19.3, -

4.5. In a round-bottom flask containing a solution of the previously obtained free-piperazine compound (311 mg, 1.1 eq.) in dry DCE (5 mL) was added Et_3N (280 µL, 4 eq.). After 15 min, cyclohexanone (52 µL, 1 eq.) and $NaBH(OAc)_3$ (144 mg, 1.3 eq.) were added, and the mixture was stirred for 24 h, at r.t. The reaction mixture was evaporated under vacuum. The crude was re-suspended in DCM and was column chromatographed (hexanes:EtOAc, 5:5 + 0.5% Et_3N) afforded the protected intermediate, used in the next step without further purification. To the previously obtained compound, a solution of TBAF (in THF, 1.1 mL, 1 M) was added dropwise, and the reaction was stirred for 3 h, at r.t. Upon completion, the reaction was quenched by the addition of saturated solution of $NaHCO_3$ (3.0 mL) and was extracted with EtOAc (2 x 10.0 mL). The collected organic phase was washed with Brine (2 x 10.0 mL) and water (2 x 10.0 mL), and dried over Na_2SO_4. The solvent was removed under vacuum. After work-up, the crude was solubilized in MeOH/HCl (3.0 M) and was precipitated by the addition of dry Et_2O as a white powder (140 mg, 37 %). ^{1}H NMR (300 MHz, D_2O): δ 7.15 (d, *J* = 1.5 Hz, 1H), 7.02 (m, 2H), 4.39 (s, 2H), 3.90 (s, 3H), 3.44 (m, 9H), 2.12 (d, *J* = 11.1 Hz, 2H), 1.92 (d, *J* = 12.9 Hz, 2H), 1.67 (m, 1H), 1.42 (m, 4H), 1.15 (m, 1H).^{13}C NMR (75 MHz, D_2O): δ 147.8, 146.8, 124.8, 119.8, 115.9, 114.9, 66.3, 60.4, 56.0, 48.1, 45.5, 26.5, 24.3, 24.2. HRMS m/z calculated for $C_{18}H_{29}N_2O_2$: 305.2224 $[M+H]^+$; found, 305.2220.

4-((4-((1s,4s)-4-ethylcyclohexyl)piperazin-1-yl)methyl)-2-methoxyphenol (**7**). **7** was synthesized by reacting the previously obtained free-piperazine compound (311 mg, 1.1 eq.) and 4-ethyl-cyclohexanone (71 µL, 1 eq.) following the general procedure A and was obtained as a white powder (119 mg, 59 %). ^{1}H NMR (300 MHz, CD_3OD): δ 7.15 (d, *J* = 2.0 Hz, 1H), 6.90 (dd, *J* = 8.1, 2.0 Hz, 1H), 6.77 (d, *J* = 8.1 Hz, 1H), 4.29 (s, 2H), 3.82 (s, 3H), 3.76-3.49 (m, 9H), 2.47 (d, *J* = 2.6 Hz, 4H), 1.85-1.47 (m, 9H), 1.33 (p, *J* = 7.3 Hz, 2H), 0.82 (t, *J* = 7.4 Hz, 3H).^{13}C NMR (75 MHz, D_2O): δ 147.8, 146.8, 124.8, 119.8, 115.9, 114.9, 66.3, 60.4, 56.0, 48.0, 45.8, 33.2, 27.0, 23.3, 21.7, 11.3.HRMS m/z calculated for $C_{20}H_{33}N_2O_2$: 333.2537 $[M+H]^+$; found, 333.2532.

4-((4-((1s,4s)-4-(tert-butyl)cyclohexyl)piperazin-1-yl)methyl)-2-methoxyphenol (**8**). **8** was synthesized by reacting the previously obtained free-piperazine compound (311 mg, 1.1 eq.) and 4-tert-butyl-cyclohexanone (77 mg, 1 eq) following general procedure A and was purified by RP-UHPLC (from 75%A25%D to 65%A35%D in 30 min; RT_{7min}: 2.33 min) to be afforded as white powder (89 mg, 30 %). ^{1}H NMR (300 MHz, C_6D_6): δ 7.03 (d, *J* = 8.0 Hz, 1H), 6.88 (d, *J* = 1.7 Hz, 1H), 6.81 (dd, *J* = 8.0, 1.8 Hz, 1H), 3.34 (s, 2H), 3.19 (2, 3H), 2.45 (s, 8H), 2.08 (t, *J* = 8.0 Hz, 1H), 1.95 (d, *J* = 14.6 Hz, 2H), 1.54-1.34 (m, 4H), 1.19 (tt, *J* = 13.4, 2.9 Hz, 2H), 1.04 (tt, *J* = 11.7, 3.7 Hz, 1H), 0.92 (s, 9H). ^{13}C NMR (75 MHz, C_6D_6): δ 147.1, 145.7, 130.8, 122.5, 114.4, 111.8, 63.5, 58.4, 55.2, 54.1, 50.5, 48.8, 32.8, 29.6, 27.8, 21.6. HRMS m/z calculated for $C_{22}H_{37}N_2O_2$: 361.2850 $[M+H]^+$; found, 361.2843.

2-methoxy-4-((4-((1s,4s)-4-phenylcyclohexyl)piperazin-1-yl)methyl)phenol (**9**). **9** was synthesized by reacting the previously obtained free-piperazine compound (311 mg, 1.1 eq.) and 4-phenyl-cyclohexanone (87 mg, 1 eq.) following general procedure A, and was purified by RP-UHPLC (from 75%A25%D to 65%A35%D in 30 min; RT_{7min}: 3.66 min) to be afforded as a white powder (151 mg, 50 %). ^{1}H NMR (300 MHz, C_6D_6): δ 7.25 (ddd, *J* = 15.0, 10.7, 4.7 Hz, 4H), 7.10 (tt, *J* = 7.0 Hz, 1H), 7.04 (d, *J* = 8.0 Hz, 1H), 6.89 (d, *J* = 1.7 Hz, 1H), 6.82 (dd, *J* = 8.0, 1.7 Hz, 1H), 3.37 (s, 2H), 3.20 (s, 3H), 2.51-2.45 (m, 9H), 2.11 (t, *J* = 2.9 Hz, 1H), 2.05-1.87 (m, 4H), 1.55-1.50 (m, 2H), 1.35-1.25 (m, 2H). ^{13}C NMR (75 MHz, C_6D_6): δ 147.9, 147.1, 145.8, 130.7, 128.7, 127.4, 126.2, 122.5, 114.5, 111.8, 63.5, 58.8, 55.2, 54.1, 50.4, 44.4, 28.9, 28.7. HRMS m/z calculated for $C_{24}H_{33}N_2O_2$: 381.2537 $[M+H]^+$; found, 381.2526.

(4-cyclohexylpiperazin-1-yl)(4-hydroxy-3-methoxyphenyl)methanone (**11**). General procedure B: In a round-bottom flask was added 4-hydroxy-3-methoxybenzoic acid (**10**, 841 mg, 1 eq.), N_1-Boc-piperazine (1 g, 1.1 eq), EDCl (1 g, 1.1 eq) and DMAP (672 mg, 1.1 eq.) in DCM (50 mL). The mixture was stirred for 17 h at r.t. until all the reagents were solubilized. Upon completion, the reaction mixture was evaporated under vacuum and the crude was re-suspended in DCM for silica loading. Column chromatography (Hexanes:EtOAc, 5:5) afforded the Boc-protected intermediate, used in the next step without further purification. The obtained compound (1 g, 1 eq.) was added to a round-bottom flask and was suspended in an aqueous solution of HCl (1 M). The suspension was refluxed for 2 h, then the solvent was removed under vacuum. The gummy residue was solubilized in a minimal amount of water and lyophilized to afford the desired free-piperazine compound as a white powder, used for the synthesis of **11-14** (822 mg, 60%). ^{1}H NMR (300 MHz, D_2O): δ 7.06 (d, *J* = 1.5 Hz, 1H), 6.98-6.90 (m, 2H), 3.84 (s, 7H), 3.58 (s, 2H), 3.31 (s, 4H).^{13}C NMR (75 MHz, D_2O): δ 172.5, 147.4, 147.4, 125.3, 120.9, 115.3, 111.4, 56.0, 43.0, 40.3. In a round-bottom flask containing a solution of the previously obtained free-piperazine compound (108 mg, 1.1 eq.)

in dry DCE (5 mL) was added Et_3N (195 µL, 4 eq.). After 15 min, cyclohexanone (37 µL, 1 eq.) and $NaBH(OAc)_3$ (101 mg, 1.3 eq.) were added, and the mixture was stirred for 48 h, at r.t. Upon completion, the solvent was removed under vacuum. The compound was purified by RP-UHPLC (from 90%A10%D to 60%A40%D in 20 min; RT_{7min}: 1.47 min) and was afforded as a white powder (54 mg, 31 %). ^1H NMR (300 MHz, D_2O): δ 7.09 (s, 1H), 7.01-6.95 (m, 2H), 4.12 (bs, 1H), 3.86 (s, 3H), 3.54-3.18 (m, 7H), 2.08 (d, *J* = 10.9 Hz, 2H), 1.90 (d, *J* = 12.8 Hz, 2H), 1.66 (d, *J* = 12.6 Hz, 1H), 1.52-1.26 (m, 4H), 1.21-1.08 (m, 1H). ^{13}C NMR (75 MHz, D_2O): δ 172.3, 147.5, 125.1, 121.0, 115.3, 111.5, 66.1, 56.0, 48.0, 26.6, 24.4. HRMS m/z calculated for $C_{18}H_{27}N_2O_3$: 319.2016 $[M+H]^+$; found, 319.2022.

(4-((1s,4s)-4-ethylcyclohexyl)piperazin-1-yl)(4-hydroxy-3-methoxyphenyl)methanone (**12**). **12** was synthesized by reacting the previously obtained free-piperazine compound (150 mg, 1.1 eq.) and 4-ethyl-cyclohexanone (71 µL, 1 eq.) following general procedure B, and was obtained as a white powder (41 mg, 21 %). ^1H NMR (300 MHz, CD_3OD): δ 7.09 (d, *J* = 1.9 Hz, 1H), 7.00 (dd, *J* = 8.1, 1.9 Hz, 1H), 6.87 (d, *J* = 8.1 Hz, 1H), 4.42 (s, 2H), 3.89 (s, 3H), 3.61 (d, *J* = 12.4 Hz, 2H), 3.50 (t, *J* = 13.0 Hz, 2H), 3.20 (ddd, *J* = 24.4, 11.7, 3.1 Hz, 3H), 1.95-1.91 (m, 2H), 1.83-1.58 (m, 7H), 1.44 (q_i, *J* = 7.3 Hz, 2H), 0.93 (t, *J* = 7.4 Hz, 3H). ^{13}C NMR (75 MHz, CD_3OD): δ 172.7, 150.5, 149.2, 126.1, 122.2, 116.1, 112.6, 67.5, 56.6, 35.1, 28.9, 24.7, 23.0, 12.4. HRMS m/z calculated for $C_{20}H_{31}N_2O_3$: 347.2329 $[M+H]^+$; found, 347.2316.

(4-((1s,4s)-4-(tert-butyl)cyclohexyl)piperazin-1-yl)(4-hydroxy-3-methoxyphenyl)methanone (**13**). **13** was synthesized by reacting the previously obtained free-piperazine compound (150 mg, 1.1 eq.) and 4-tert-butyl-cyclohexanone (77 mg, 1 eq.) following general procedure B, as was obtained a white powder (53 mg, 26 %). ^1H NMR (300 MHz, CD_3OD): δ 7.10 (d, *J* = 1.8 Hz, 1H), 7.00 (dd, *J* = 8.1, 1.9 Hz, 1H), 6.87 (d, *J* = 8.1 Hz, 1H), 4.35 (s, 2H), 3.89 (s, 3H), 3.76-3.63 (m, 4H), 3.40 (t, *J* = 3.5 Hz, 1H), 3.15 (td, *J* = 12.0, 3.0 Hz, 2H), 2.28 (d, *J* = 14.0 Hz, 2H), 1.82-1.72 (m, 4H), 1.48-1.34 (m, 2H), 1.23 (tt, *J* = 11.2, 3.1 Hz, 1H), 0.92 (s, 9H). ^{13}C NMR (75 MHz, CD_3OD): δ 172.7, 150.6, 149.2, 126.1, 122.2, 116.1, 112.6, 65.0, 56.6, 51.2, 33.5, 28.0, 26.8, 22.6. HRMS m/z calculated for $C_{22}H_{35}N_2O_3$: 375.2642 $[M+H]^+$; found, 375.2626.

(4-hydroxy-3-methoxyphenyl)(4-((1s,4s)-4-phenylcyclohexyl)piperazin-1-yl)methanone (**14**). **14** was synthesized by reacting the previously obtained free-piperazine compound (150 mg, 1.1 eq.) and 4-phenyl-cyclohexanone (87 mg, 1 eq.) following general procedure B, and was obtained as a white powder (52 mg, 24 %). ^1H NMR (300 MHz, CD_3OD): δ 7.40 (d, *J* = 7.5 Hz, 2H), 7.31 (t, *J* = 7.6 Hz, 2H), 7.18 (t, *J* = 7.2 Hz, 1H), 7.08 (d, *J* = 1.7 Hz, 1H), 6.99 (dd, *J* = 8.1, 1.8 Hz, 1H), 6.86 (d, *J* = 8.1 Hz, 1H), 4.37 (s, 2H), 3.88 (s, 3H), 3.67-3.53 (m, 4H), 3.42 (s, 1H), 3.15 (td, *J* = 12.1, 3.0 Hz, 2H), 2.98 (s, 1H), 2.34-2.26 (m, 2H), 2.06-1.85 (m, 6H). ^{13}C NMR (75 MHz, CD_3OD): δ 172.7, 150.5, 149.1, 144.9, 129.6, 128.3, 127.1, 126.1, 122.2, 116.1, 112.6, 66.4, 56.6, 49.6, 39.3, 29.1, 25.0. HRMS m/z calculated for $C_{24}H_{31}N_2O_3$: 395.2329 $[M+H]^+$; found, 395.2312.

5-((4-((1s,4s)-4-(tert-butyl)cyclohexyl)piperazin-1-yl)methyl)pyridin-2(1H)-one (**16**). In a round-bottom flask containing a solution of 6-hydroxy-nicotinaldehyde (**15**, 616 mg, 1 eq.) in dry DCE (50 mL) was added N_1-Boc-piperazine (1 g, 1.1 eq.). After 5 min stirring, $NaBH(OAc)_3$ (1.3 g, 1.3 eq.) was added, and the mixture was stirred for 48 h, at r.t. Upon completion, the reaction mixture was diluted with DCM (25 mL) and was extracted with an aqueous solution of HCl (3 x 25mL; 1.0 M). The collected aqueous phase was basified with an aqueous saturated solution of $NaHCO_3$ and extracted with EtOAc (2 x 25.0 mL). The organic phase was dried over Na_2SO_4 and evaporated under vacuum to yield the intermediate as white solids, used in the next step without further purification. The compound (1.1 g, 1 eq.) was solubilized in a mixture of DCM: TFA (1:1, 20 mL) and was stirred at r.t. for 90 min. The reaction mixture was then evaporated and the residue was solubilized in water (50.0 mL) and lyophilized to afford the desired compound as a white powder, used for the synthesis of **16** and **17** (1.6 g, 78 %). ^1H NMR (300 MHz, D_2O): δ 7.82-7.78 (m, 1H), 6.72 (dd, *J* = 2.0, 4.1, 10.2 Hz, 2H), 4.37 (s, 2H), 3.64 (s, 8H), 3.60 (s, 1H). ^{13}C NMR (75 MHz, D_2O): δ 164.5, 144.8, 138.8, 120.0, 117.5, 108.7, 57.1, 47.7, 40.7, 40.3. In a round-bottom flask containing a solution of the previously obtained free-piperazine compound (147 mg, 1.2 eq.) in dry DCE (5 mL) was added Et_3N (280 µL, 4 eq.). After 15 min, 4-tert-butyl-cyclohexanone (77 mg, 1 eq.) and $NaBH(OAc)_3$ (144 mg, 1.3 eq.) were added, and the mixture was stirred for 24 h, at r.t. The reaction mixture was evaporated under vacuum. The crude was purified by RP-UHPLC (from 75%A25%D to 70%A30%D in 30 min; RT_{7min}: 1.61 min) to be afforded as a white powder (43 mg, 15 %). ^1H NMR (300 MHz, C_6D_6): δ 7.19 (d, *J* = 2.3 Hz, 1H), 6.92 (s, 1H), 6.59 (d, *J* = 9.3 Hz, 1H), 2.76 (s, 2H), 2.43 (s, 4H), 2.19 (s, 4H), 2.05 (d, *J* = 7.2 Hz, 2H), 1.94 (d, *J* = 14.1 Hz, 2H), 1.51-1.36 (m, 4H), 1.21 (t, *J* = 13.0 Hz, 1H), 1.09-1.00 (m, 1H), 0.92

(s, 9H). ^{13}C NMR (75 MHz, C_6D_6): δ 165.7, 143.4, 133.9, 120.2, 117.2, 58.9, 58.3, 53.6, 50.3, 48.7, 32.8, 29.5, 27.8, 21.6. HRMS m/z calculated for $C_{20}H_{34}N_3O$: 332.2696 $[M+H]^+$; found, 332.2682.

5-((4-((1s,4s)-4-phenylcyclohexyl)piperazin-1-yl)methyl)pyridin-2(1H)-one (**17**). **17** synthesized by reacting the previously obtained free-piperazine compound (147 mg, 1.2 eq.) and 4-phenyl-cyclohexanone (87 mg, 1 eq.) as in **17**, and was obtained as a white powder (95 mg, 45 %). ^1H NMR (300 MHz, C_6D_6): δ 14.26 (s, 1H), 7.30-7.18 (m, 5H), 7.10 (tt, *J* = 7.0, 1.5 Hz, 1H), 6.89 (d, *J* = 2.0 Hz, 1H), 6.61 (d, *J* = 9.3 Hz, 1H), 2.77 (s, 2H), 2.54 (tt, *J* = 11.0, 3.6 Hz, 1H), 2.33 (s, 4H), 2.20 (s, 4H), 2.09 (s, 1H), 2.05-1.87 (m, 4H), 1.57-1.52 (m, 2H), 1.32 (t, *J* = 12.9 Hz, 2H). ^{13}C NMR (75 MHz, C_6D_6): δ 165.8, 147.8, 143.2, 134.1, 128.7, 127.3, 126.2, 120.4, 116.9, 59.1, 58.7, 53.6, 50.3, 44.3, 28.9, 28.7. HRMS m/z calculated for $C_{22}H_{30}N_3O$: 352.2383 $[M+H]^+$; found, 352.2372.

5-(4-((1s,4s)-4-(tert-butyl)cyclohexyl)piperazine-1-carbonyl)pyridin-2(1H)-one (**19**). In a round-bottom flask was added 6-hydroxynicotinic acid (**18**, 696 mg, 1 eq.), N_1-Boc-piperazine (1 g, 1.1 eq), EDCl (1 g, 1.1 eq) and DMAP (672 mg, 1.1 eq.) in DCM (50 mL). The mixture was stirred for 17 h at r.t. until all the reagents were solubilized. Upon completion, the reaction mixture was washed with an aqueous solution of HCl (3 x 254 mL; 1.0 M). The resulting organic phase was dried over $MgSO_4$ and evaporated. The obtained intermediate was used in the next step without further purification. The compound (1.0 g, 1 eq.) was dissolved in a mixture of DCM (25.0 mL) and TFA (25.0 mL) and was stirred at r.t., for 90 min. The reaction mixture was cooled in an ice bath and H_2O (50.0 mL) was added to it. The aqueous phase was extracted and washed with DCM (2 x 50.0 mL). The collected aqueous phase was partially evaporated under vacuum and was lyophilized to yield the desired compound as a white powder, used for the synthesis of **19** and **20** (1.1 g, 70%). %). ^1H NMR (300 MHz, D_2O): δ 7.81 (d, *J* = 2.2 Hz, 1H), 7.72 (dd, *J* = 9.4, 2.6 Hz, 1H), 6.65 (d, *J* = 9.4 Hz, 1H), 3.88 (t, *J* = 10.6, 5.2 Hz, 4H), 3.32 (t, *J* = 10.6, 5.2 Hz, 4H). ^{13}C NMR (75 MHz, D_2O): δ 168.6, 164.5, 141.7, 136.7, 119.2, 114.6, 42.8. In a round-bottom flask containing a solution of the previously obtained free-piperazine compound (177 mg, 1.1 eq.) in dry DCE (5 mL) was added Et_3N (280 µL, 4 eq.). After 15 min, 4-tert-butyl-cyclohexanone (77 mg, 1 eq.) and $NaBH(OAc)_3$ (144 mg, 1.3 eq.) were added, and the mixture was stirred for 24 h, at r.t. The reaction mixture was evaporated under vacuum. The crude was purified by RP-UHPLC (from 75%A25%D to 60%A40%D in 30 min; RT_{7min}: 1.70 min) to be afforded as a white powder (16 mg, 6 %). ^1H NMR (300 MHz, CD_3OD): δ 7.78 (d, *J* = 2.1 Hz, 1H), 7.70 (dd, *J* = 9.5, 2.6 Hz, 1H), 6.56 (d, *J* = 9.4 Hz, 1H), 4.31 (s, 2H), 3.67 (d, *J* = 45.8 Hz, 4H), 3.36 (t, *J* = 3.5 Hz, 1H), 3.13 (s, 2H), 2.28 (d, *J* = 15.5 Hz, 2H), 1.77 (dd, *J* = 21.9, 8.9 Hz, 4H), 1.43-1.17 (m, 3H), 0.90 (s, 9H). ^{13}C NMR (75 MHz, CD_3OD): δ 168.9, 165.2, 142.1, 138.6, 120.7, 114.9, 65.0, 51.1, 33.5, 27.9, 26.8, 22.5. HRMS m/z calculated for $C_{20}H_{32}N_3O_2$: 346.2489 $[M+H]^+$; found, 346.2490.

5-(4-((1s,4s)-4-phenylcyclohexyl)piperazine-1-carbonyl)pyridin-2(1H)-one (**20**). **20** synthesized by reacting the previously obtained free-piperazine compound (177 mg, 1.1 eq.) and 4-phenyl-cyclohexanone (87 mg, 1 eq.) as in **19**, and was purified by RP-UHPLC (from 75%A25%D to 60%A40%D in 30 min; RT_{7min}: 1.62 min) to be afforded as a white powder (96 mg, 23 %). ^1H NMR (300 MHz, D_2O): δ 7.84 (d, *J* = 2.1 Hz, 1H), 7.75 (dd, *J* = 9.4, 2.5 Hz, 1H), 7.47-7.40 (m, 4H), 7.30 (dt, *J* = 8.4, 2.1 Hz, 1H), 6.69 (d, *J* = 9.5 Hz, 1H), 4.36 (s, 2H), 3.68-3.46 (m, 5H), 3.18 (dt, *J* = 12.3, 3.3 Hz, 2H), 3.03 (s, 1H), 2.25-2.15 (m, 2H), 2.01-1.81 (m, 6H). ^{13}C NMR (75 MHz, D_2O): δ 168.5, 164.6, 144.1, 141.6, 136.9, 128.7, 127.3, 126.2, 119.4, 117.5, 114.4, 65.2, 48.6, 37.1, 27.3, 23.2. HRMS m/z calculated for $C_{22}H_{28}N_3O_2$: 366.2176 $[M+H]^+$; found, 366.2184.

1-((1s,4s)-4-phenylcyclohexyl)-4-(pyridin-3-ylmethyl)piperazine (**24**). General procedure C: In a round-bottom flask containing a solution of 1-(4-phenylcyclohexyl)piperazine[1] (169 mg, 1.1 eq.), in dry DCE (5 mL) was added Et_3N (280 µL, 4 eq.). After 15 min, nicotinaldehyde (**21**, 47 µL, 1 eq.), and $NaBH(OAc)_3$ (144 mg, 1.3 eq.) were added, and the mixture was stirred for 24 h, at r.t. The reaction mixture was evaporated under vacuum and was purified by RP-UHPLC (from 75%A25%D to 60%A40%D in 20 min; RT_{7min}: 1.56 min) to be afforded as a white powder (193 mg, 68 %). ^1H NMR (300 MHz, C_6D_6): δ 8.73 (d, *J* = 1.6 Hz, 1H), 8.53 (dd, *J* = 4.7, 1.6 Hz, 1H), 7.38 (dt, *J* = 7.7, 1.9 Hz, 1H), 7.29-7.36 (m, 4H), 7.10 (dt, *J* = 6.9, 1.7 Hz, 1H), 6.79 (dd, *J* = 7.7, 4.7 Hz, 1H), 3.14 (s, 2H), 2.52 (tt, *J* = 11.1, 3.9 Hz, 1H), 2.28 (s, 8H), 2.09-2.06 (m, 1H), 2.02-1.83 (m, 4H), 1.55-1.49 (m, 2H), 1.34-1.24 (m, 2H). ^{13}C NMR (75 MHz, $CDCl_3$): δ 151.1, 149.1, 147.9, 136.1, 134.3, 128.7, 127.3, 126.2, 123.2, 60.4, 58.7, 53.9, 50.3, 44.3, 28.9, 28.7. HRMS m/z calculated for $C_{22}H_{30}N_3$: 336.2434 $[M+H]^+$; found, 336.2435.

1-((6-methoxypyridin-3-yl)methyl)-4-((1s,4s)-4-phenylcyclohexyl)piperazine (**25**). **25** was synthesized by reacting 1-(4-phenylcyclohexyl)piperazine[1] (169 mg, 1.1 eq.) and 6-methoxynicotinaldehyde (**22**, 69 mg,

1 eq.) following general procedure C, and was afforded as a white powder (94 mg, 43 %). ^{1}H NMR (300 MHz, D_2O): δ 8.25 (d, *J* = 2.2 Hz, 1H), 7.98 (dd, *J* = 8.8, 2.5 Hz, 1H), 7.44-7.38 (m, 4H), 7.29 (ddd, *J* = 8.5, 5.6, 3.0 Hz, 1H), 7.08 (d, *J* = 8.8 Hz, 1H), 4.38 (s, 2H), 3.99 (s, 3H), 3.58 (s, 8H), 2.99 (s, 1H), 2.16 (dd, *J* = 13.2, 10.9 Hz, 2H), 1.94 (ddd, *J* = 16.2, 15.7, 8.3 Hz, 6H). ^{13}C NMR (75 MHz, D_2O): δ 164.5, 147.8, 144.1, 143.7, 128.7, 127.2, 126.2, 118.6, 111.2, 65.3, 57.0, 55.0, 48.0, 46.7, 37.5, 27.2, 23.4. HRMS m/z calculated for $C_{23}H_{32}N_3O$: 366.2540 $[M+H]^+$; found, 366.2521.

1-((6-bromopyridin-3-yl)methyl)-4-((1s,4s)-4-phenylcyclohexyl)piperazine (**26**). **26** was synthesized by reacting 1-(4-phenylcyclohexyl)piperazine[1] (337 mg, 1.2 eq.) and 6-bromo-nicotinaldehyde (**23**, 186 mg, 1 eq.) following general procedure C, and was afforded as a white powder (294 mg, 60 %). ^{1}H NMR (300 MHz, C_6D_6): δ 8.19 (d, *J* = 1.4 Hz, 1H), 7.29-7.20 (m, 4H), 7.10 (dt, *J* = 7.0, 1.7 Hz, 1H), 7.03-6.96 (m, 2H), 2.91 (s, 2H), 2.53 (tt, *J* = 11.0, 3.8 Hz, 1H), 2.3 (s, 4H), 2.2 (2, 4H), 2.07 (t, *J* = 3.0 Hz, 1H), 2.02-1.84 (m, 4H), 1.56-1.51 (m, 2H), 1.37-1.24 (m, 2H). ^{13}C NMR (75 MHz, C_6D_6): δ 150.9, 147.8, 141.3, 138.9, 133.8, 128.7, 127.8, 127.3, 126.3, 59.3, 58.67, 53.8, 50.2, 44.3, 28.8, 28.7. HRMS m/z calculated for $C_{22}H_{29}BrN_3$: 414.1539 $[M+H]^+$; found, 414.1526.

5-((4-((1s,4s)-4-(m-tolyl)cyclohexyl)piperazin-1-yl)methyl)pyridin-2(1H)-one (**30**). In a round-bottom flask containing a solution of 4-(m-tolyl)cyclohexan-1-one (**27**, 378 mg, 1 eq.) and benzylpiperazine (416 µL, 1.2 eq.) in dry DCE (10 mL), was added AcOH (100 µL,). After 15 min, $NaBH(OAc)_3$ (593 mg, 1.4 eq.) was added and the mixture was stirred for 48 h at r.t. The solvent was removed under vacuum and the crude was purified by column chromatography (hexanes:EtOAc, 9:1 + 0.5 % Et_3N). The obtained intermediate was dissolved in Et_2O and was precipitated by the addition of a methanol solution of HCl (3.0 M). The collected solids were washed with Et_2O and hexanes and were used in the next step without further purification. The previously obtained compound was dissolved in a mixture of methanol (27 mL) and AcOH (106 µL, 2 eq.). To this stirring solution, Pd/C (30 mg, 0.3 eq.) was added and the reactional mixture was stirred for 16 h, at r.t., under H_2 atmosphere. The reaction mixture was filtered through a pad of Celite to remove the catalyst (washed with MeOH, 3 x 50 mL). The solvent was removed under vacuum and the compound was purified by RP-UHPLC (from 100%A0%D to 80%A20%D, in 20 min; RT_{5min}: 1.58 min). The desired compound was obtained as white powder (385 mg, 38 %). ^{1}H-NMR (300 MHz, D_2O, δ = ppm): δ 7.23 (dt, *J* = 18.3, 7.7 Hz, 3H), 7.09 (d, *J* = 7.4 Hz, 1H), 3.85-3.35 (m, 9H), 2.90 (d, *J* = 3.2 Hz, 1H), 2.30 (s, 3H), 2.10 (dd, *J* = 17.0, 6.9 Hz, 2H), 2.00-1.78 (m, 6H). ^{13}C-NMR (75 MHz, D_2O, δ = ppm): δ 144.2,138.8, 128.7, 127.8, 126.8, 124.1, 65.5, 45.9, 40.5, 37.4, 27.1, 23.3, 20.4. In a round-bottom flask containing a solution of the previously obtained compound (195 mg, 1.1 eq.) and **15** (45 mg, 1 eq.) in dry DCE (4 mL), was added Et_3N (223 µL, 1.4 eq.). After 15 min, $NaBH(OAc)_3$ (110 mg, 1.3 eq.) was added and the mixture was stirred for 24 h at r.t. The solvent was removed under vacuum and the crude was purified by RP-UHPLC (from 80%A20%D to 60%A40%D, in 20 min; RT_{5min}: 1.61 min). The desired compound was obtained as white powder (181 mg, 76 %). ^{1}H-NMR (300 MHz, CD_3CN:D_2O, 3:1, δ = ppm): δ 7.57 (d, *J* = 7.7 Hz, 1H), 7.16 (t, *J* = 7.5 Hz, 1H), 7.08 (d, *J* = 9.7 Hz, 1H), 6.99 (d, *J* = 7.3 Hz, 1H), 6.50 (d, *J* = 10.0 Hz, 1H), 4.04 (s, 2H), 3.50 (s, 12H), 3.36 (dd, *J* = 10.2, 5.0 Hz, 1H), 2.82-2.76 (m, 1H), 2.27 (s, 3H), 2.04-1.88 (m, 7H), 1.79-1.71 (m, 2H). ^{13}C-NMR (75 MHz, CD_3CN:D_2O, 3:1, δ = ppm): δ 164.5, 145.6, 144.9, 139.3, 139.1, 129.4, 128.8, 127.7, 125.1, 121.2, 118.8, 109.5, 65.5, 57.4, 48.7, 48.0, 39.9, 28.4, 25.0, 21.5. HRMS m/z calculated for $C_{23}H_{32}N_3O$: 366.2540 $[M+H]^+$; found, 366.2552.

5-((4-((1s,4s)-4-(3-(trifluoromethyl)phenyl)cyclohexyl)piperazin-1-yl)methyl)pyridin-2(1H)-one (**31**). In a round-bottom flask containing a solution of 4-(3-(trifluoromethyl)phenyl)cyclohexan-1-one (**28**, 488 mg, 1 eq.) and benzylpiperazine (416 µL, 1.2 eq.) in dry DCE (10 mL), was added AcOH (100 µL,). After 15 min, $NaBH(OAc)_3$ (593 mg, 1.4 eq.) was added and the mixture was stirred for 48 h at r.t. The solvent was removed under vacuum and the crude was purified by column chromatography (hexanes:EtOAc, 9:1 + 0.5 % Et_3N). The obtained intermediate was dissolved in Et_2O and was precipitated by the addition of a methanol solution of HCl (3.0 M). The collected solids were washed with Et_2O and hexanes and were used in the next step without further purification. The previously obtained compound was dissolved in a mixture of methanol (16 mL) and AcOH (74 µL, 2 eq.). To this stirring solution, Pd/C (21 mg, 0.3 eq.) was added and the reactional mixture was stirred for 16 h, at r.t., under H_2 atmosphere. The reaction mixture was filtered through a pad of Celite to remove the catalyst (washed with MeOH, 3 x 50 mL). The solvent was removed under vacuum and the compound was purified by RP-UHPLC (from 95%A05%D to 70%A30%D, in 20 min; RT_{5min}: 1.66 min). The desired compound was obtained as white powder (311 mg, 28 %). ^{1}H NMR (300 MHz, D_2O): δ 7.71 (s, 1H), 7.65-7.52 (m, 3H), 3.89-3.54 (m, 9H), 3.06 (t, *J* = 5.5, 2.4 Hz,

1H), 2.21-2.10 (m, 2H), 2.06-1.84 (m, 6H). ^{13}C NMR (75 MHz, D_2O): δ 144.9, 131.0, 130.3, 129.9, 129.2, 123.8 (q, *J* = 3.8 Hz), 122.9 (q, *J* = 3.7 Hz), 117.5, 65.3, 46.0, 40.6, 37.5, 26.9, 23.2. ^{19}F NMR (376 MHz, D_2O): -62.3, -75.6. In a round-bottom flask containing a solution of the previously obtained compound (270 mg, 1.1 eq.) and **15** (56 mg, 1 eq.) in dry DCE (5 mL), was added Et_3N (280 µL, 1.4 eq.). After 15 min, $NaBH(OAc)_3$ (140 mg, 1.3 eq.) was added and the mixture was stirred for 24 h at r.t. The solvent was removed under vacuum and the crude was purified by RP-UHPLC (from 70%A30%D to 55%A45%D, in 20 min; RT_{5min}: 1.74 min) and was obtained as white powder (284 mg, 81 %). ^1H-NMR (300 MHz, CD_3CN:D_2O, 3:1, δ = ppm): δ 7.60-7.55 (m, 4H), 7.52-7.45 (m, 2H), 4.07 (s, 2H), 3.52 (s, 8H), 2.96-2.89 (m, 1H), 2.04 (ddd, *J* = 11.7, 10.4, 5.2 Hz, 2H), 1.96-1.90 (m, 5H), 1.84-1.75 (m, 2H).^{13}C-NMR (75 MHz, CD_3CN:D_2O, 3:1, δ = ppm): δ 164.3, 147.0, 144.8, 139.5, 132.1, 131.2, 130.7, 130.3, 127.4, 125.0, 124.8 (q, *J* = 3.8 Hz), 123.9 (q, *J* = 3.8 Hz),121.3, 119.5, 118.7, 109.1, 65.2, 57.4, 48.6, 48.1, 40.1, 28.1, 25.1. ^{19}F NMR (376 MHz, CD_3CN:D_2O, 3:1, δ = ppm): δ -62.9, -76.0. HRMS m/z calculated for $C_{23}H_{29}F_3N_3O$: 420.2257 $[M+H]^+$; found, 420.2265.

5-((4-((1s,4s)-4-(2-(trifluoromethyl)phenyl)cyclohexyl)piperazin-1-yl)methyl)pyridin-2(1H)-one (**32**). In a round-bottom flask containing a solution of 4-(2-(trifluoromethyl)phenyl)cyclohexan-1-one (**29**, 365 mg, 1 eq.) and benzylpiperazine (312 µL, 1.2 eq.) in dry DCE (7.5 mL), was added AcOH (100 µL,). After 15 min, $NaBH(OAc)_3$ (445 mg, 1.4 eq.) was added and the mixture was stirred for 48 h at r.t. The solvent was removed under vacuum and the crude was purified by column chromatography (hexanes:EtOAc, 9:1 + 0.5 % Et_3N). The obtained intermediate was dissolved in Et_2O and was precipitated by the addition of a methanol solution of HCl (3.0 M). The collected solids were washed with Et_2O and hexanes and were used in the next step without further purification. The previously obtained compound was dissolved in a mixture of methanol (10 mL) and AcOH (46 µL, 2 eq.). To this stirring solution, Pd/C (13 mg, 0.3 eq.) was added and the reactional mixture was stirred for 16 h, at r.t., under H_2 atmosphere. The reaction mixture was filtered through a pad of Celite to remove the catalyst (washed with MeOH, 3 x 50 mL). The solvent was removed under vacuum and the compound was purified by RP-UHPLC (from 95%A05%D to 70%A30%D, in 20 min; RT_{5min}: 1.69 min). The desired compound was obtained as white powder (215 mg, 27 %). ^1H NMR (300 MHz, D_2O): δ 7.74-7.60 (m, 3H), 7.40 (t, *J* = 7.4 Hz, 1H), 4.07-3.50 (m, 9H), 3.19-3.12 (m, 1H), 2.38 (d, *J* = 16.5 Hz, 1H), 2.03 (tt, *J* = 16.5, 3.8 Hz, 1H), 1.87-1.68 (m, 4H). ^{13}C NMR (75 MHz, D_2O): δ 144.5, 132.5, 128.0, 127.4, 127.0, 126.6, 126.4, 126.0 (q, *J* = 6.1 Hz), 63.9, 47.0, 40.2, 38.0, 26.9, 25.0. ^{19}F NMR (376 MHz, D_2O): -58.8, -75.6. In a round-bottom flask containing a solution of the previously obtained compound (215 mg, 1.1 eq.) and **15** (45 mg, 1 eq.) in dry DCE (4 mL), was added Et_3N (223 µL, 1.4 eq.). After 15 min, $NaBH(OAc)_3$ (110 mg, 1.3 eq.) was added and the mixture was stirred for 24 h at r.t. The solvent was removed under vacuum and the crude was purified by RP-UHPLC (from 70%A30%D to 55%A45%D, in 20 min; RT_{5min}: 1.75 min) and was obtained as white powder (186 mg, 72 %). ^1H-NMR (300 MHz, CD_3CN:D_2O, 3:1, δ = ppm): δ 7.80 (d, *J* = 7.9 Hz, 1H), 7.64 (d, *J* = 9.2 Hz, 3H), 7.50 (t, *J* = 7.5 Hz, 1H), 7.34 (t, *J* = 7.6 Hz, 1H), 6.56 (d, *J* = 9.8 Hz, 1H), 4.10 (s, 2H), 3.69 (s, 8H), 3.37 (s, 1H), 3.04 (t, *J* = 8.5 Hz, 1H), 2.34 (t, *J* = 13.4 Hz, 2H), 2.00-1.82 (m, 5H), 1.67 (d, *J* = 10.1 Hz, 2H). ^{13}C-NMR (75 MHz, CD_3CN:D_2O, 3:1, δ = ppm): δ 163.8, 161.6 (q, *J* = 35.7 Hz), 145.4, 139.6, 133.4, 129.8, 128.2, 127.8, 127.7, 127.5, 126.5 (q, *J* = 6.0 Hz), 124.1, 120.8, 119.4, 118.3, 115.6, 109.7, 64.2, 57.0, 49.0, 48.1, 39.8, 28.0, 26.6. ^{19}F NMR (376 MHz, CD_3CN:D_2O, 3:1, δ = ppm): δ -59.3, -76.1. HRMS m/z calculated for $C_{23}H_{29}F_3N_3O$: 420.2257 $[M+H]^+$; found, 420.2270.

4-hydroxy-4-(3-(trifluoromethyl)phenyl)cyclohexan-1-one (**37**). A dry three-neck round-bottom flask equipped with a condenser was charged with Mg (349 mg, 1.4 eq.) and dry THF (1.0 mL) under Argon atmosphere. To this suspension, a solution of 1-bromo-3-trifluormethyl-benzene (**33**, 1.7 mL, 1.2 eq.) in dry THF (0.4 mL) was added dropwise using an addition funnel. The reaction mixture was stirred under reflux and Argon atmosphere for 30 min. The obtained Grignard-reagent was used in situ, without purification. After cooling the previous mixture to r.t., a solution of 1,4-dioxaspiro[4.5]decan-8-one (1.6 g, 1 eq.) in dry THF (1 mL) was added dropwise and the reaction was stirred for another 30 min, under reflux and Argon atmosphere. The reaction was quenched by addition of aqueous saturated solution of NH_4Cl (6 mL) and was extracted with Et_2O (3 x 50 mL). The combined organic phase was dried over $MgSO_4$ and evaporated under vacuum. The obtained residue column chromatographed (hexanes:EtOAc, 95:05 to 75:25) to yield the intermediate **35** used in the next step without further purification. In a round-bottom flask containing a solution of **35** (2.7 g, 1 eq.) was dissolved in a mixture of acetone and H_2O (1:1, 160 mL). To this solution, PPTS (4.4 g, 2.0 eq.) was added and the mixture was stirred at 60 °C for 6 h. The organic solvent was removed under vacuum and the resulting aqueous phase was extracted with EtOAc (3 x 100 mL). The collected organic phase was dried over Na_2SO_4 and evaporated. The crude was column

chromatographed (hexanes:EtOAc, 9:1) to yield the desired ketone as a white powder (1.9 g, 71 %). ^{1}H NMR (300 MHz, C_6D_6, δ = ppm): δ 7.62 (s, 1H), 7.29 (d, *J* = 7.7 Hz, 1H), 7.07 (d, *J* = 8.0 Hz, 1H), 6.93 (t, *J* = 7.8 Hz, 1H), 2.49 (td, *J* = 14.0, 6.6 Hz, 2H), 2.09 (dd, *J* = 4.7, 2.1 Hz, 1H), 2.05 (dd, *J* = 4.7, 2.1 Hz, 1H), 1.51 (td, *J* = 13.7, 4.7 Hz, 2H), 1.42-1.33 (m, 2H). ^{13}C NMR (75 MHz, C_6D_6, δ = ppm): δ 208.2, 149.2, 130.8 (q, *J* = 31.9 Hz), 129.0, 128.3, 124.1 (q, *J* = 3.7 Hz), 121.7 (q, *J* = 3.8 Hz), 71.6, 38.1, 37.1. ^{19}F NMR (376 MHz, C_6D_6, δ = ppm): δ -62.1.

4-hydroxy-4-(2-(trifluoromethyl)phenyl)cyclohexan-1-one (**38**). A dry three-neck round-bottom flask equipped with a condenser was charged with Mg (342 mg, 1.4 eq.) and dry THF (1.0 mL) under Argon atmosphere. To this suspension, a solution of 1-bromo-2-trifluormethyl-benzene (**34**, 1.7 mL, 1.2 eq.) in dry THF (0.4 mL) was added dropwise using an addition funnel. The reaction mixture was stirred under reflux and Argon atmosphere for 30 min. The obtained Grignard-reagent was used in situ, without purification. After cooling the previous mixture to r.t., a solution of 1,4-dioxaspiro[4.5]decan-8-one (1.6 g, 1 eq.) in dry THF (1 mL) was added dropwise and the reaction was stirred for another 30 min, under reflux and Argon atmosphere. The reaction was quenched by addition of aqueous saturated solution of NH_4Cl (6 mL) and was extracted with Et_2O (3 x 50 mL). The combined organic phase was dried over $MgSO_4$ and evaporated under vacuum. The obtained residue column chromatographed (hexanes:EtOAc, 95:05 to 75:25) to yield the intermediate **36** used in the next step without further purification. In a round-bottom flask containing a solution of **36** (471 mg, 1 eq.) was dissolved in a mixture of acetone and H_2O (1:1, 20 mL). To this solution, PPTS (1 g, 2.0 eq.) was added and the mixture was stirred at 60 °C for 6 h. The organic solvent was removed under vacuum and the resulting aqueous phase was extracted with EtOAc (3 x 50 mL). The collected organic phase was dried over Na_2SO_4 and evaporated. The crude was column chromatographed (hexanes:EtOAc, 9:1) to yield the desired ketone as a white powder (292 mg, 38 %). ^{1}H NMR (300 MHz, C_6D_6, δ = ppm): δ 7.61 (dd, *J* = 7.9, 0.9 Hz, 1H), 6.96 (td, *J* = 7.9, 1.1 Hz, 1H), 6.82 (dd, *J* = 16.5, 8.0 Hz, 2H), 2.61-2.50 (m, 2H), 2.09 (d, *J* = 2.1 Hz, 1H), 2.05 (dd, *J* = 4.2, 2.1 Hz, 1H), 1.77-1.62 (m, 4H), 1.52 (s, 1H). ^{13}C NMR (75 MHz, C_6D_6, δ = ppm): δ 208.6, 147.3, 131.5, 128.5, 127.6, 127.4, 123.8, 73.0, 38.6, 38.6, 37.1. ^{19}F NMR (376 MHz, C_6D_6, δ = ppm): δ -53.4.

5-((4-((1r,4r)-4-hydroxy-4-(3-(trifluoromethyl)phenyl)cyclohexyl)piperazin-1-yl)methyl)pyridin-2(1H)-one (**39,** 3OG). In a round-bottom flask containing a solution of **37** (1.9 g, 1 eq.) and benzylpiperazine (1.5 mL, 1.2 eq.) in dry DCE (72 mL), was added AcOH (400 µL,). After 15 min, $NaBH(OAc)_3$ (2.1 g, 1.4 eq.) was added and the mixture was stirred for 24 h at r.t. The solvent was removed under vacuum and the crude was purified by column chromatography (hexanes:EtOAc, 7:3 + 0.5 % Et_3N). The obtained intermediate was dissolved in Et_2O and was precipitated by the addition of a methanol solution of HCl (3.0 M). The collected solids were washed with Et_2O and hexanes and were used in the next step without further purification. The previously obtained compound was dissolved in a mixture of methanol (65 mL) and AcOH (210 µL, 2 eq.). To this stirring solution, Pd/C (55 mg, 0.3 eq.) was added and the reactional mixture was stirred for 16 h, at r.t., under H_2 atmosphere. The reaction mixture was filtered through a pad of Celite to remove the catalyst (washed with MeOH, 3 x 100 mL). The desired compound was obtained as white powder (540 mg, 18 %). ^{1}H NMR (300 MHz, D_2O): δ 7.90 (s, 1H), 7.84 (d, *J* = 7.8 Hz, 1H), 7.72 (d, *J* = 7.7 Hz, 1H), 7.64 (t, *J* = 7.8 Hz, 1H), 3.59 (s, 9H), 2.69 (d, *J* = 14.0 Hz, 2H), 2.24 (d, *J* = 10.2 Hz, 2H), 1.95-1.85 (m, 2H), 1.56 (q, *J* = 10.8 Hz, 2H). ^{13}C NMR (75 MHz, D_2O): δ 162.9 (q, *J* = 35.3 Hz), 143.0, 130.7 (t, *J* = 31.9 Hz), 129.8, 129.7, 129.6, 124.9 (q, *J* = 3.6 Hz), 122.9 (q, *J* = 3.8 Hz), 116.4 (q, *J* = 301.5 Hz), 71.8, 64.7, 45.9, 40.7, 34.0, 23.3. ^{19}F NMR (376 MHz, D_2O, δ = ppm): -62.3, -75.6. In a round-bottom flask containing a solution of the previously obtained compound (540 mg, 1.1 eq.) and **15** (198 mg, 1.2 eq.) in MeOH (15 mL) was added $NaBH_3CN$ (102 mg,1.2 eq the mixture was stirred for 24 h at r.t. The solvent was removed under vacuum and the crude was purified by column chromatography (DCM:MeOH, 95:5 + 0.5 % Et_3N to 92:8 + 0.5 % NEt_3). The product was dissolved in Et_2O and was precipitated by the addition of a methanol solution of HCl (3.0 M) to obtain the desired product as white powder (335 mg, 48 %). ^{1}H-NMR (400 MHz, D_2O, δ = ppm): δ 7.90 (s, 1H), 7.85 (d, *J* = 7.9 Hz, 1H), 7.76-7.73 (m, 2H), 7.65 (t, *J* = 7.8 Hz, 1H), 6.70 (dd, *J* = 10.4, 2.2 Hz, 1H), 4.26 (s, 2H), 3.60-3.58 (m, 9H), 2.69 (d, *J* = 14.1 Hz, 1H), 2.26-2.23 (m, 2H), 1.94-1.87 (m, 2H), 1.56 (q, *J* = 10.7 Hz, 2H). ^{13}C-NMR (100 MHz, D_2O, δ = ppm): δ 164.5, 162.9 (q, *J* = 35.4 Hz), 144.8, 143.0, 138.5, 130.5 (q, *J* = 32.0 Hz), 129.9, 129.7, 128.2, 125.5, 124.9 (q, *J* = 3.8 Hz), 122.9 (q, *J* = 3.7 Hz), 122.8, 120.0, 116.3 (q, *J* = 291.8 Hz), 109.2, 71.8, 64.5, 56.7, 48.0, 46.4, 34.0, 23.5. ^{19}F NMR (376 MHz, D_2O, δ = ppm): δ -62.3, -75.6. HRMS m/z calculated for $C_{23}H_{28}F_3N_3O_2$: 436.2206 $[M+H]^+$; found, 436.2207.

5-((4-((1r,4r)-4-hydroxy-4-(2-(trifluoromethyl)phenyl)cyclohexyl)piperazin-1-yl)methyl)pyridin-2(1H)-one (**40**). In a round-bottom flask containing a solution of **38** (292 mg, 1 eq.) and benzylpiperazine (235 µL, 1.2 eq.) in dry DCE (11 mL), was added AcOH (100 µL). After 15 min, $NaBH(OAc)_3$ (335 mg, 1.4 eq.) was added and the mixture was stirred for 48 h at r.t. The solvent was removed under vacuum and the crude was purified by column chromatography (hexanes:EtOAc, 7:3 + 0.5 % Et_3N). The obtained intermediate was dissolved in Et_2O and was precipitated by the addition of a methanol solution of HCl (3.0 M). The collected solids were washed with Et_2O and hexanes and were used in the next step without further purification. The previously obtained compound was dissolved in a mixture of methanol (12 mL) and AcOH (38 µL, 2 eq.). To this stirring solution, Pd/C (11 mg, 0.3 eq.) was added and the reactional mixture was stirred for 16 h, at r.t., under H_2 atmosphere. The reaction mixture was filtered through a pad of Celite to remove the catalyst (washed with MeOH, 3 x 50 mL). The solvent was removed and the desired compound was obtained as white powder (113 mg, 19 %). ^{1}H NMR (300 MHz, D_2O): δ 7.90 (d, *J* = 7.8 Hz, 1H), 7.75 (d, *J* = 7.8 Hz, 1H), 7.65 (t, *J* = 7.3 Hz, 1H), 7.51 (t, *J* = 7.2 Hz, 1H), 3.63 (s, 8H), 3.52 (s, 1H), 2.45 (t, *J* = 9.5 Hz, 2H), 2.26 (d, *J* = 10.0 Hz, 2H), 1.97 (t, *J* = 17.4 Hz, 4H). ^{13}C NMR (75 MHz, D_2O): δ 144.3, 132.1, 128.8 (q, *J* = 7.2 Hz), 128.3, 127.9, 127.6, 127.2, 126.7, 123.0, 72.9, 63.6, 46.5, 40.9, 34.0, 22.4. ^{19}F NMR (376 MHz, D_2O, δ = ppm): -53.8. In a round-bottom flask containing a solution of the previously obtained compound (113 mg, 1.1 eq.) and **15** (28 mg, 1 eq.) in dry DCE (2.5 mL), was added Et_3N (128 µL, 4 eq.). After 15 min, $NaBH(OAc)_3$ (63 mg, 1.3 eq.) was added and the mixture was stirred for 24 h at r.t. The solvent was removed under vacuum and the crude was purified by RP-UHPLC (from 80%A20%D to 70%A30%D, in 20 min; RT_{5min}: 1.49 min) and was obtained as white powder (91 mg, 60 %). ^{1}H-NMR (400 MHz, D_2O, δ = ppm): δ 7.90 (dd, *J* = 7.9, 0.8 Hz, 1H), 7.79-7.76 (m, 2H), 7.71 (d, *J* = 7.9 Hz, 1H), 7.66-7.62 (m, 1H), 7.51 (t, *J* = 7.6 Hz, 1H), 6.71 (d, *J* = 10.2, 4.5 Hz, 1H), 4.33 (s, 2H), 3.67-3.62 (m, 9H), 2.41 (dd, *J* = 18.1, 8.9 Hz, 2H), 2.31 (dd, *J* = 13.7, 7.3 Hz, 2H), 2.00-1.93 (m, 4H). ^{13}C-NMR (100 MHz, D_2O, δ = ppm): δ 164.5, 162.9 (q, *J* = 35.4 Hz), 144.8, 144.2, 138.6, 132.1, 128.9 (q, *J* = 7.4 Hz), 128.2, 128.0, 127.5 (q, *J* = 30.9 Hz), 126.2, 123.5, 120.1, 116.4 (q, *J* = 291.9 Hz), 109.0, 72.7, 63.9, 56.7, 47.8, 47.0, 33.9, 22.3. ^{19}F NMR (376 MHz, D_2O, δ = ppm): δ -53.9, -75.6. HRMS m/z calculated for $C_{23}H_{28}F_3N_3O_2$: 436.2206 $[M+H]^+$; found, 436.2209.

(E)-N'-(4-hydroxy-3-methoxybenzylidene)hexane-1-sulfonohydrazide (**41**). In a round-bottom flask containing an ice-cold solution of hydrazine hydrate (9.4 mL, 10 eq.) in THF (10 mL) was added dropwise a solution of hexane-1-sulfonyl chloride (1.6 mL, 1 eq.) in THF (10 mL). The reaction mixture was stirred for 1 h, at 0 °C, then, it was allowed to warm to r.t. and was stirred for an additional 1 h. The reaction mixture was extracted with EtOAc (5 X 5 mL) and was dried over Na_2SO_4. The resulting solution was evaporated and was solubilized in a minimal amount of chloroform. To this solution, hexane was slowly added to precipitate the desired compound as a white solid (1.5 g, 82 %). Then, in a round-bottom flask containing a solution of the previously obtained compound (270 mg, 1 eq.) in MeOH (30 mL), was added dropwise a solution of **4** (228 mg, 1 eq.). The reaction mixture was stirred for 5 h, under reflux. Under completion (TLC), the reaction mixture was evaporated and column chromatographed (hexanes:EtOAc, 50:50) to yield the desired compound as an yellow sticky oil (419 mg, 89 %). ^{1}H NMR (300 MHz, $CDCl_3$): δ 7.95 (s, 1H), 7.76 (s, 1H), 7.29 (d, *J* = 1.7 Hz, 1H), 7.02 (dd, J^1 = 1.7 Hz, J^2 = 8.4 Hz, 1H), 6.89 (d, *J* = 8.1 Hz, 1H), 5.90 (s, 1H), 3.92 (s, 3H), 3.28 (t, *J* = 8.0 Hz, 2H), 1.85 (p, *J* = 7.7 Hz, 2H), 1.43 (p, *J* = 3.6 Hz, 2H), 1.30-1.25 (m, 4H), 0.85 (t, *J* = 6.9 Hz, 3H).^{13}C NMR (75 MHz, $CDCl_3$): δ 148.0, 146.8, 125.5, 122.8, 114.0, 107.6, 55.9, 50.9, 31.0, 27.7, 22.9, 22.0, 13.7. HRMS m/z calculated for $C_{14}H_{23}N_2O_4S$: 315.1373 $[M+H]^+$; found, 315.1366.

(E)-N'-(4-hydroxy-3-methoxybenzylidene)octane-1-sulfonohydrazide (**42**). **42** was synthesized from hydrazine hydrate (9.4 mL, 10 eq.) and octane-1-sulfonyl chloride (2 mL, 1 eq.) following the same procedures for compound **41** and was obtained as an yellow sticky oil (478 mg, 56 %). ^{1}H NMR (300 MHz, $CDCl_3$): δ 8.00 (s, 1H), 7.77 (s, 1H), 7.29 (d, *J* = 1.5 Hz, 1H), 7.03 (dd, J^1 = 1.8 Hz, J^2 = 8.1 Hz, 1H), 6.90 (d, *J* = 8.4 Hz, 1H), 5.91 (s, 1H), 3.93 (s, 3H), 3.28 (t, *J* = 8.0 Hz, 2H), 1.86 (p, *J* = 7.7 Hz, 2H), 1.45-1.38 (m, 2H), 1,27-1.24 (m, 8H), 0.85 (t, *J* = 6.8 Hz, 3H).^{13}C NMR (75 MHz, $CDCl_3$): δ 148.4, 148.2, 147.0, 125.8, 123.0, 114.3, 107.9, 56.1, 51.1, 31.7, 29.0, 28.9, 28.2, 23.1, 22.5, 14.0. HRMS m/z calculated for $C_{16}H_{27}N_2O_4S$: 343.1686 $[M+H]^+$; found, 343.1680.

(E)-N'-(4-hydroxy-3-methoxybenzylidene)heptanehydrazide (**43**). In a round-bottom flask containing an ice-cold solution of hydrazine hydrate (1 mL, 10 eq.) in DCM (6 mL), was added dropwise a solution of heptanoyl chloride (310 µL, 1 eq.) in DCM (6 mL). The reaction mixture was stirred for 1 h, at 0 °C, then, it was allowed to warm to r.t. and was stirred for additional 1 h. The reaction mixture was quenched by the

addition of H_2O (10 mL). The solution was transferred to a funnel and an aqueous solution of HCl 5% (5 mL) was added. The mixture was extracted and the aqueous phase was re-extracted with DCM (2 X 15 mL). The organic phase was collected, dried over $MgSO_4$ and evaporated. The resulting residue was solubilized in a minimal amount of DCM and was precipitated as a white solid (144 mg, 50 %) by the addition of hexane. The compound was used in the next step without further purification. This intermediate (144 mg, 1 eq.) was solubilized in EtOH (5 mL) and was added dropwise to a solution of **4** (152 mg, 1 eq.) in EtOH (5 mL), followed by AcOH (2 drops), and the reaction mixture was stirred at r.t., for 2 h. The reaction was quenched by the addition of cold H_2O until precipitation. The solids were filtered off and dried under vacuum. The crude was then, column chromatographed (hexanes:EtOAc, 1:2) to afford the desired compound as a white solid (137 mg, 25 %). *Note that N-acyl-hydrazones are known to generate a mixture of interconversible rotamers of the amide bound (sym/antiperiplanar) yielding duplicated NMR peaks.*[2,3] ^{1}H NMR (300 MHz, $CDCl_3$): δ 9.84 (s, 0.3H), 8.71 (s, 0.5H), 7.64 (s, 1H), 7.42 (s, 1H), 7.07 (dd, *J* = 12.3, 9.1 Hz, 1H), 6.93 (d, *J* = 7.0 Hz, 1H), 3.97 (d, *J* = 5.7 Hz, 3H), 2.74 (t, *J* = 7.3 Hz, 1H), 2.26 (t, *J* = 7.7 Hz, 1H), 1.76-1.67 (m, 2H), 1.33 (brs, 7H), 0.89 (s, 3H).^{13}C NMR (75 MHz, $CDCl_3$): δ 175.8, 148.0, 147.1, 143.2, 127.6, 122.4, 114.7, 108.0, 33.0, 31.8, 29.3, 24.9, 22.7, 14.2. HRMS m/z calculated for $C_{15}H_{23}N_2O_3$: 279.1703 [M+H]$^+$; found, 279.1710.

(E)-N'-(4-hydroxy-3-methoxybenzylidene)nonanehydrazide (**44**). **44** was synthesized from hydrazine hydrate (0.68 µL, 10 eq.) and nonanoyl chloride (252 µL, 1 eq.) following the same procedures for compound **43** and was obtained as white solid (129 mg, 31 %). ^{1}H NMR (300 MHz, DMSO-d_6): δ 11.10 (s, 0.5H), 11.00 (s, 0.5H), 9.60 (s, 0.1H), 9.42 (d, *J* = 6.1 Hz, 1H), 8.03 (s, 0.5H), 7.85 (s, 0.5H), 7.22 (d, *J* = 12.7 Hz, 1H), 7.03 (d, *J* = 8.1 Hz, 1H), 6.80 (dd, *J* = 8.1, 1.3 Hz, 1H), 3.80 (d, *J* = 2.9 Hz, 3H), 2.58 (t, *J* = 7.3 Hz, 1H), 2.16 (t, *J* = 7.3 Hz, 1H), 1.57 (dd, *J* = 13.4, 6.6 Hz, 2H), 1.25 (s, 11H), 0.86 – 0.81 (m, 2H). ^{13}C NMR (75 MHz, $CDCl_3$): δ 176.3, 147.9, 147.1, 143.6, 126.6, 122.3, 114.7, 108.1, 56.1, 32.9, 32.0, 29.6, 29.5, 29.3, 25.0, 22.8, 14.2. HRMS m/z calculated for $C_{17}H_{27}N_2O_3$: 307.2016 [M+H]$^+$; found, 307.2025.

(E)-N-(2-(2-(4-hydroxy-3-methoxybenzylidene)hydrazineyl)-2-oxoethyl)hexane-1-sulfonamide (**45**). In a round-bottom flask containing a solution of methyl glycinate hydrochloride salt (226 mg, 1 eq.) in DCM (2.5 mL), was added Et_3N (753 µL, 3 eq.). The reaction mixture was stirred at 0 °C for 10 min, followed by the dropwise addition of hexane-1-sulfonyl chloride (300 µL, 1 eq.) in DCM (2.5 mL). The reaction mixture was allowed to warm up to r.t. and was stirred for an additional 6 h. Under completion (TLC), the reaction mixture was extracted with H_2O (3 X 10 mL) and the organic phase was collected and dried over $MgSO_4$. The solvent was removed under vacuum until a minimal amount of DCM was enough to solubilize all the solids. Then, cold hexane was slowly added to precipitate the desired compound as a white solid (192 mg, 45 %). Then, in a round-bottom flask containing a solution of the previously obtained sulfonamide (177 mg, 1 eq.) in MeOH (185 µL), was added hydrazine hydrate (185 µL). The reaction mixture was stirred at r.t. for 3 h. Under completion (TLC), the solvents were removed under vacuum and the residue was solubilized in a minimal amount of EtOH. Hexane was added to the previous solution until precipitation of the desired compound as a white solid (131 mg, 75 %). Finally, in a round-bottom flask containing a solution of the obtained hydrazide-sulfonamide (47 mg, 1 eq.) in EtOH (1 mL), was added **4** (30 mg, 1 eq.) and AcOH (1 drop) in EtOH (1 mL). The reaction mixture was stirred at r.t. for 24 h. Under completion (TLC), the solvents were removed under vacuum and the residue was column chromatographed (hexanes:EtOAc, 75:25) to afford the desired compound as a white solid (65 mg, 87 %). ^{1}H NMR (300 MHz, $CDCl_3$): δ 9.13 (s, 1H), 7.69 (s, 1H), 7.24 (s, 1H), 7.08 (d, *J* = 8.4 Hz, 1H), 6.94 (d, *J* = 8.1 Hz, 1H), 5.93 (s, 1H), 5.19 (t, *J* = 5.1 Hz, 1H), 4.40 (d, *J* = 5.1 Hz, 2H), 3.95 (d, *J* = 13.8 Hz, 3H), 3.08 (t, *J* = 8.0 Hz, 2H), 1.87 (p, J= 7.8 Hz, 2H), 1.44-1.25 (m, 6H), 1.62 (s, 1H), 0.87 (t, *J* = 6.5 Hz, 3H). ^{13}C NMR (75 MHz, DMSO-d6): δ 169.8, 149.0, 148.7, 148.0, 147.9, 125.4, 125.3, 122.0, 121.2, 115.5, 115.4, 109.6, 109.1, 55.5, 52.4, 52.2, 43.3, 30.7, 27.2, 23.0, 21.8, 13.8. HRMS m/z calculated for $C_{16}H_{25}N_2O_5S$: 372.1588 [M+H]$^+$; found, 372.1582.

(E)-N-(2-(2-(4-hydroxy-3-methoxybenzylidene)hydrazineyl)-2-oxoethyl)octane-1-sulfonamide (**46**). In a round-bottom flask containing a solution of methyl glycinate hydrochloride salt (112 mg, 1 eq.) in DCM (1.2 mL), was added Et_3N (418 µL, 3 eq.). The reaction mixture was stirred at 0 °C for 10 min, followed by the dropwise addition of octane-1-sulfonyl chloride (196 µL, 1.1 eq.) in DCM (1.2 mL). The reaction mixture was allowed to warm up to r.t. and was stirred for an additional 6 h. Under completion (TLC), the reaction mixture was extracted with H_2O (3 X 10 mL) and the organic phase was collected and dried over $MgSO_4$. The solvent was removed under vacuum until a minimal amount of DCM was enough to solubilize all the solids. Then, cold hexane was slowly added to precipitate the desired compound as a white solid (122 mg,

52 %). Then, in a round-bottom flask containing a solution of the previously obtained sulfonamide (88 mg, 1 eq.) in MeOH (825 µL), was added hydrazine hydrate (825 µL). The reaction mixture was stirred at r.t. for 3 h. Under completion (TLC), the solvents were removed under vacuum and the residue was solubilized in a minimal amount of EtOH. Hexane was added to the previous solution until precipitation of the desired compound as a white solid (87 mg, 99 %). Finally, in a round-bottom flask containing a solution of the obtained hydrazide-sulfonamide (133 mg, 1 eq.) and **4** (76 mg, 1 eq.) and AcOH (1 drop) in EtOH (1 mL). The reaction mixture was stirred at r.t. for 24 h. Under completion (TLC), the solvents were removed under vacuum and the residue was column chromatographed (hexanes:EtOAc, 75:25) to afford the desired compound as a white solid (178 mg, 89 %). ^{1}H NMR (300 MHz, $CDCl_3$): δ 9.85 (s, 0.1H), 9.69 (s, 1H), 8.03 (s, 0.1H), 7.72 (s, 1H), 7.37 (s, 0.1H), 7.22 (s, 1H), 7.09 (d, *J* = 8.1 Hz, 1H), 7.01 (d, *J* = 8.1 Hz, 0.1H), 6.93 (d, *J* = 8.1 Hz, 1H), 6.85 (d, *J* = 8.0 Hz, 0.1H), 5.98 (s, 1H), 5.79 (t, *J* = 6.1 Hz, 0.1H), 5.34 (t, *J* = 5.1 Hz, 1H), 4.38 (d , *J* = 5.1 Hz, 2H), 3.95 (s, 3H), 3.85 (s, 0.4H), 3.07 (t, *J* = 7.78 Hz, 2H), 1.85 (p, *J* = 7.5 Hz, 2H), 1.40-1.26 (m, 10H), 0.86 (t, *J* = 6.0 Hz, 3H).^{13}C NMR (75 MHz, $CDCl_3$): δ 170.3, 148.5, 147.1, 146.3, 125.5, 123.0, 114.5, 108.0, 56.2, 53.2, 44.3, 31.7, 29.1, 29.0, 28.4, 23.6, 22.6, 14.0.HRMS m/z calculated for $C_{18}H_{30}N_2O_5S$: 400.1901 $[M+H]^+$; found, 400.1891.

2. Copy of ^{1}H and ^{13}C NMR spectra

4-((tert-butyldimethylsilyl)oxy)-3-methoxybenzaldehyde (**5**).

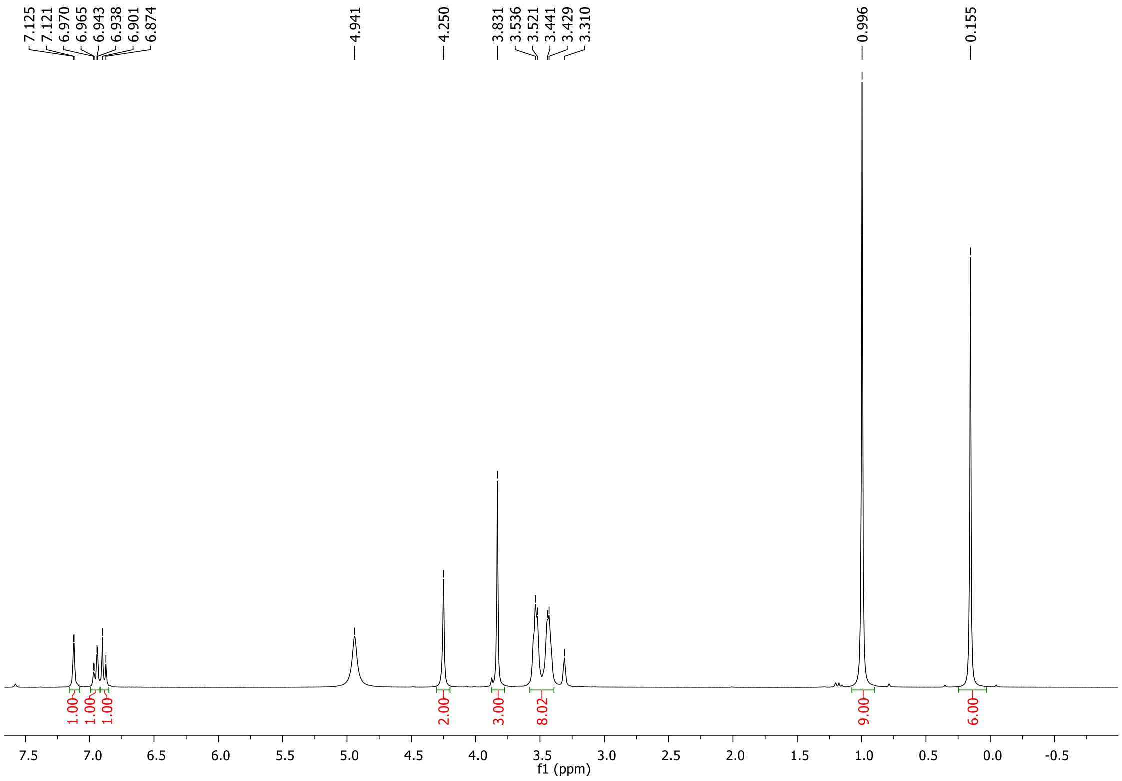

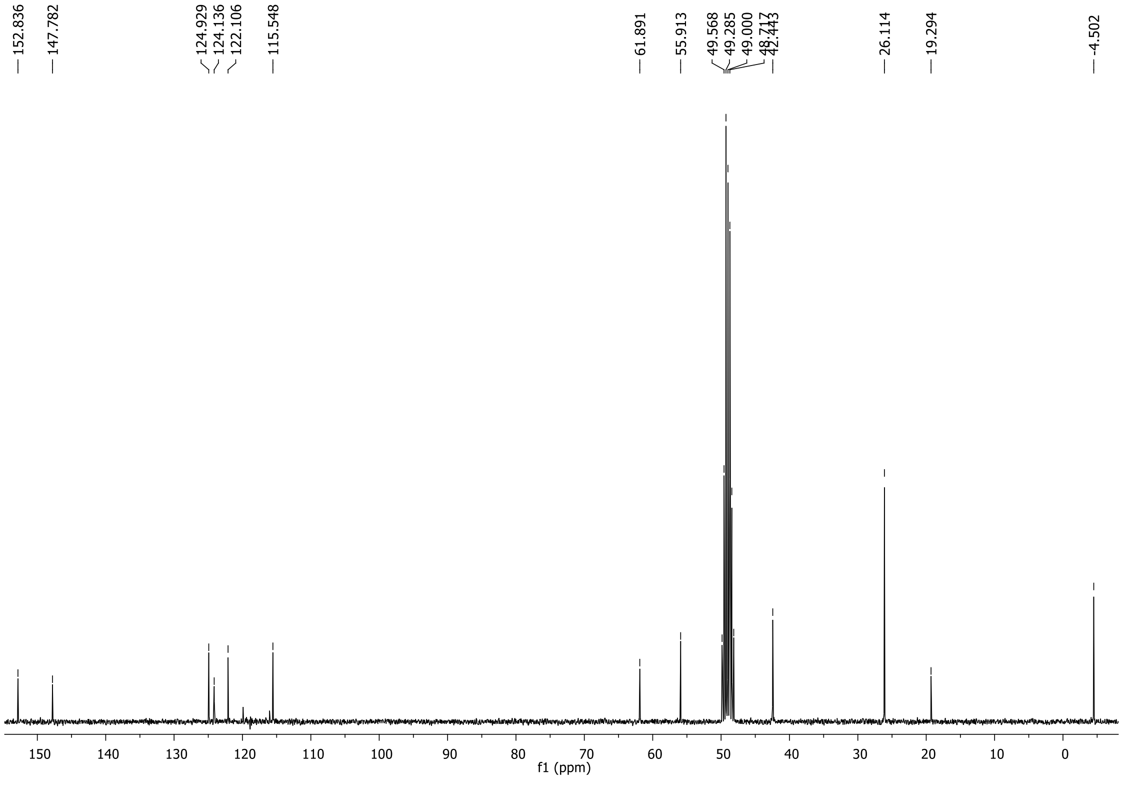

4-((4-cyclohexylpiperazin-1-yl)methyl)-2-methoxyphenol (**6**).

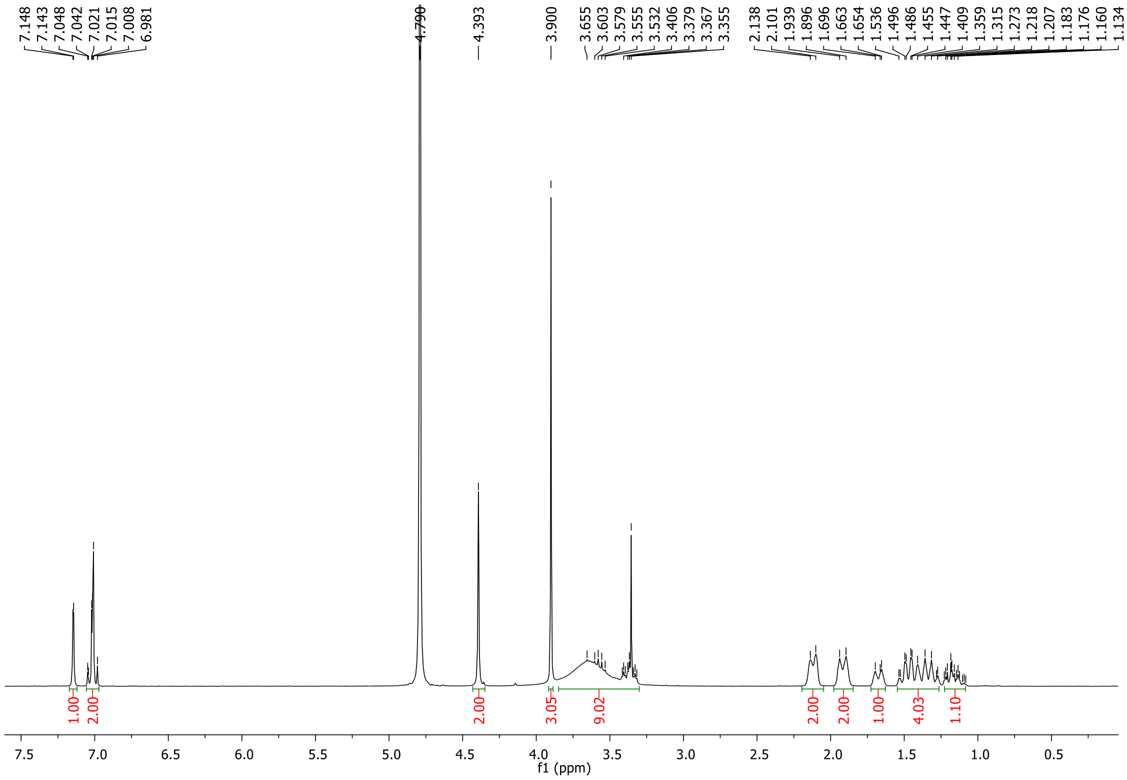

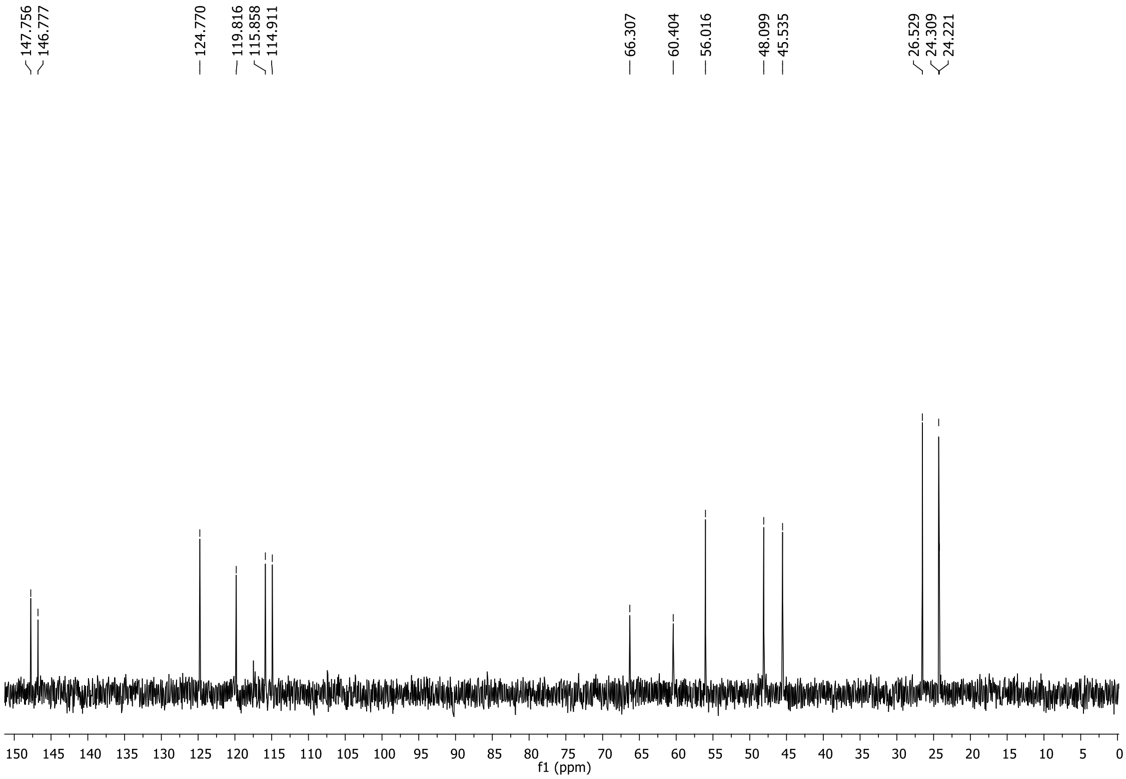

4-((4-((1s,4s)-4-ethylcyclohexyl)piperazin-1-yl)methyl)-2-methoxyphenol (**7**).

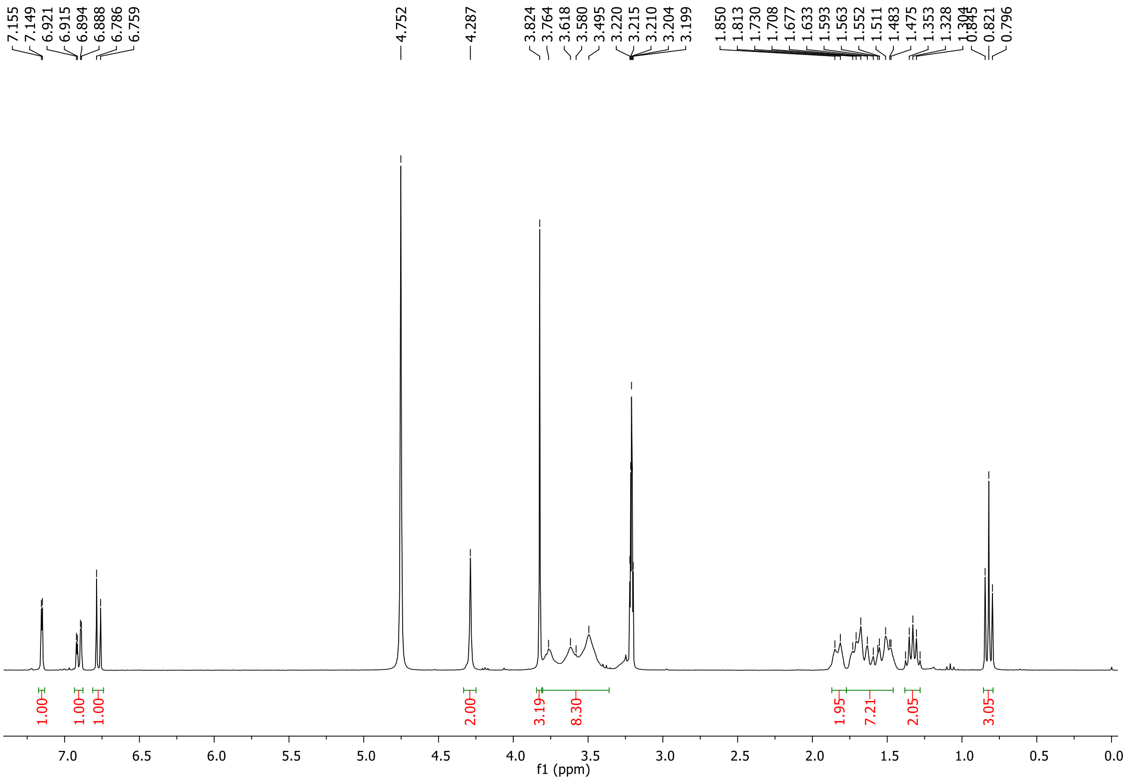

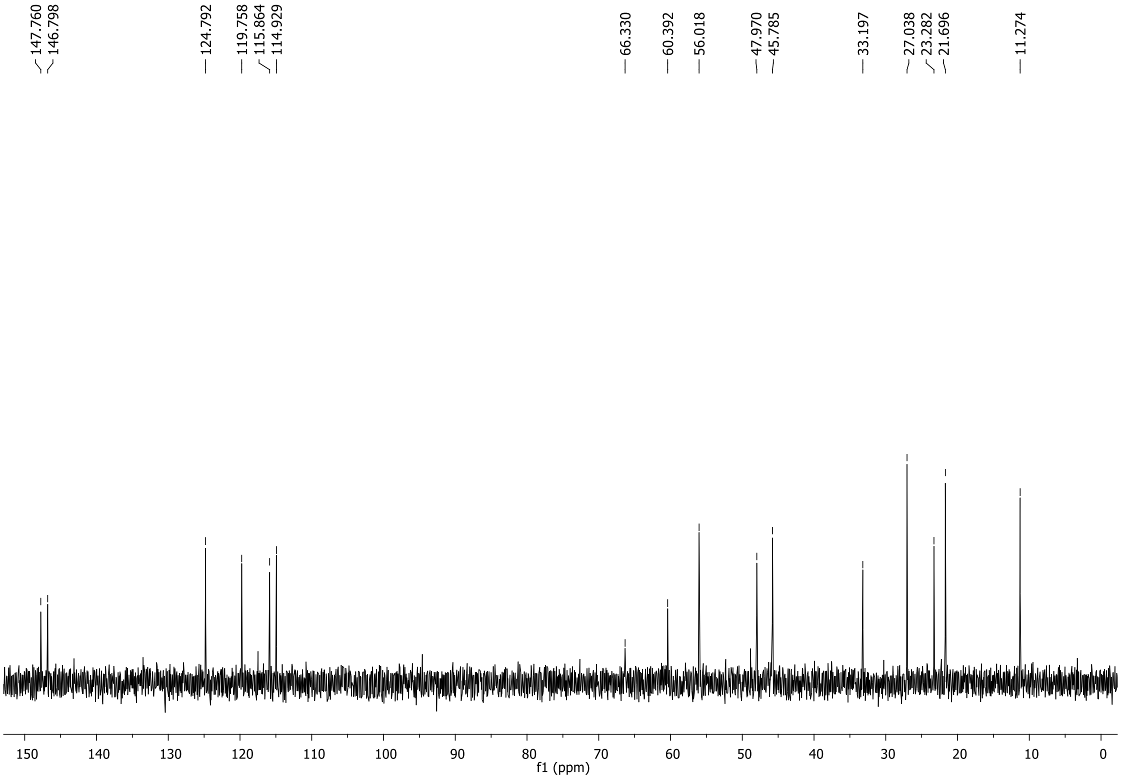

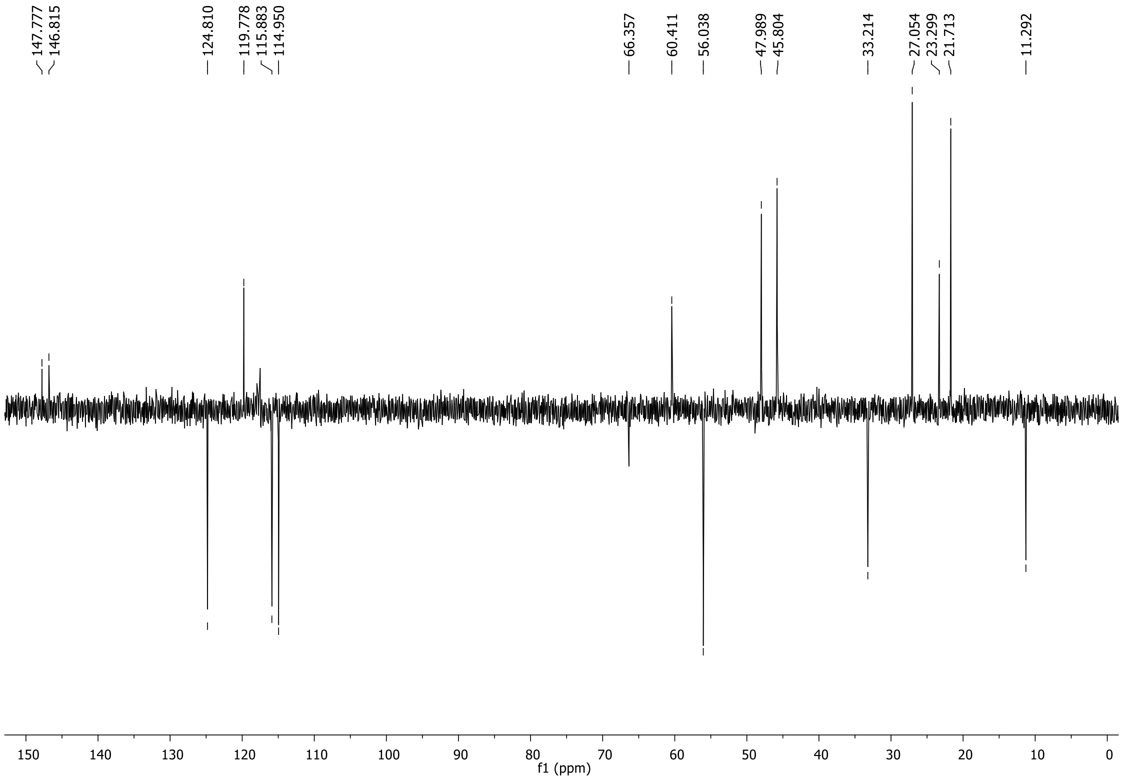

4-((4-((1s,4s)-4-(tert-butyl)cyclohexyl)piperazin-1-yl)methyl)-2-methoxyphenol (**8**).

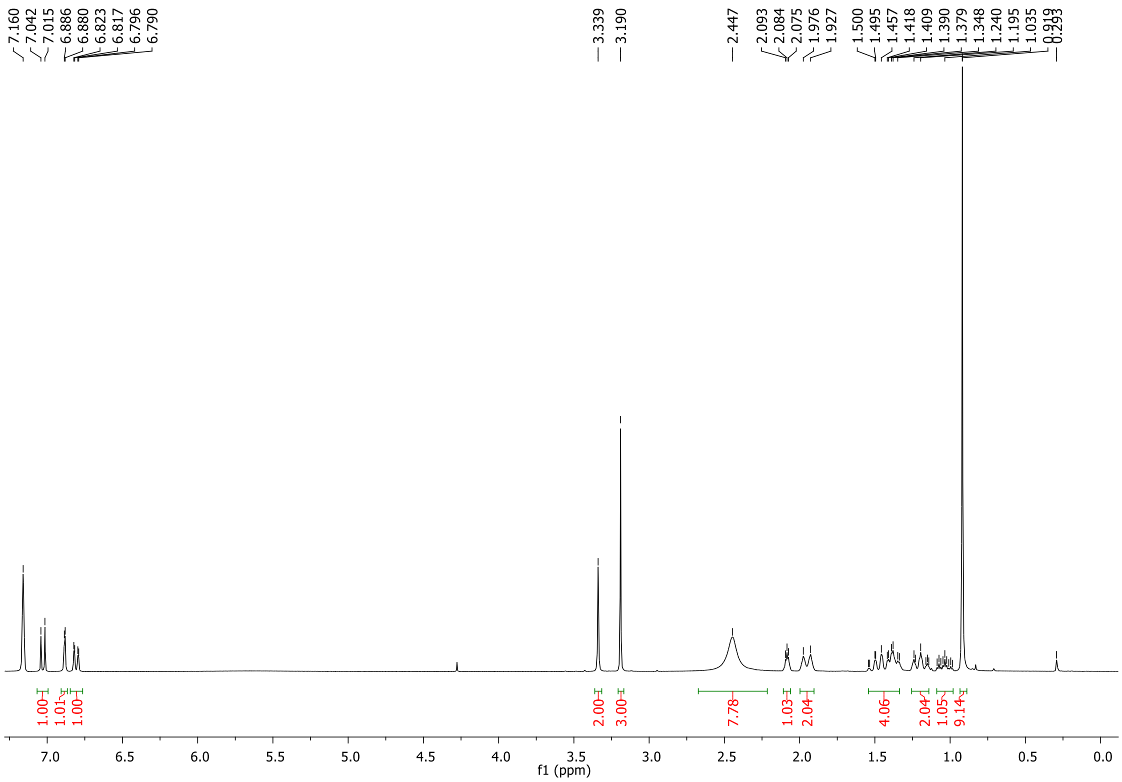

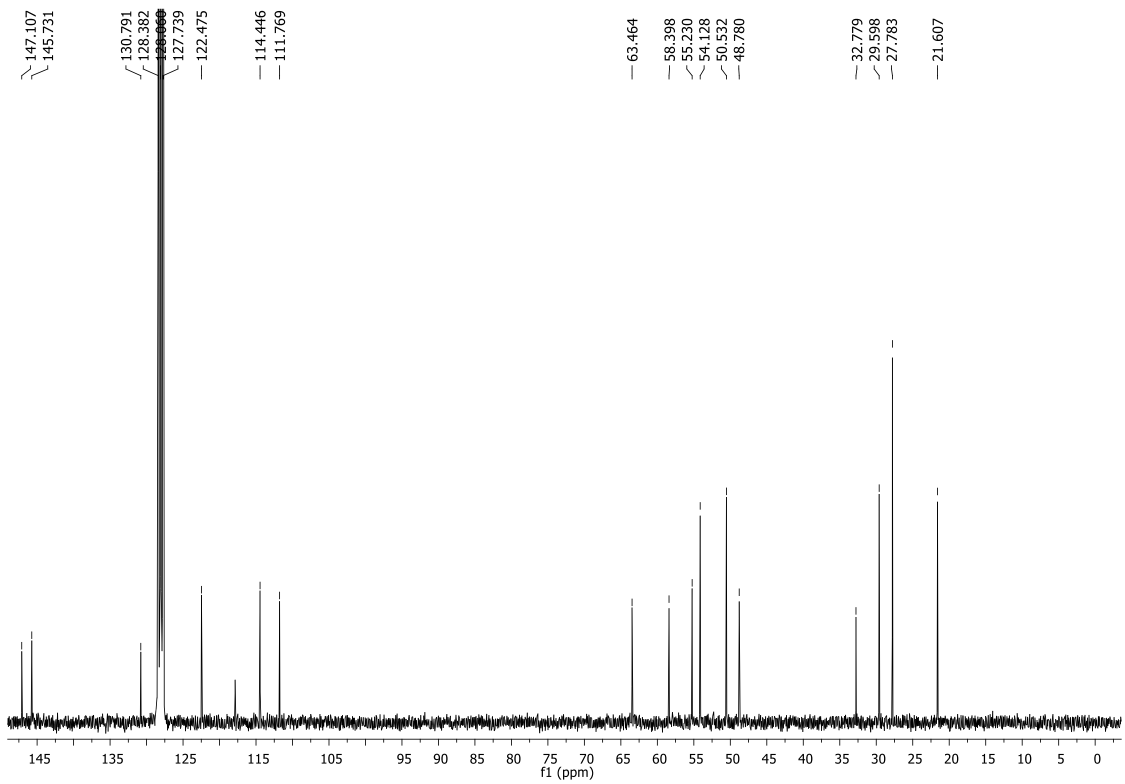
147.107
145.731
130.791
128.382
127.739
122.475
114.446
111.769
63.464
58.398
55.230
54.128
50.532
48.780
32.779
29.598
27.783
21.607
145 135 125 115 105 95 90 85 80 75 70 65 60 55 50 45 40 35 30 25 20 15 10 5 0
f1 (ppm)

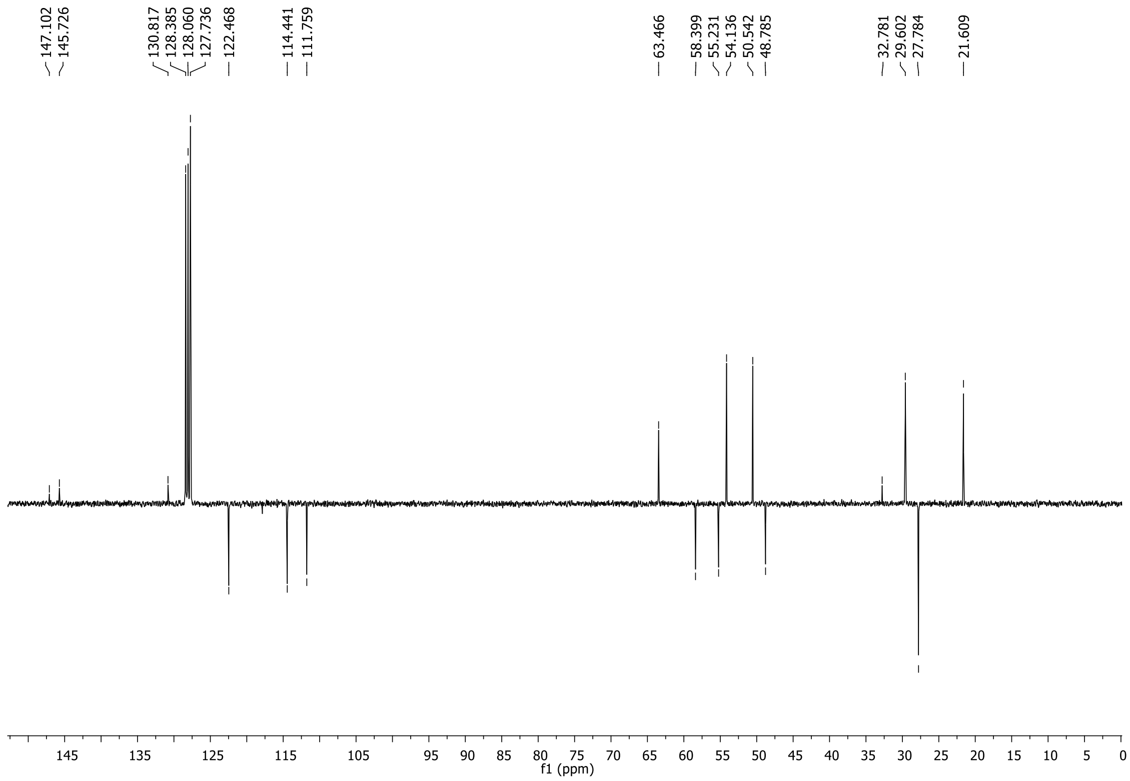
147.102
145.726
130.817
128.385
128.060
127.736
122.468
114.441
111.759
63.466
58.399
55.231
54.136
50.542
48.785
32.781
29.602
27.784
21.609
145 135 125 115 105 95 90 85 80 75 70 65 60 55 50 45 40 35 30 25 20 15 10 5 0
f1 (ppm)

2-methoxy-4-((4-((1s,4s)-4-phenylcyclohexyl)piperazin-1-yl)methyl)phenol (**9**).

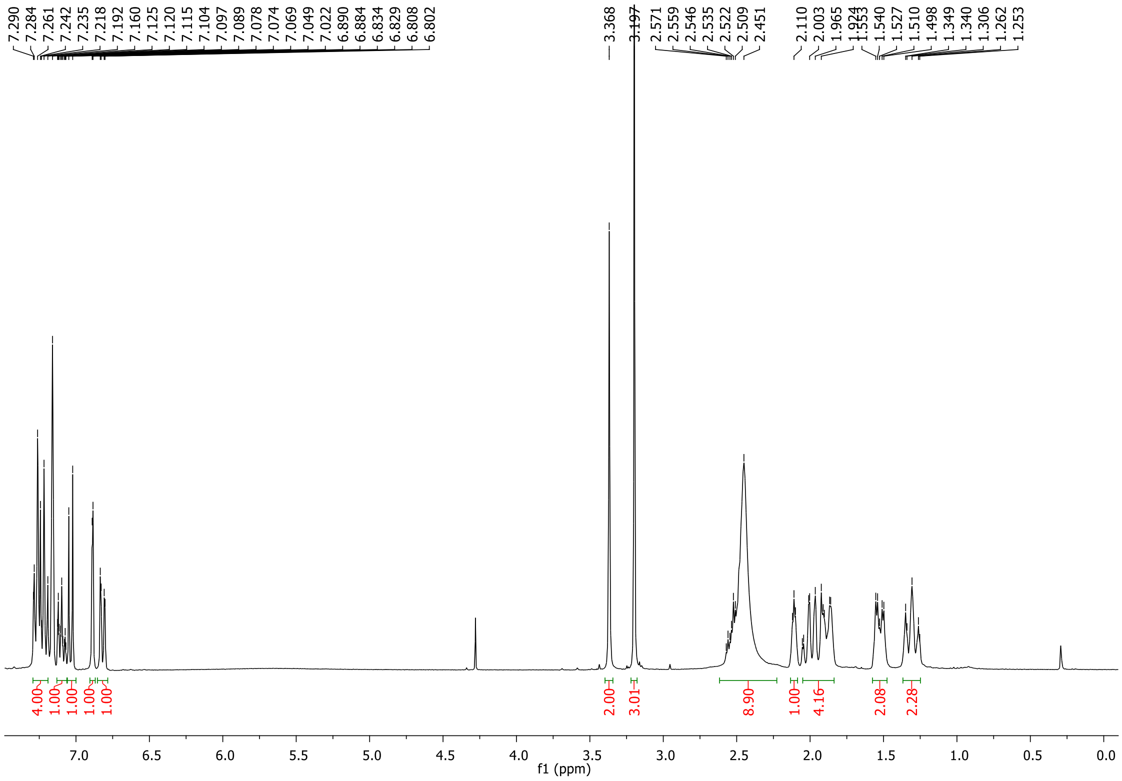

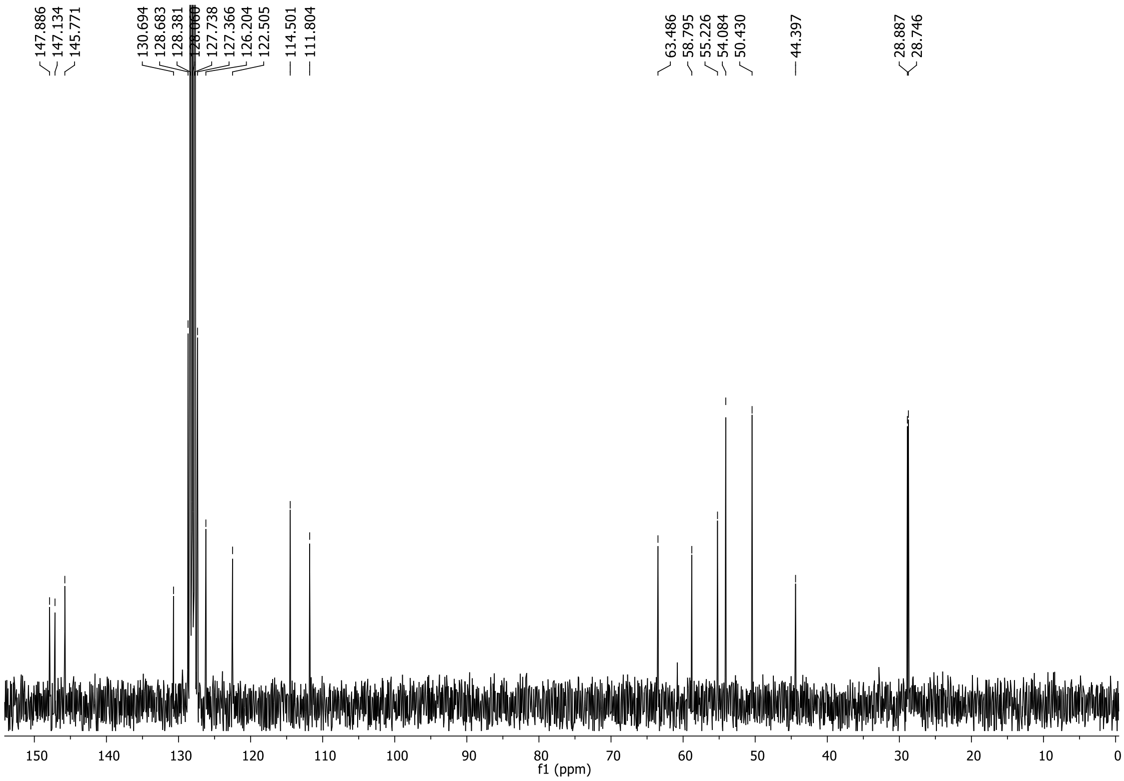

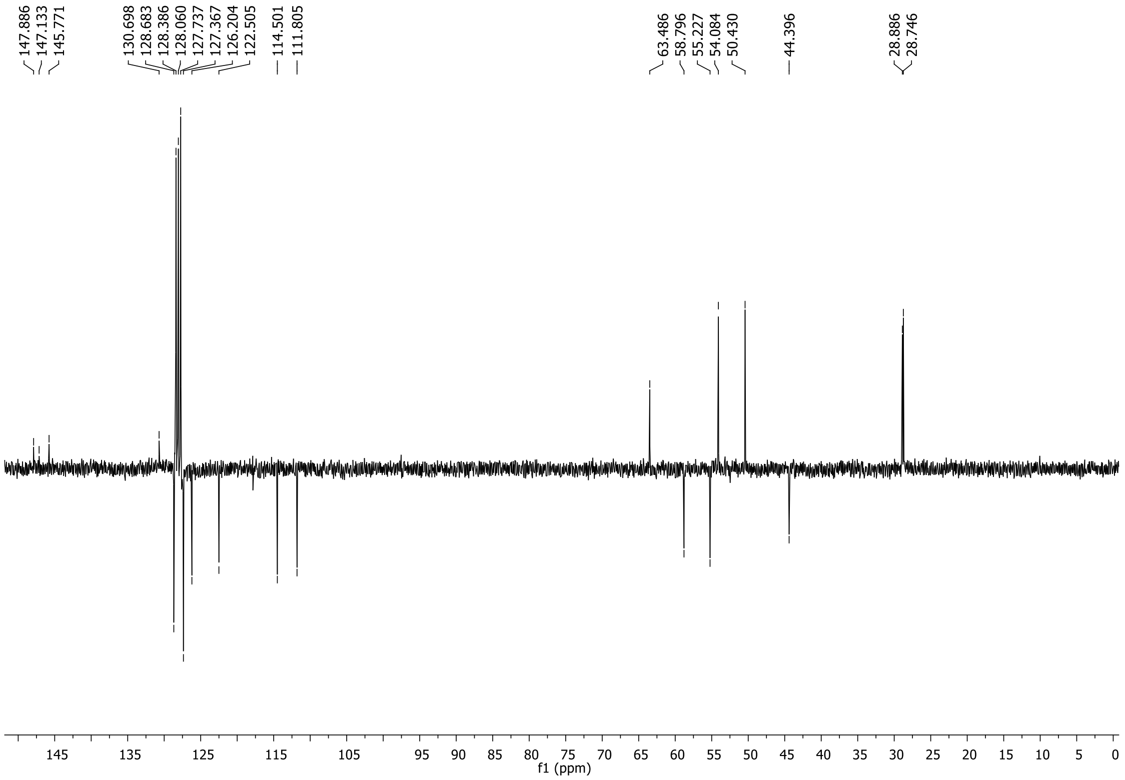

(4-cyclohexylpiperazin-1-yl)(4-hydroxy-3-methoxyphenyl)methanone (**11**).

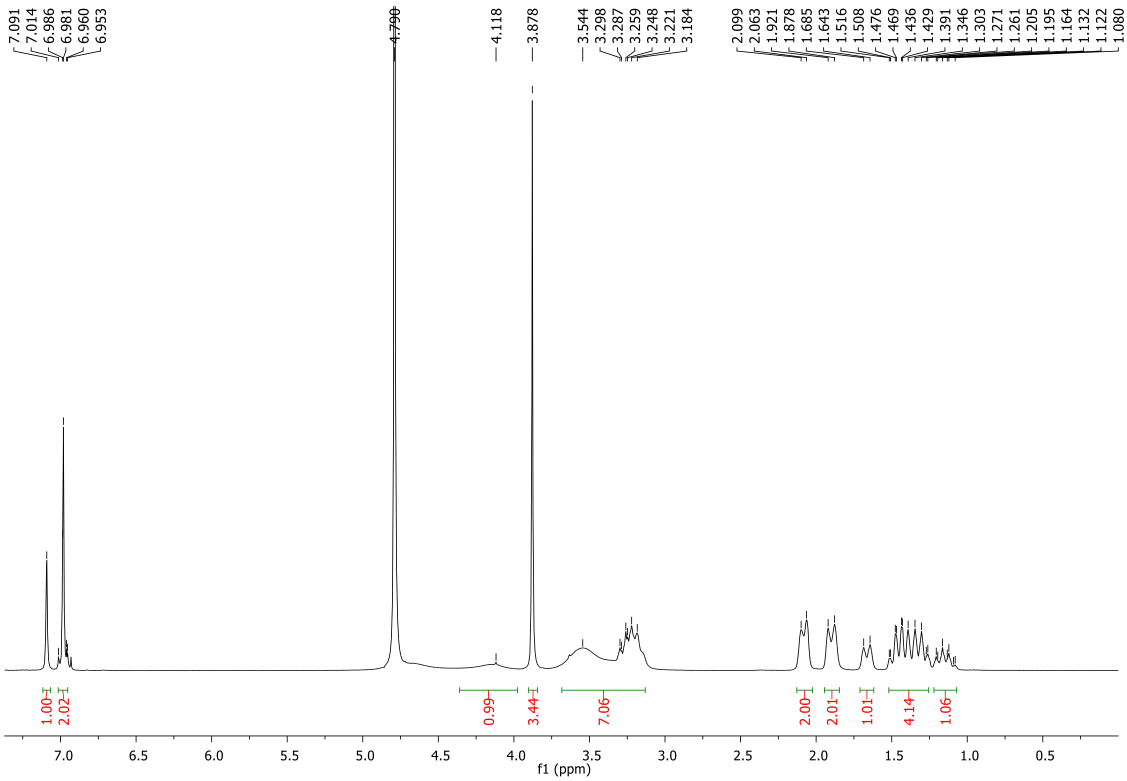

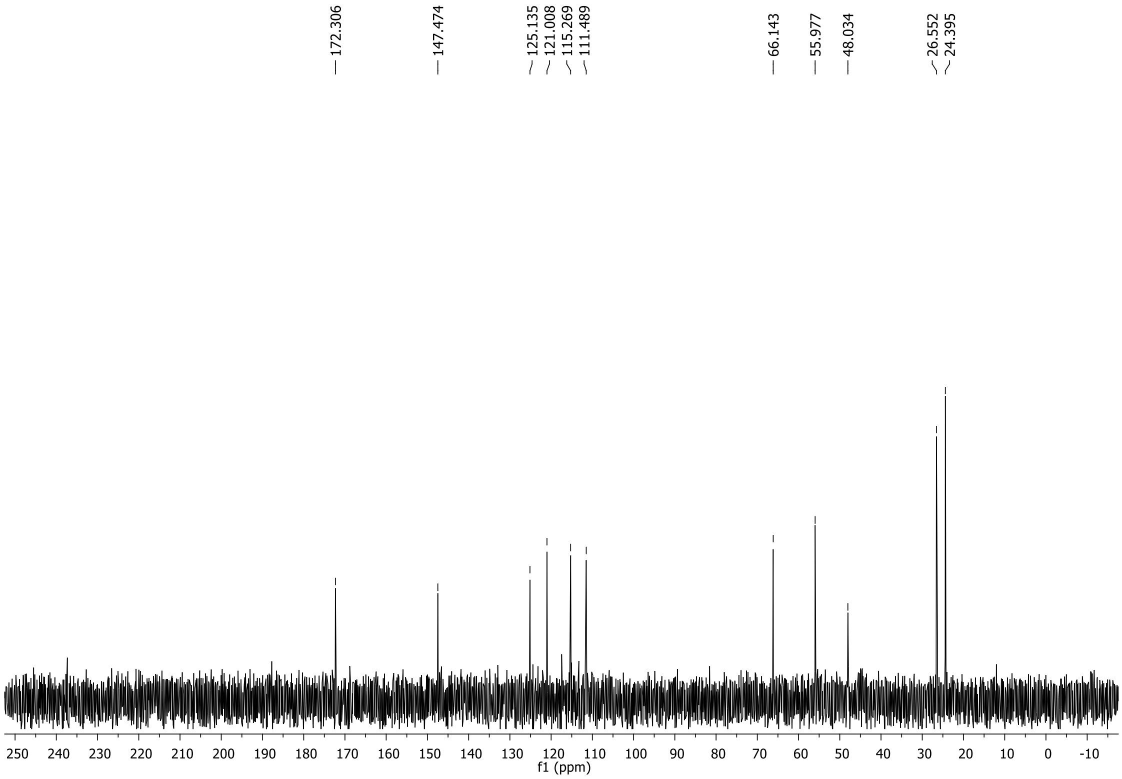

(4-((1s,4s)-4-ethylcyclohexyl)piperazin-1-yl)(4-hydroxy-3-methoxyphenyl)methanone (**12**).

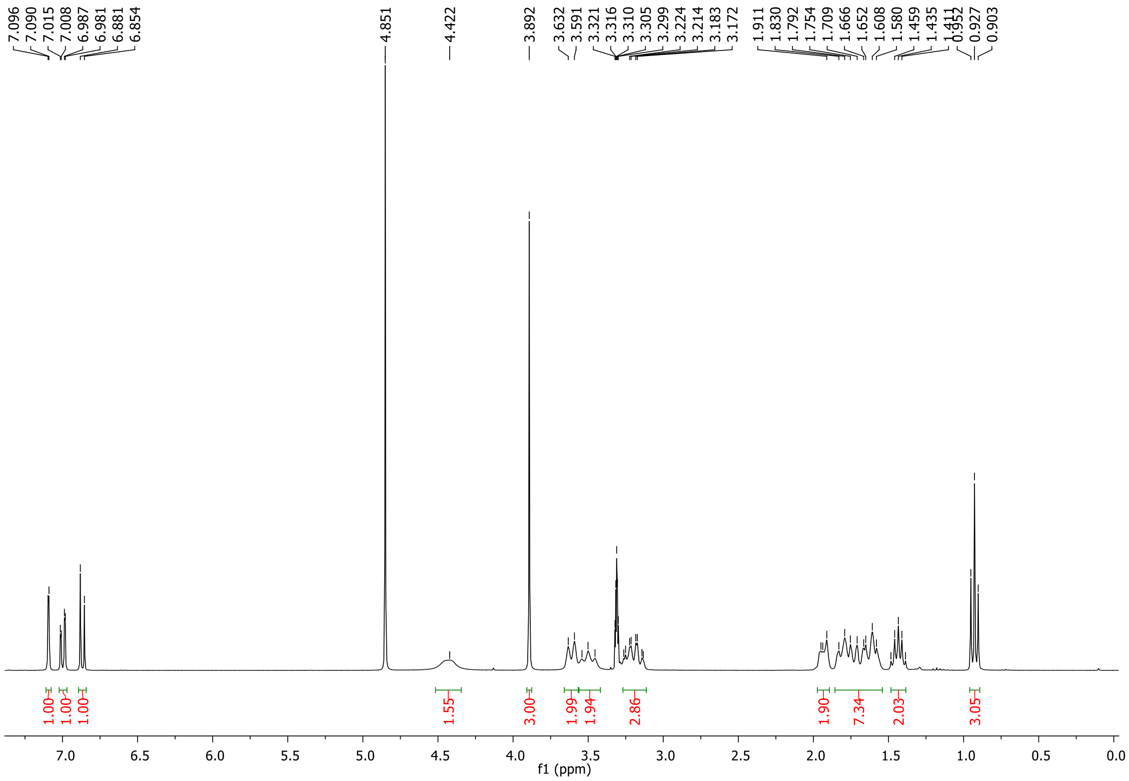

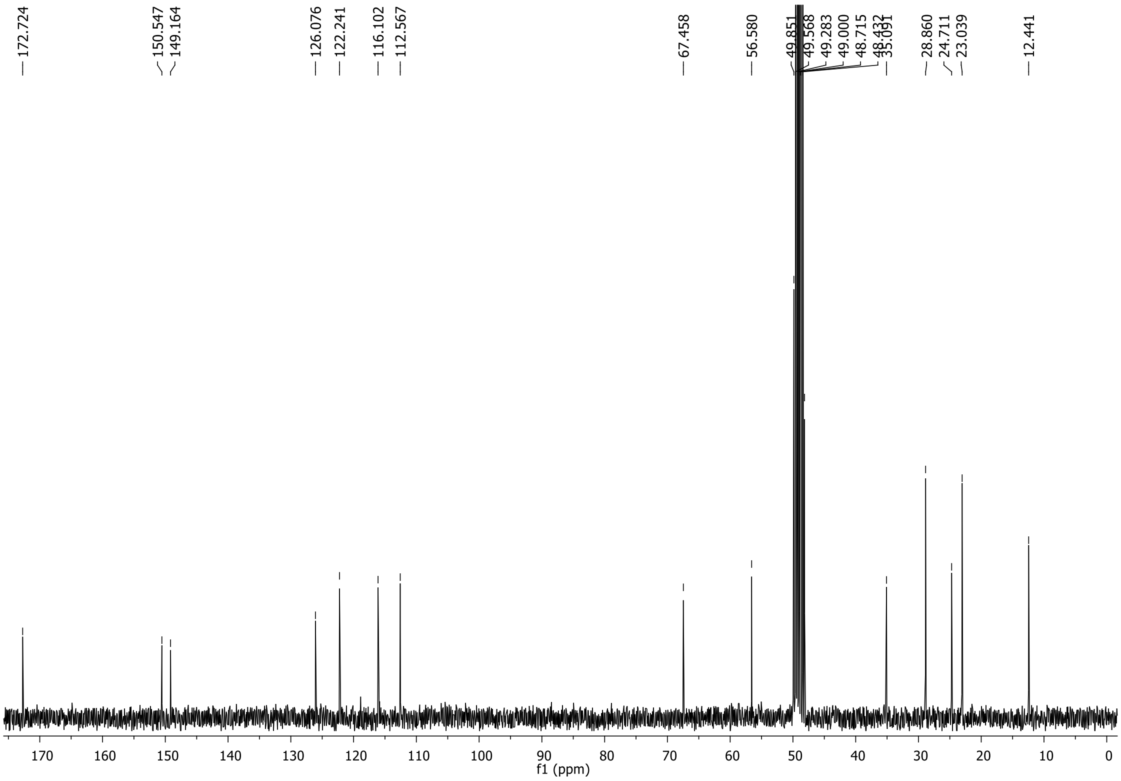
172.724
150.547
149.164
126.076
122.241
116.102
112.567
67.458
56.580
49.851
49.568
49.283
49.000
48.715
48.432
35.091
28.860
24.711
23.039
12.441
170 160 150 140 130 120 110 100 90 80 70 60 50 40 30 20 10 0
f1 (ppm)

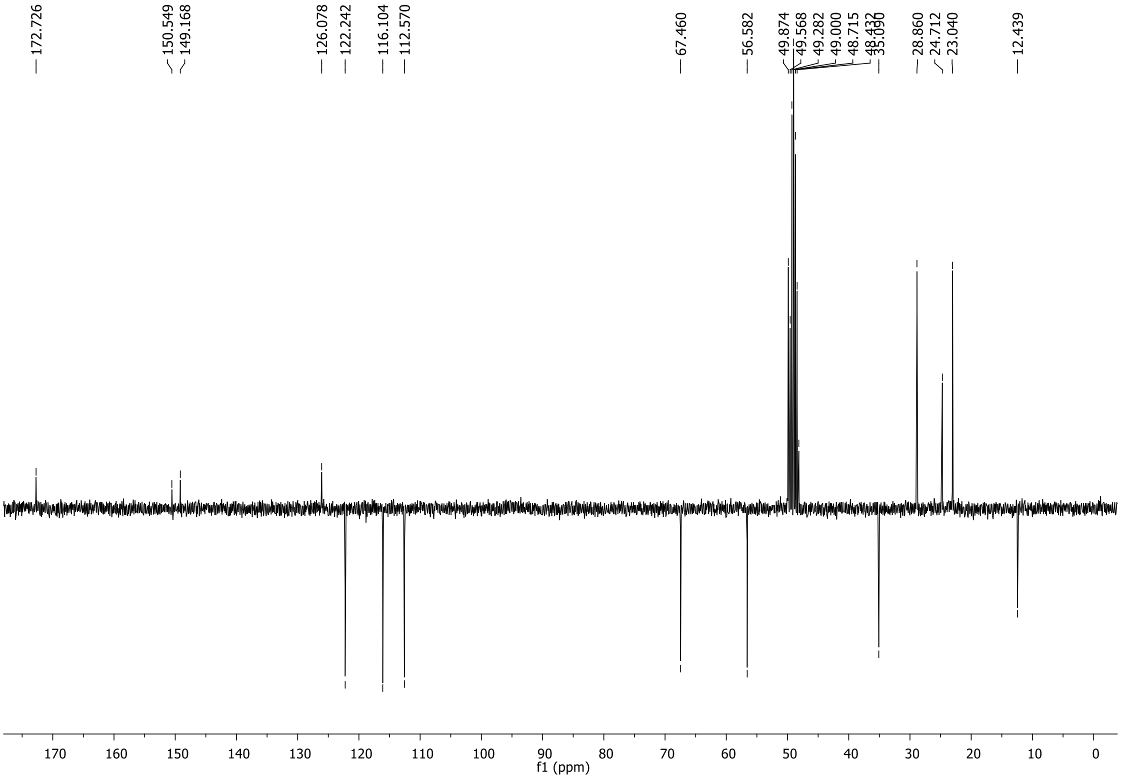
172.726
150.549
149.168
126.078
122.242
116.104
112.570
67.460
56.582
49.874
49.568
49.282
49.000
48.715
48.432
35.090
28.860
24.712
23.040
12.439
170 160 150 140 130 120 110 100 90 80 70 60 50 40 30 20 10 0
f1 (ppm)

(4-((1s,4s)-4-(tert-butyl)cyclohexyl)piperazin-1-yl)(4-hydroxy-3-methoxyphenyl)methanone (**13**).

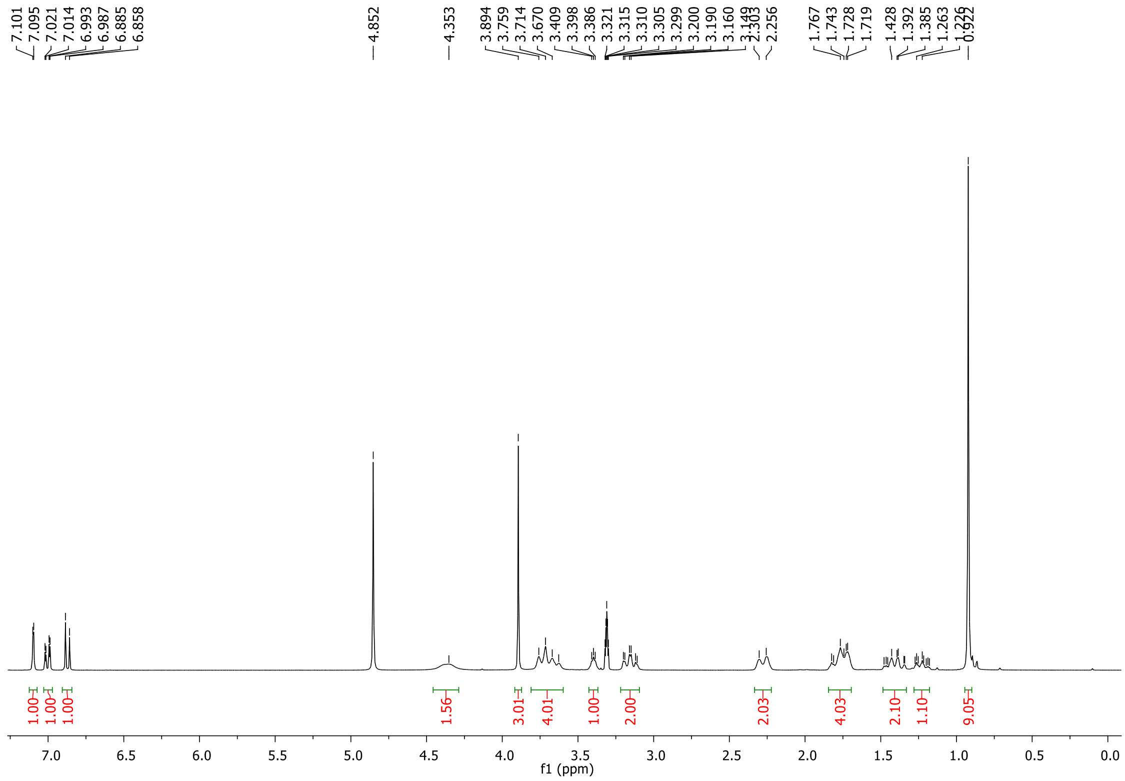

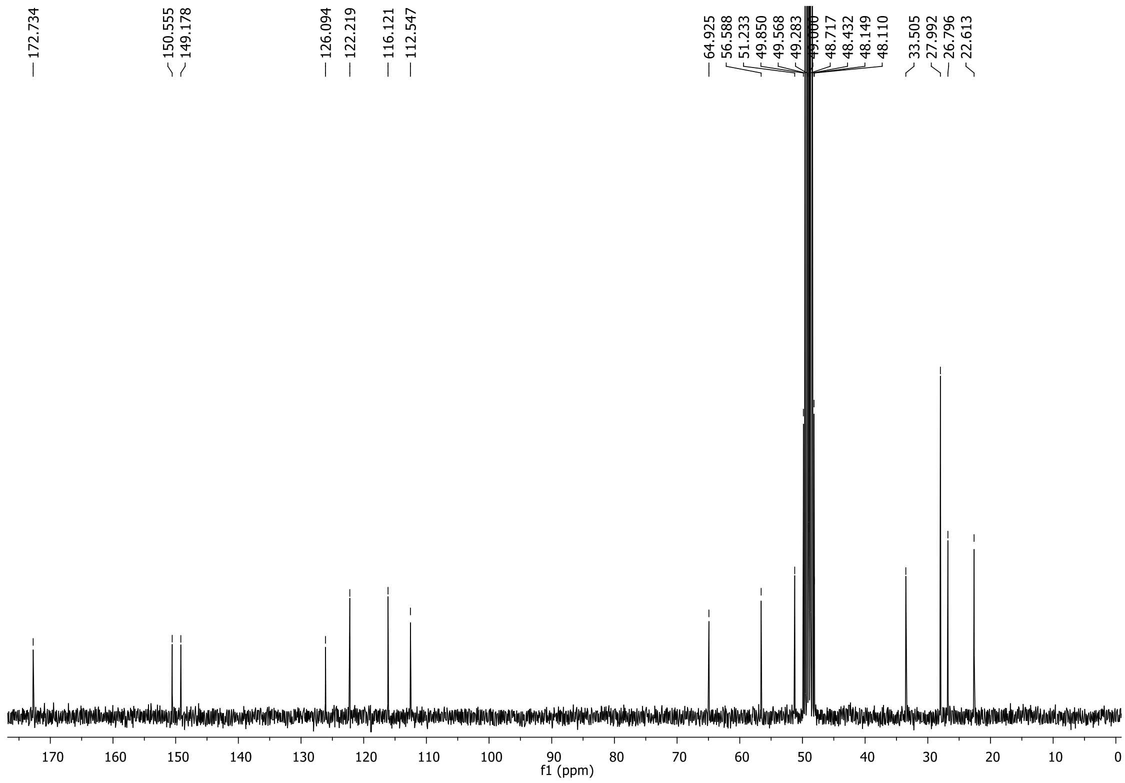

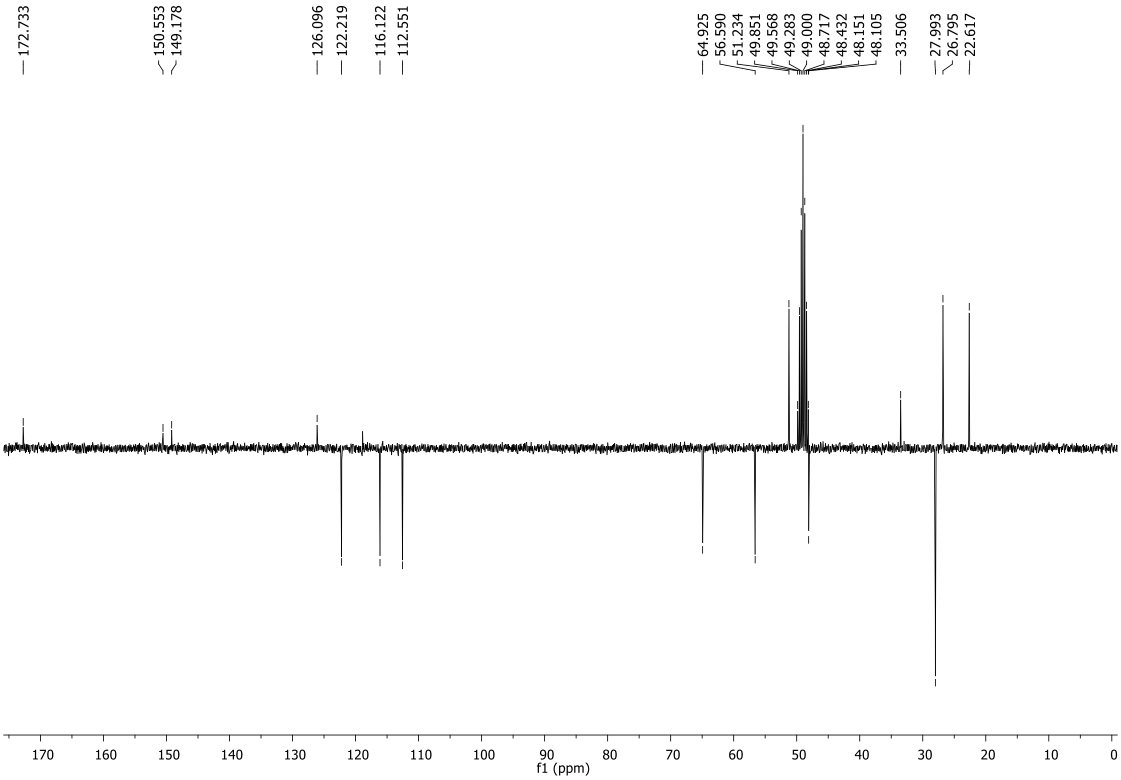

(4-hydroxy-3-methoxyphenyl)(4-((1s,4s)-4-phenylcyclohexyl)piperazin-1-yl)methanone (**14**).

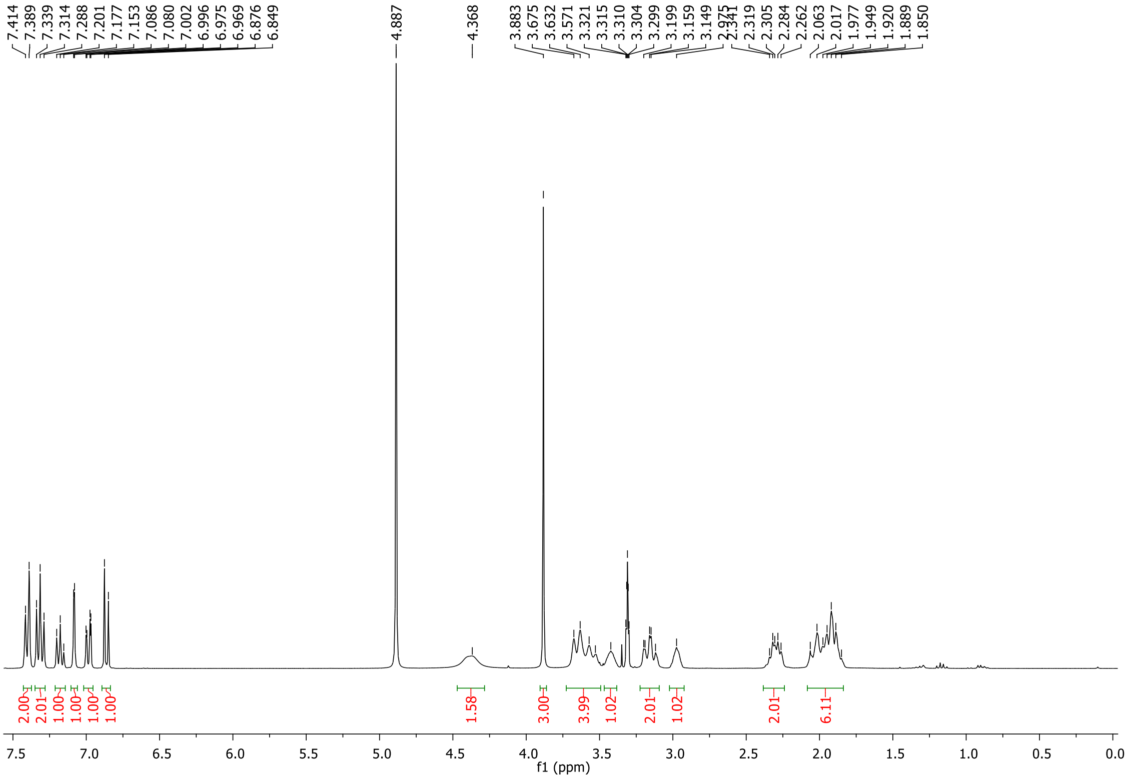

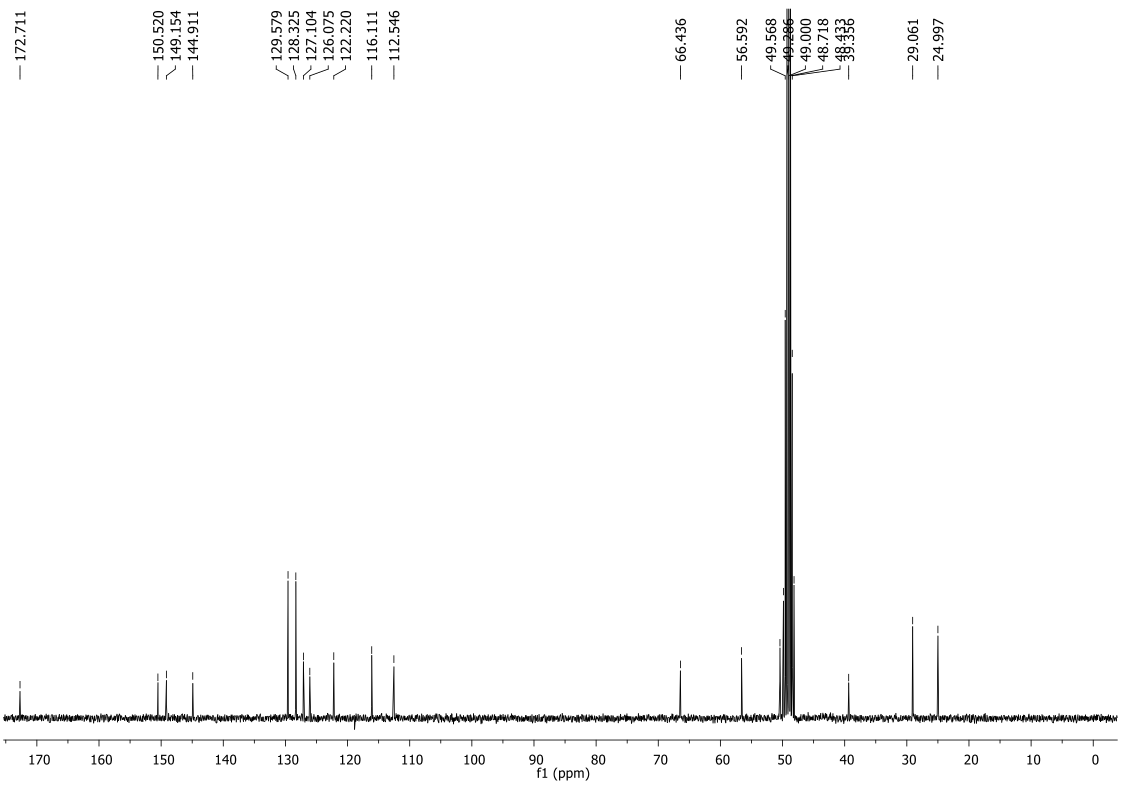

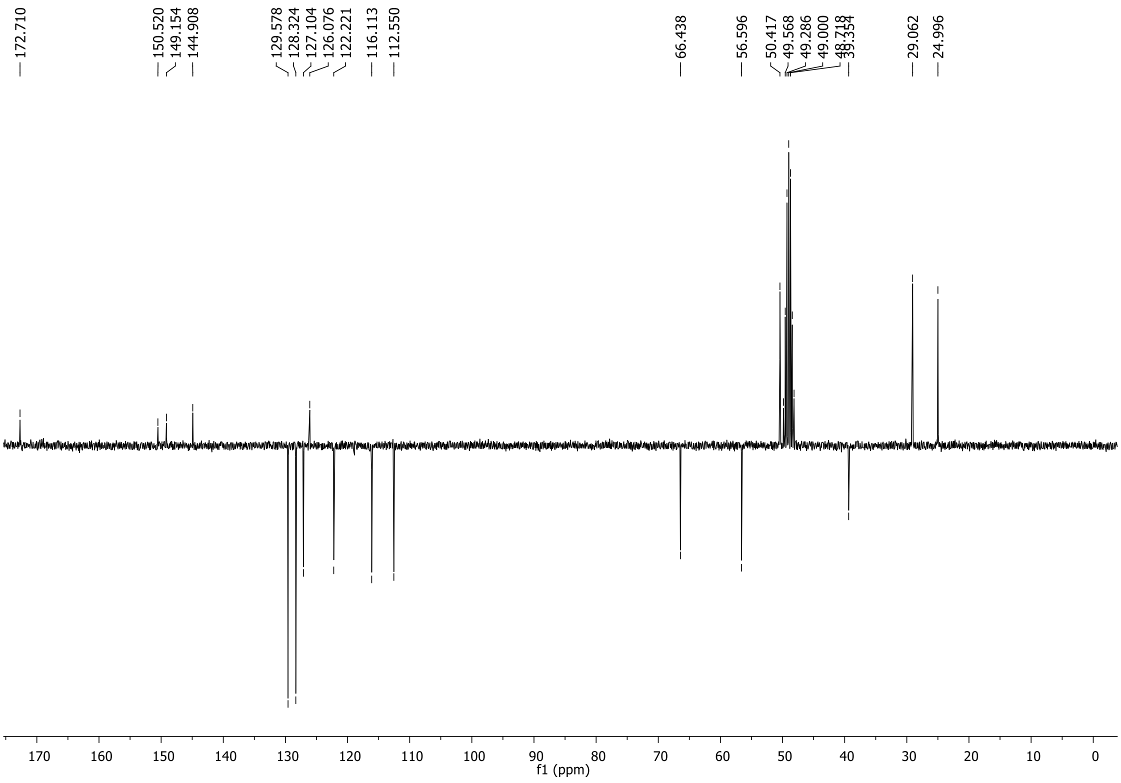

5-((4-((1s,4s)-4-(tert-butyl)cyclohexyl)piperazin-1-yl)methyl)pyridin-2(1H)-one (**16**).

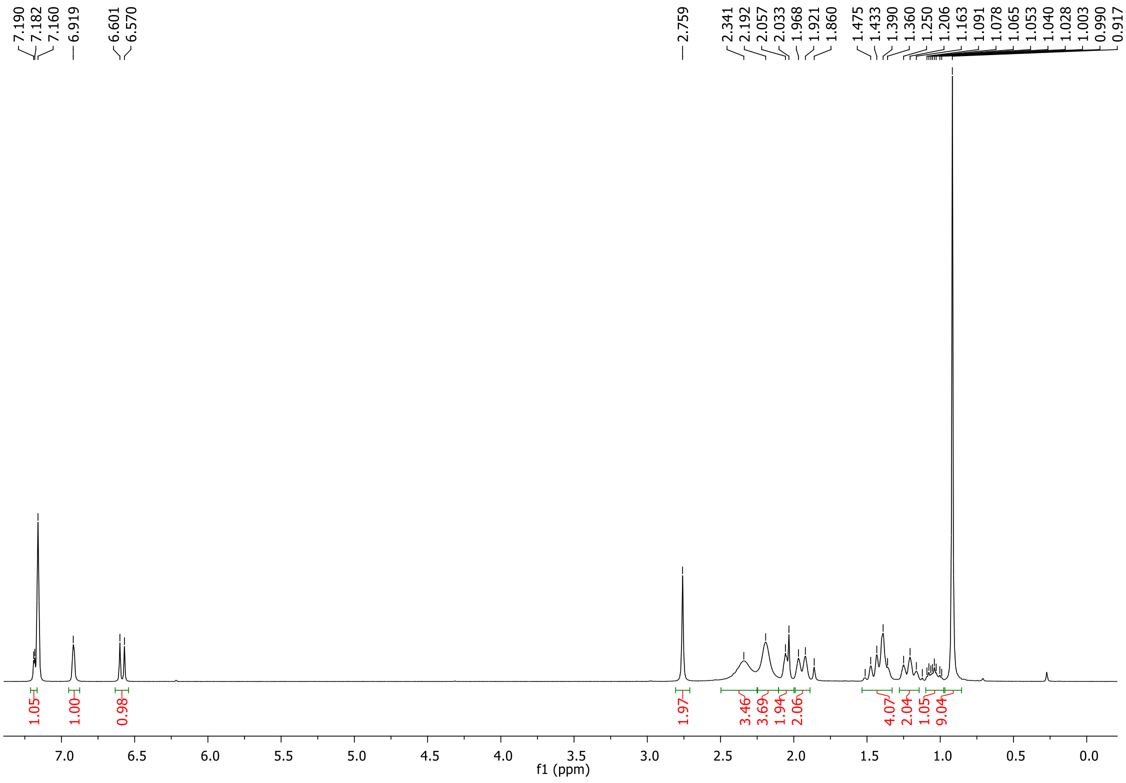

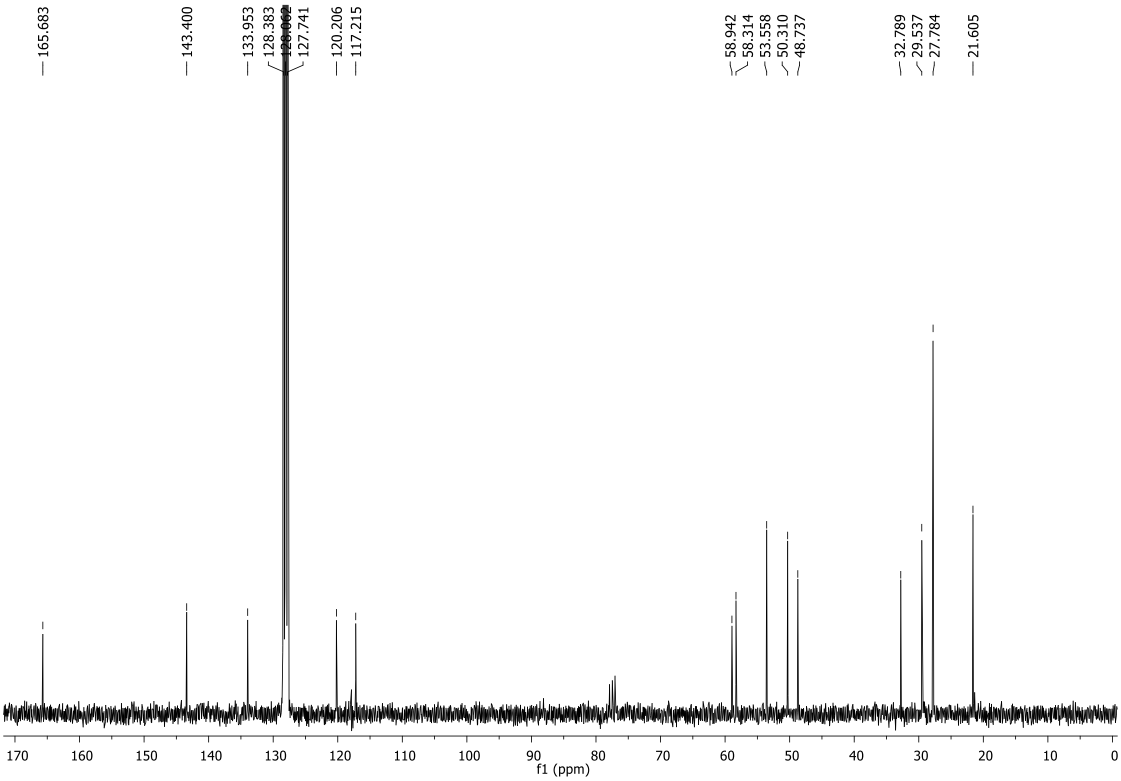

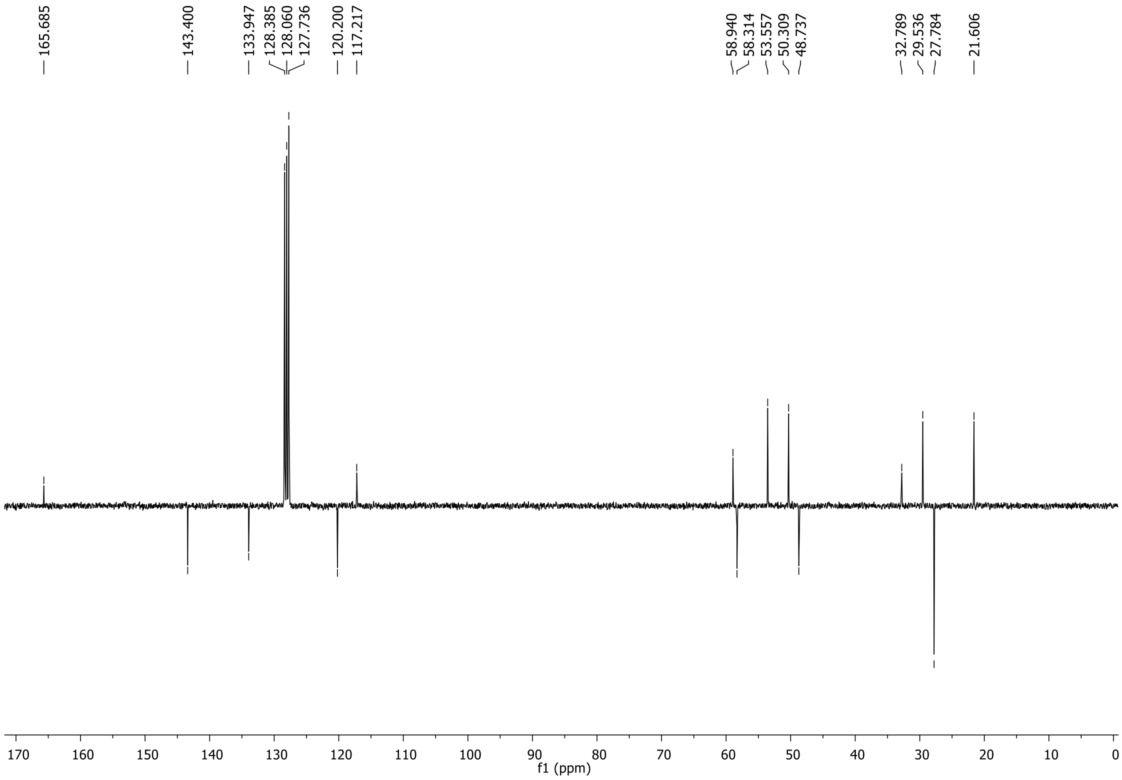

5-((4-((1s,4s)-4-phenylcyclohexyl)piperazin-1-yl)methyl)pyridin-2(1H)-one (**17**).

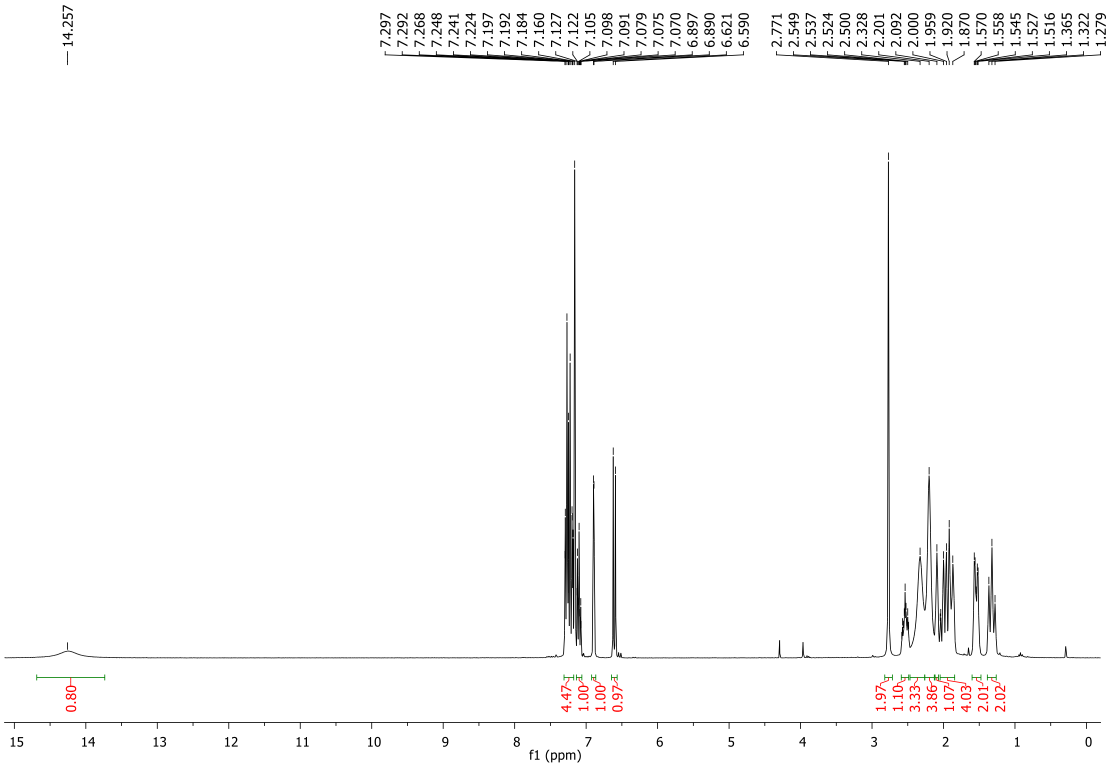

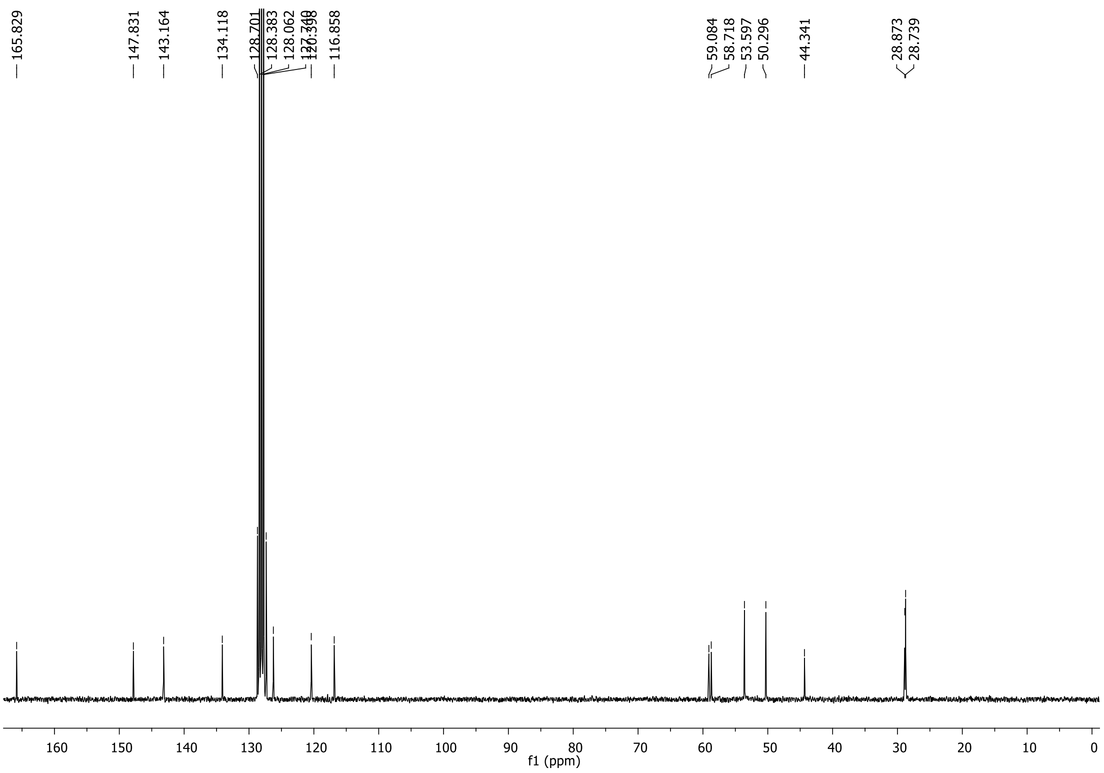
165.829
147.831
143.164
134.118
128.701
128.383
128.062
116.858
59.084
58.718
53.597
50.296
44.341
28.873
28.739
f1 (ppm)

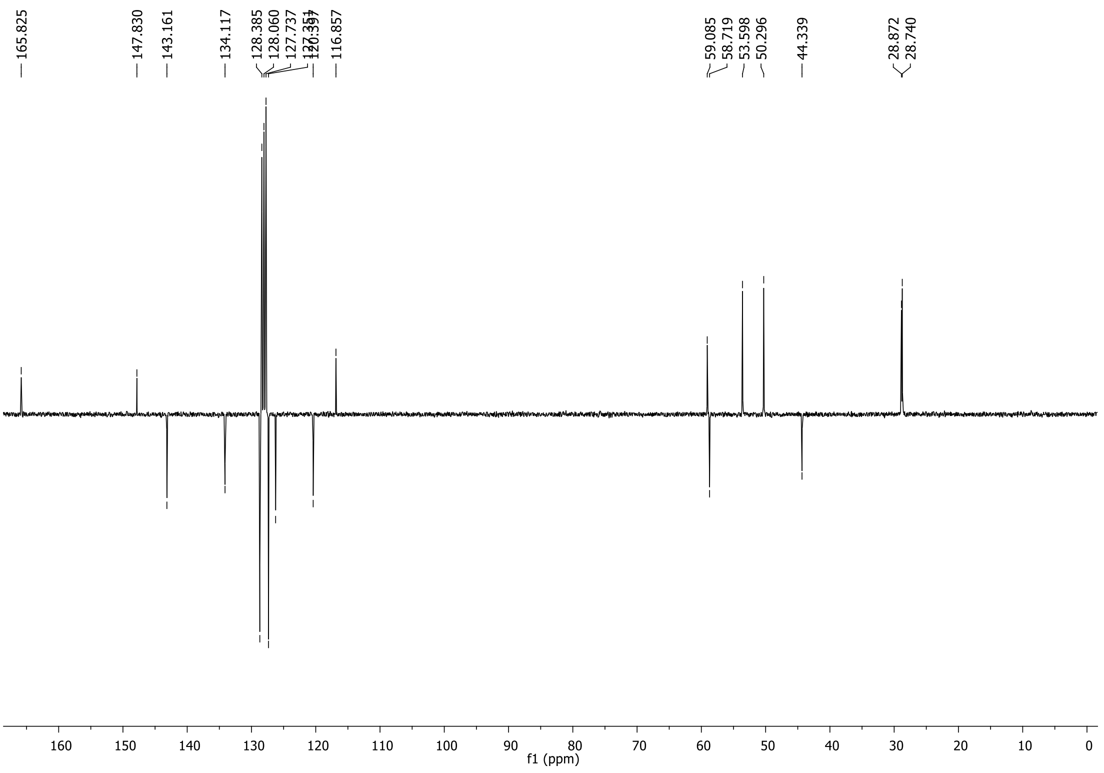
165.825
147.830
143.161
134.117
128.385
128.060
127.737
116.857
59.085
58.719
53.598
50.296
44.339
28.872
28.740
f1 (ppm)

5-(4-((1s,4s)-4-(tert-butyl)cyclohexyl)piperazine-1-carbonyl)pyridin-2(1H)-one (**19**).

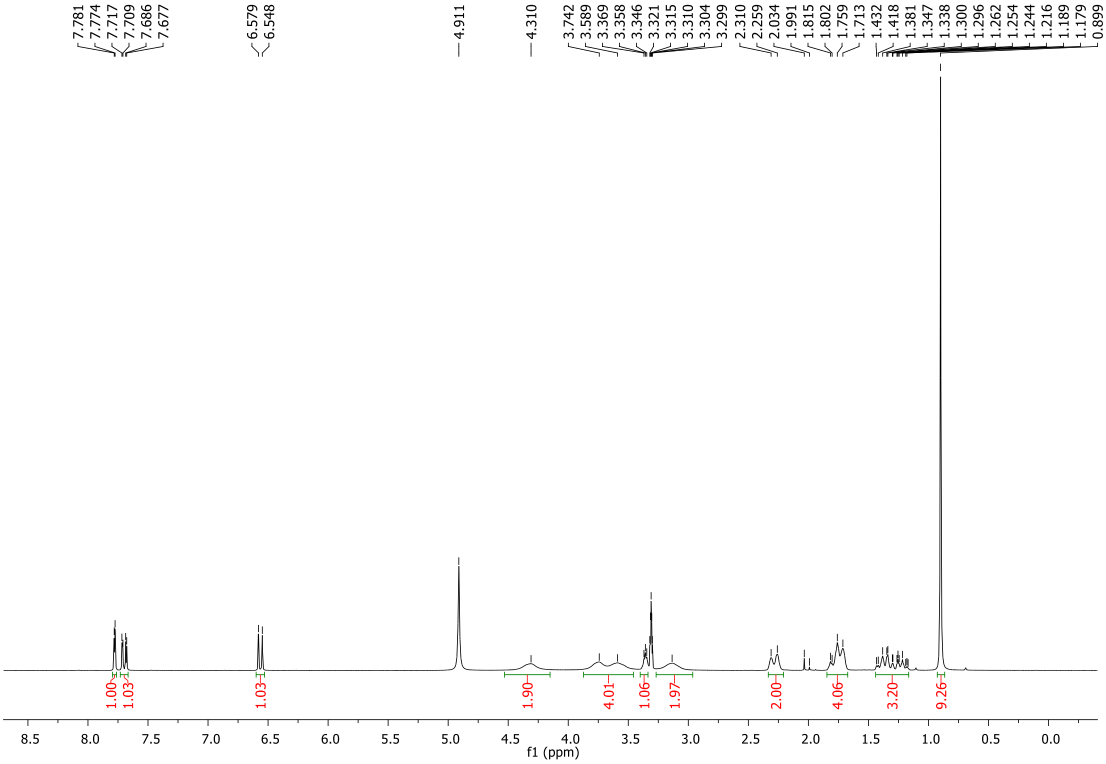

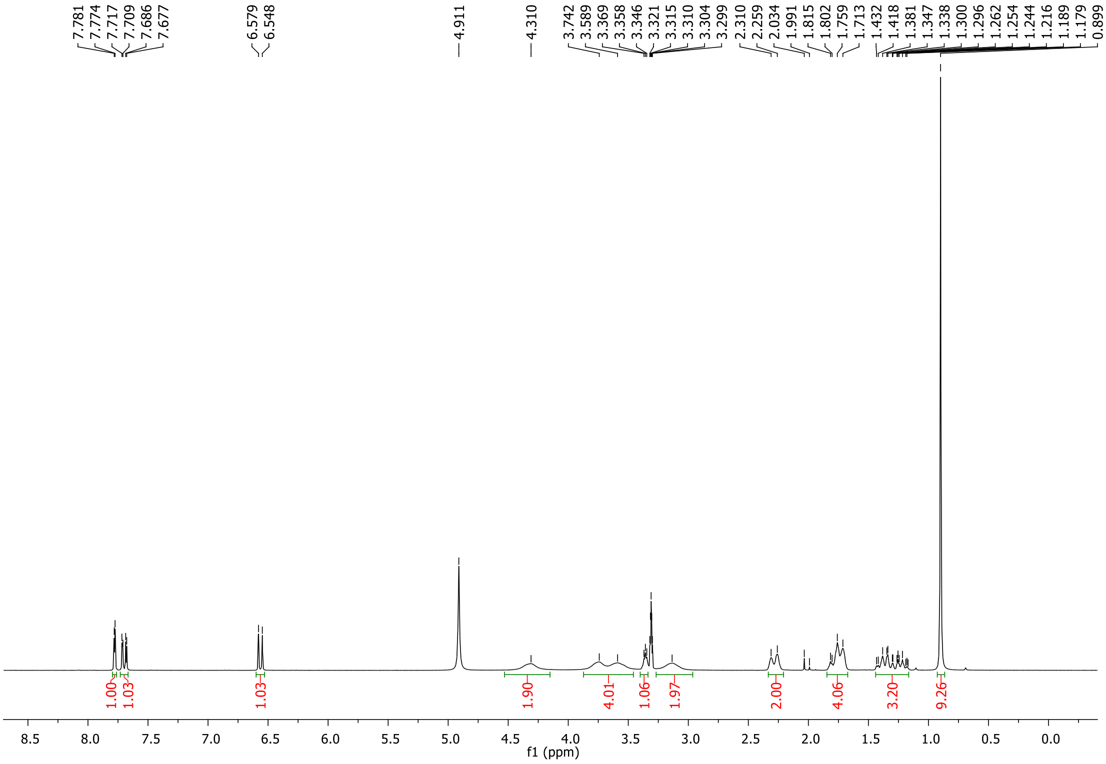

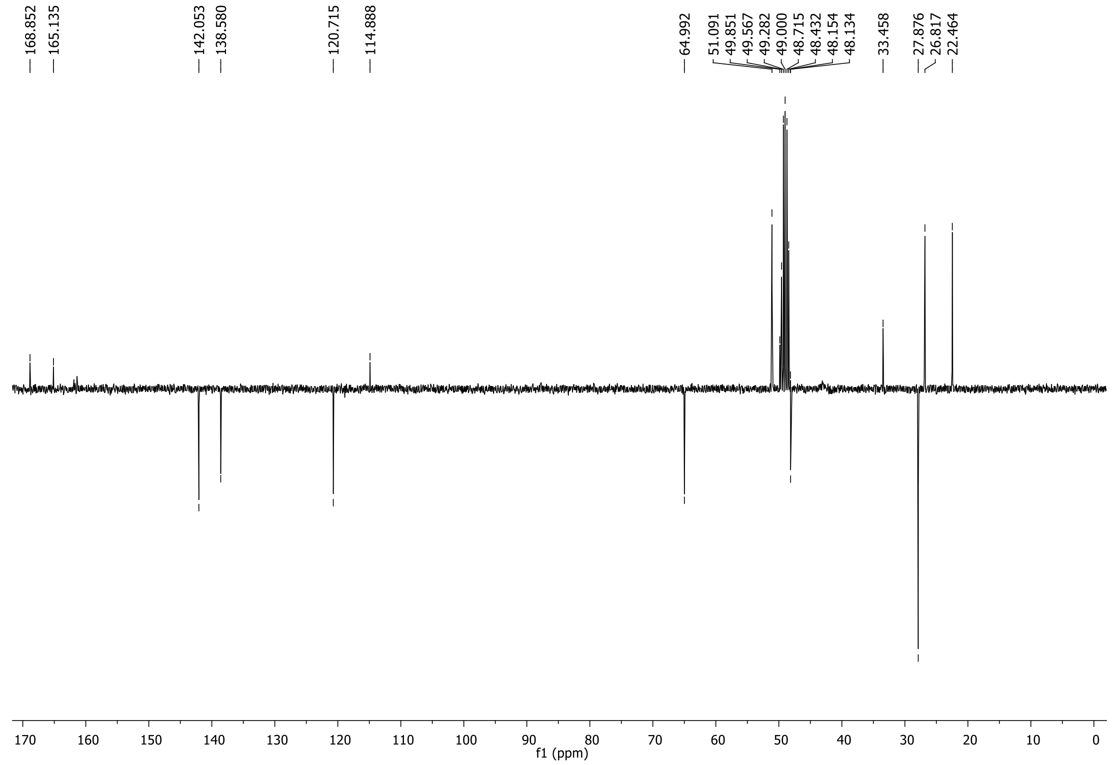

5-(4-((1s,4s)-4-phenylcyclohexyl)piperazine-1-carbonyl)pyridin-2(1H)-one (**20**).

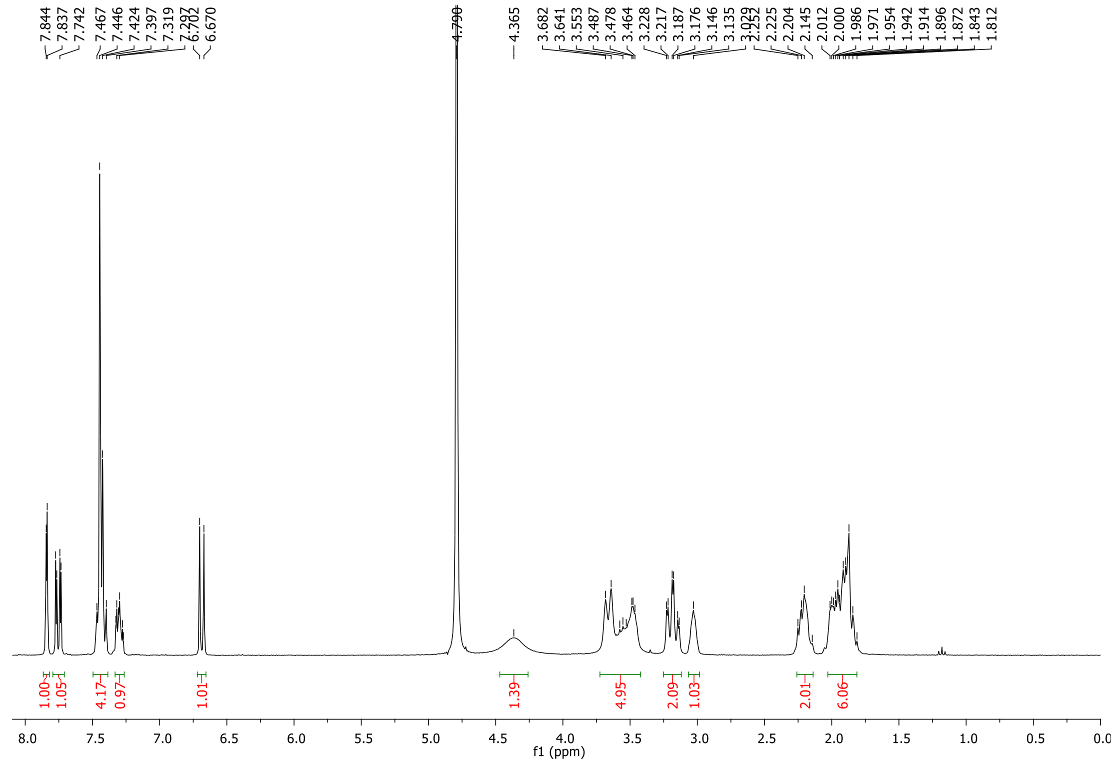

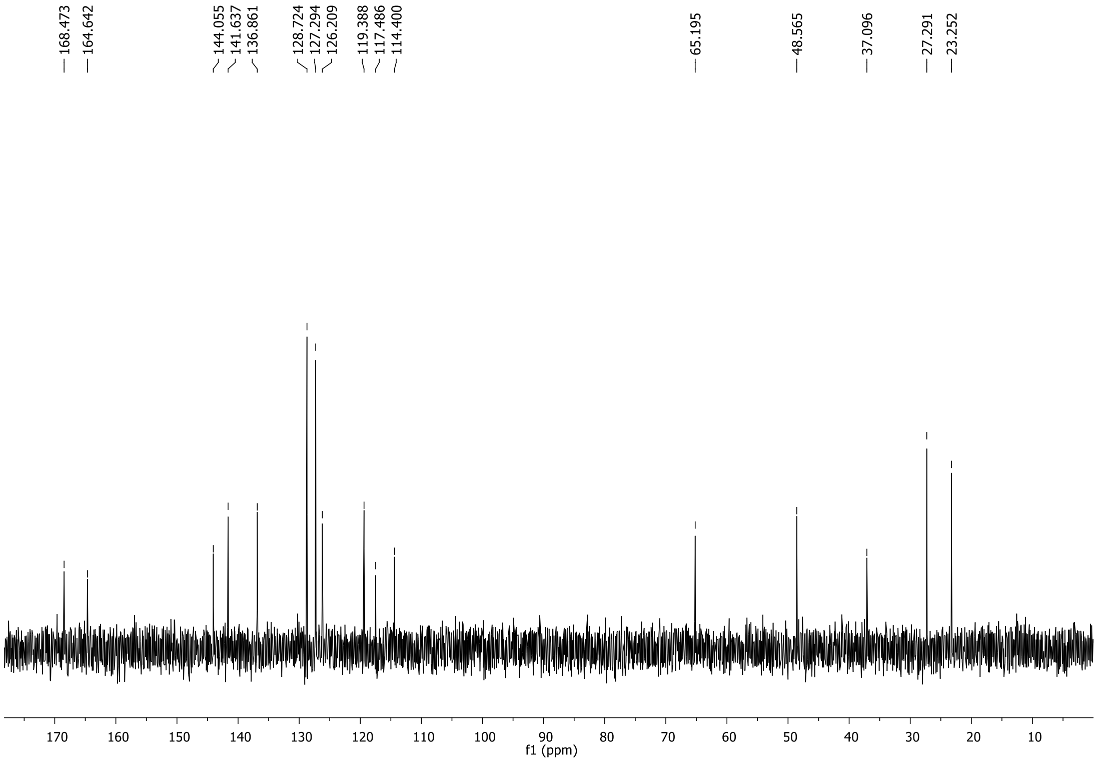
168.473
164.642
144.055
141.637
136.861
128.724
127.294
126.209
119.388
117.486
114.400
65.195
48.565
37.096
27.291
23.252
170 160 150 140 130 120 110 100 90 80 70 60 50 40 30 20 10
f1 (ppm)

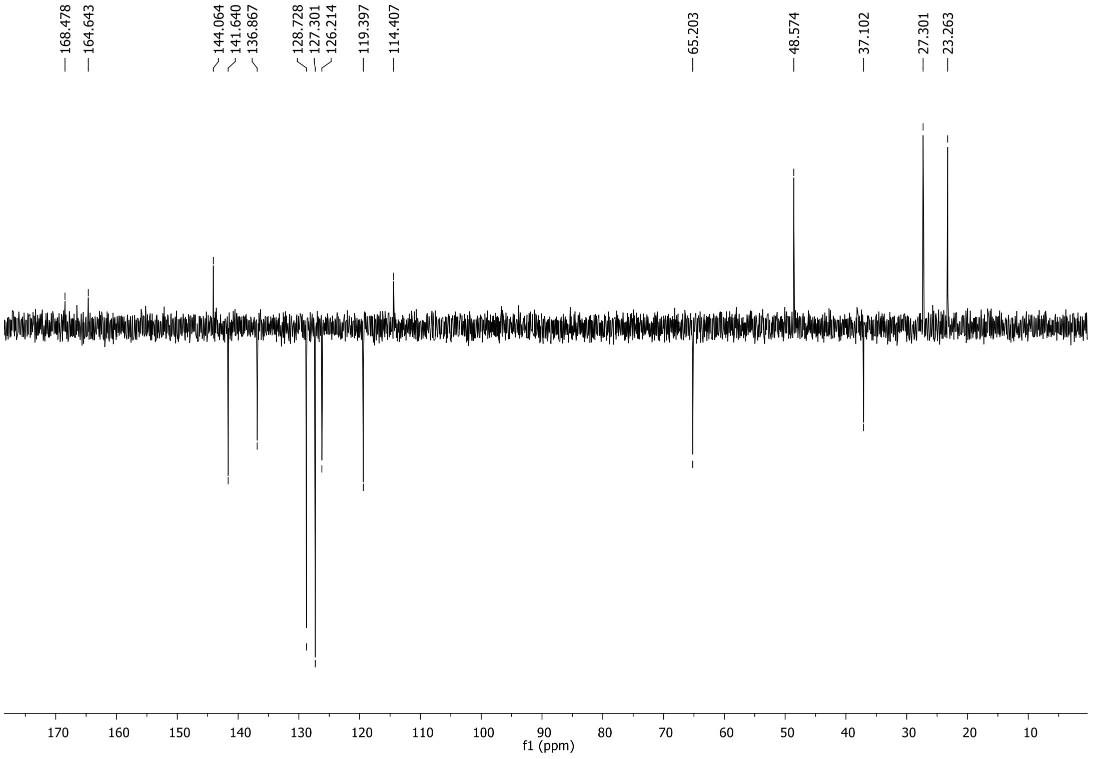
168.478
164.643
144.064
141.640
136.867
128.728
127.301
126.214
119.397
114.407
65.203
48.574
37.102
27.301
23.263
170 160 150 140 130 120 110 100 90 80 70 60 50 40 30 20 10
f1 (ppm)

1-((1s,4s)-4-phenylcyclohexyl)-4-(pyridin-3-ylmethyl)piperazine (**24**).

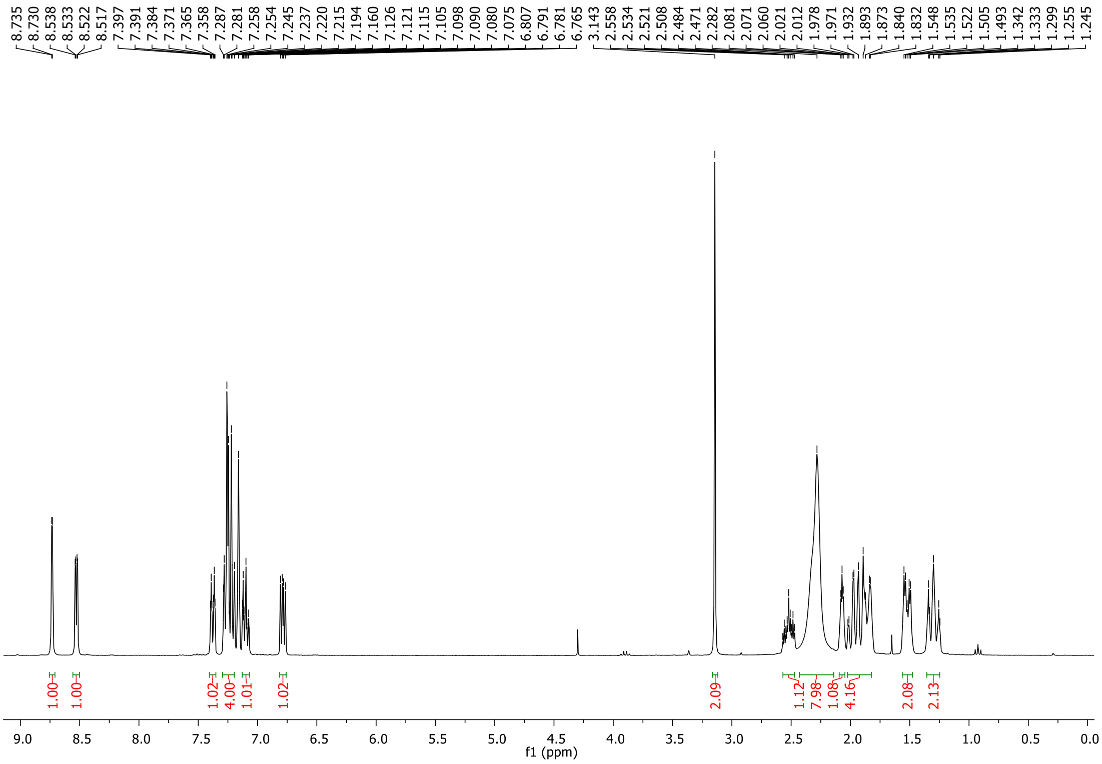

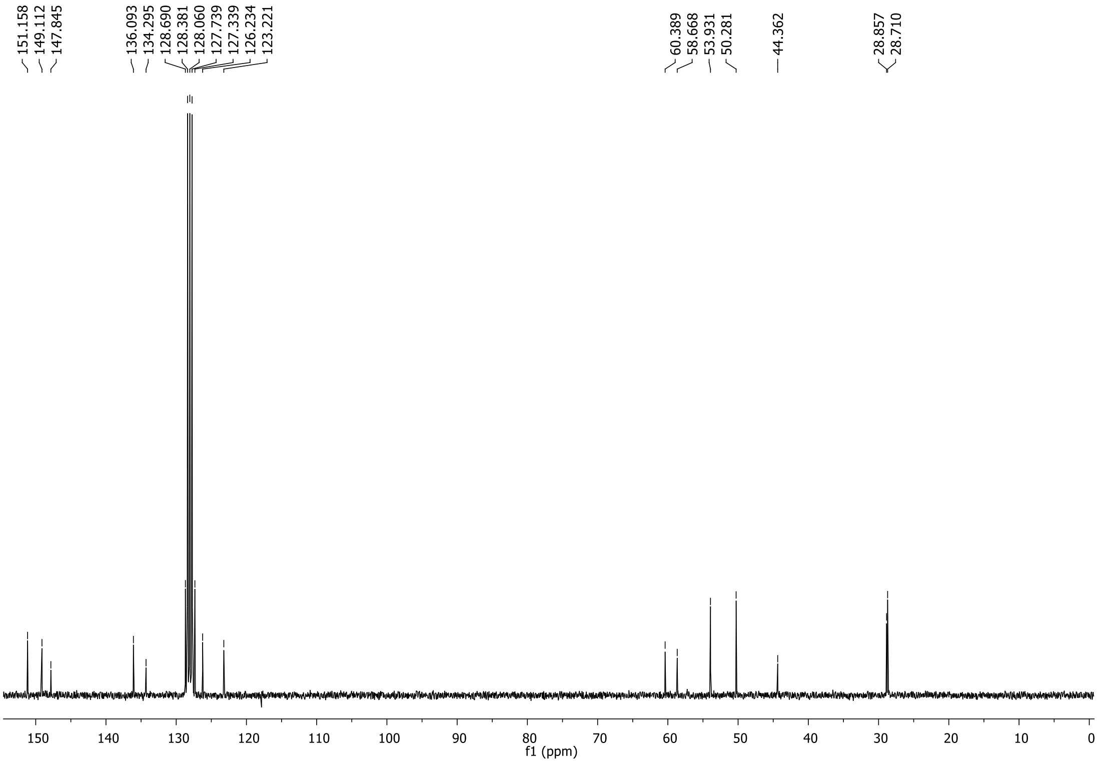

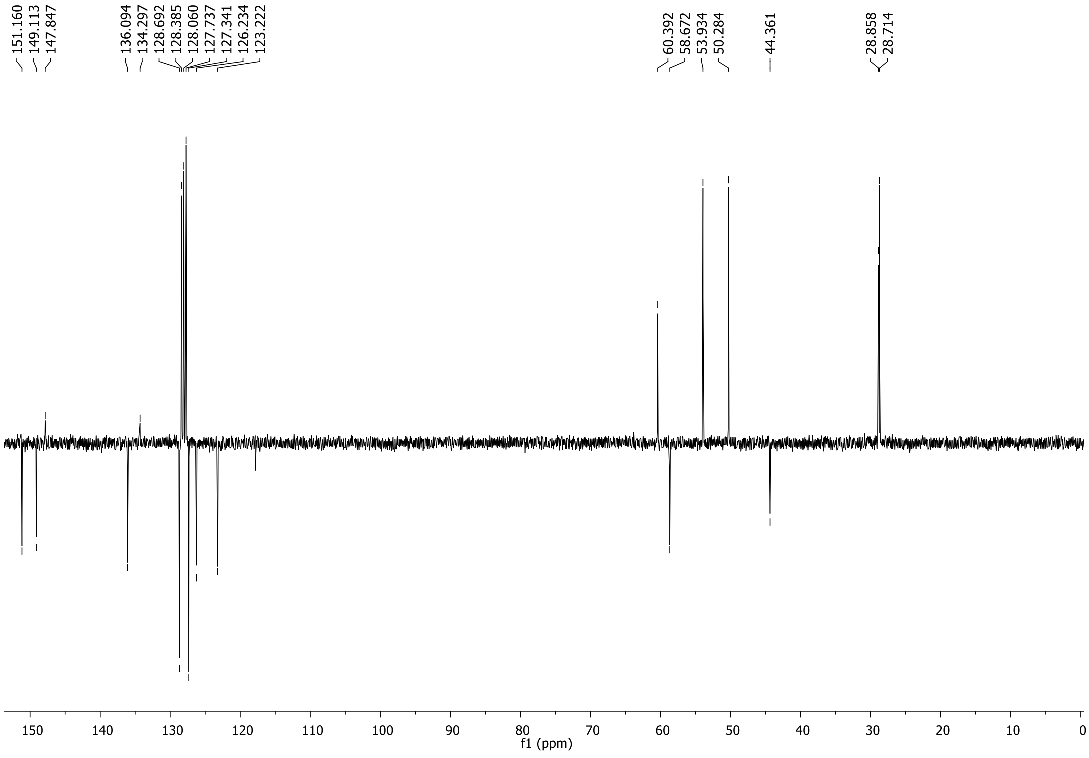

1-((6-methoxypyridin-3-yl)methyl)-4-((1s,4s)-4-phenylcyclohexyl)piperazine (**25**).

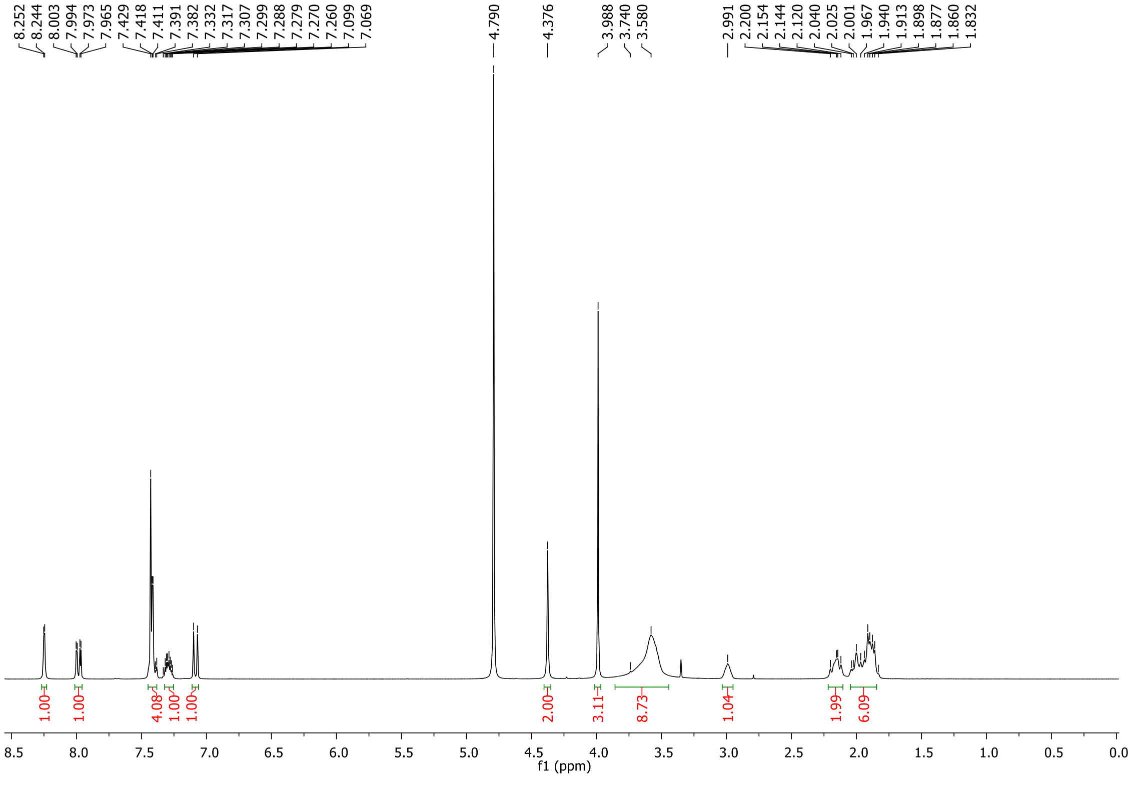

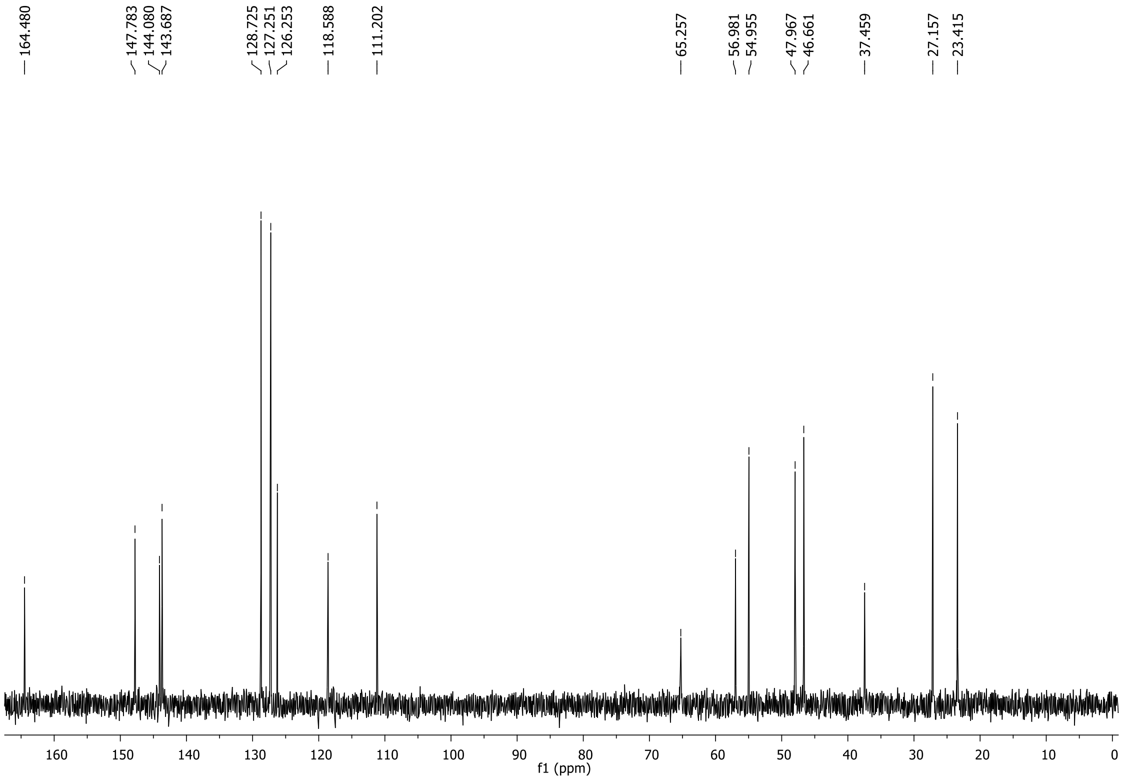
164.480
147.783
144.080
143.687
128.725
127.251
126.253
118.588
111.202
65.257
56.981
54.955
47.967
46.661
37.459
27.157
23.415
160 150 140 130 120 110 100 90 80 70 60 50 40 30 20 10 0
f1 (ppm)

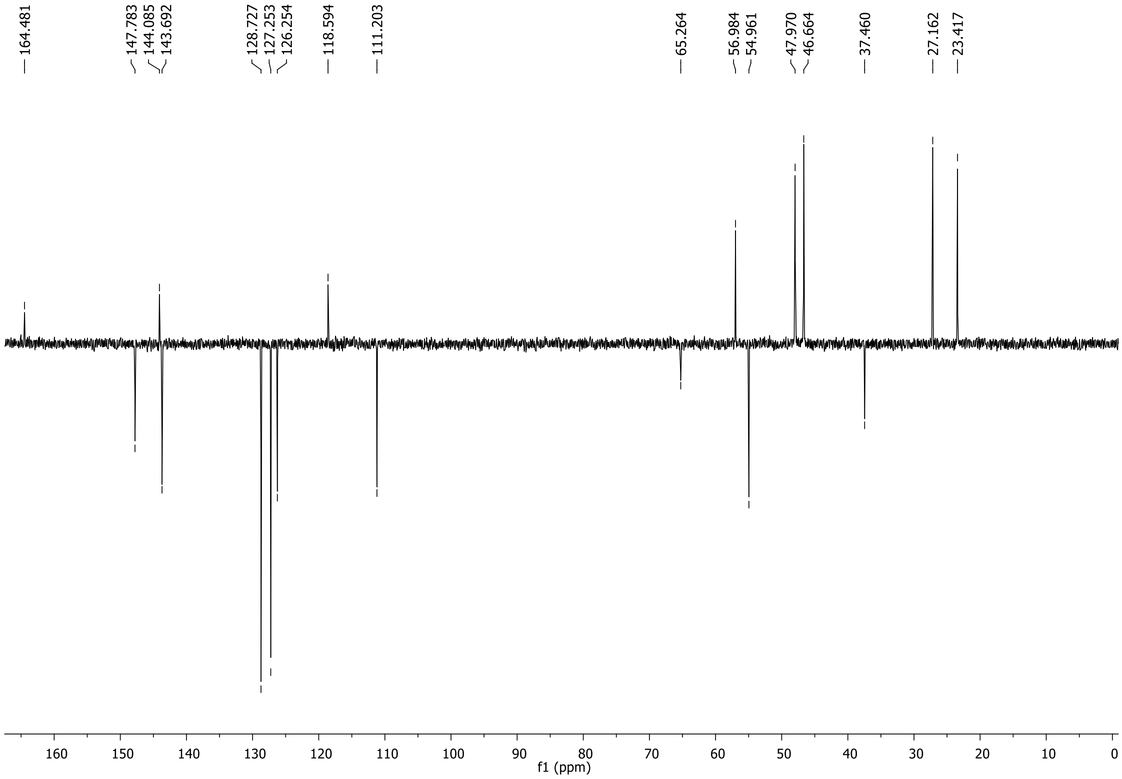
164.481
147.783
144.085
143.692
128.727
127.253
126.254
118.594
111.203
65.264
56.984
54.961
47.970
46.664
37.460
27.162
23.417
160 150 140 130 120 110 100 90 80 70 60 50 40 30 20 10 0
f1 (ppm)

1-((6-bromopyridin-3-yl)methyl)-4-((1s,4s)-4-phenylcyclohexyl)piperazine (**26**).

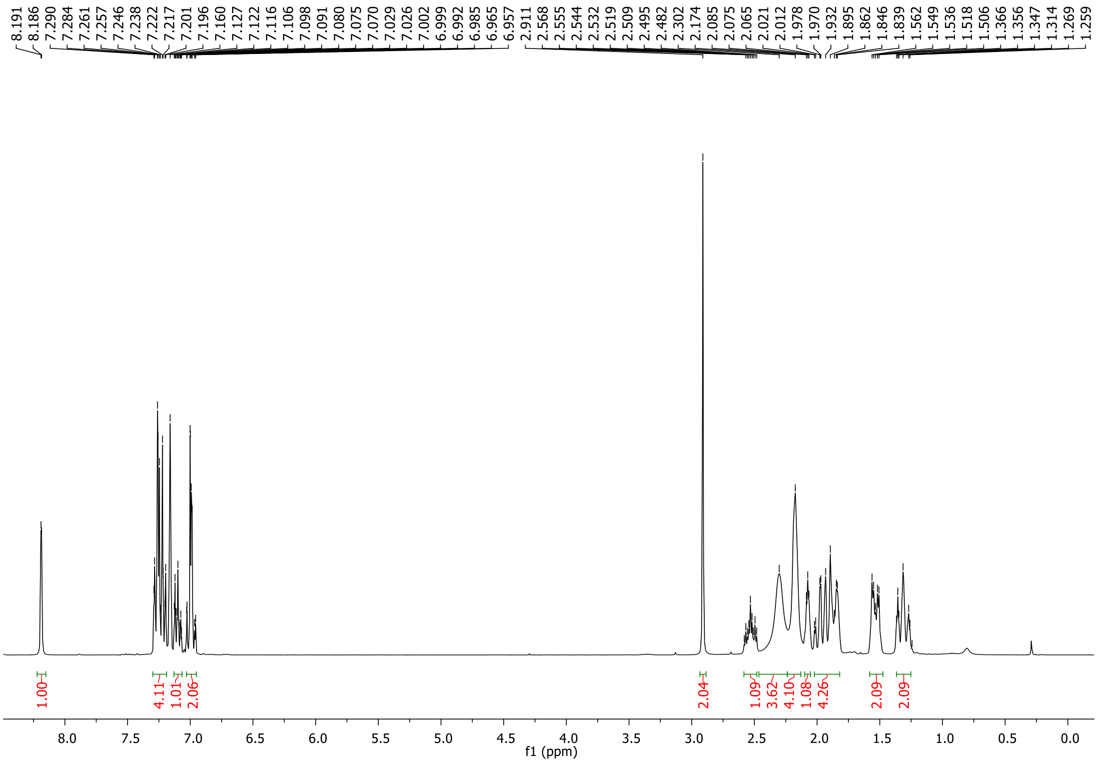

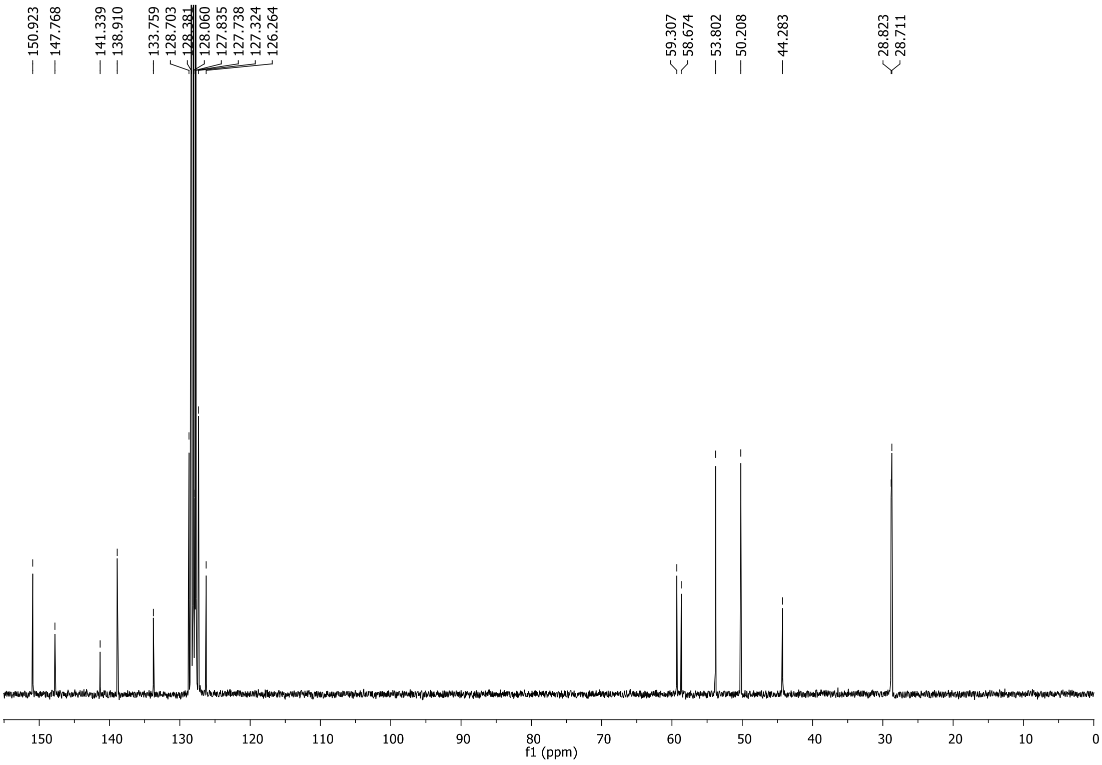

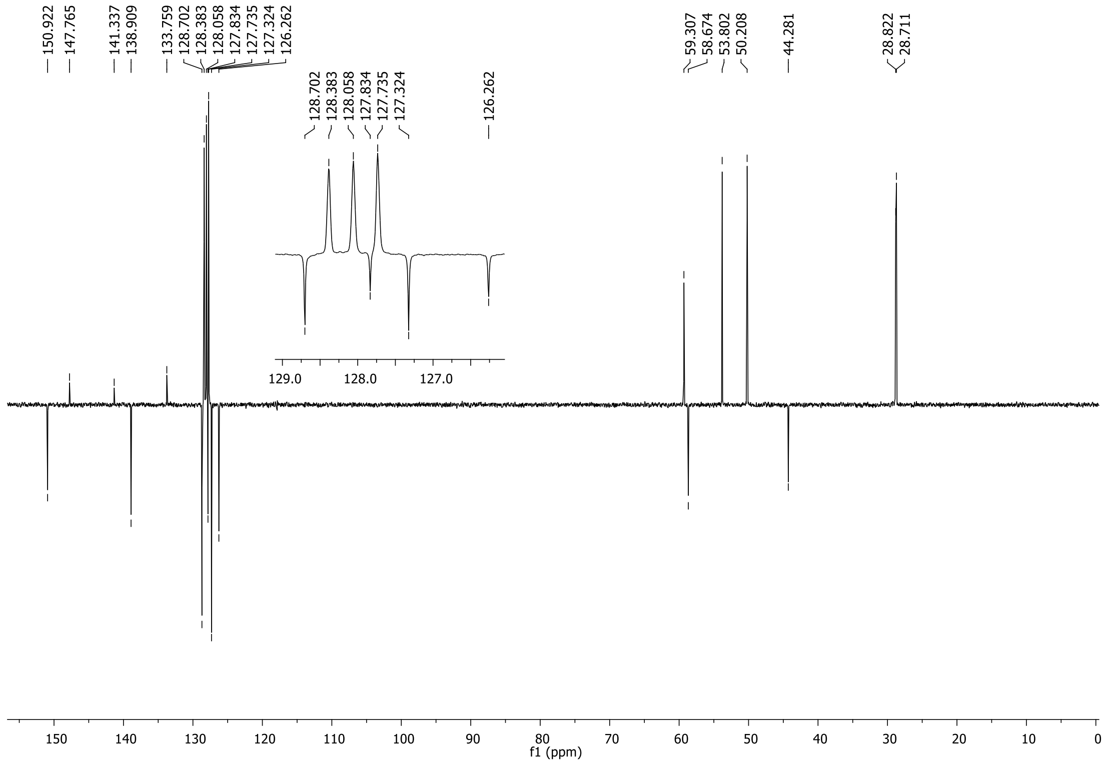

4-(m-tolyl)cyclohexan-1-one (**27**).

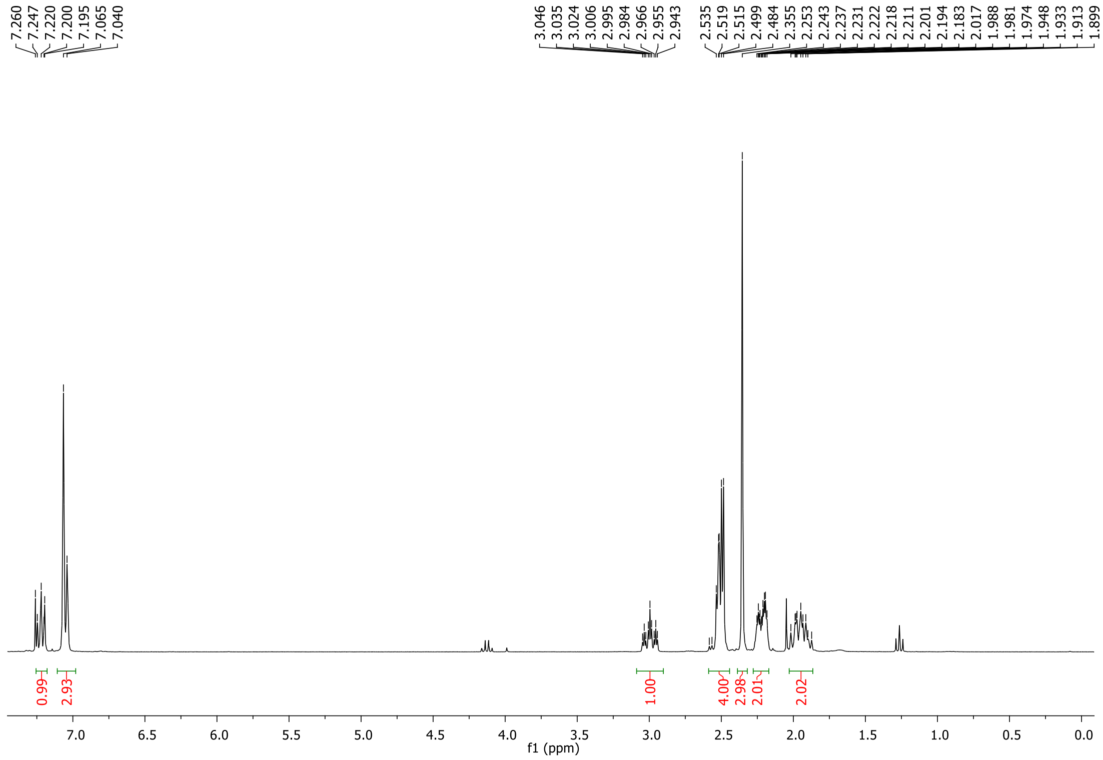

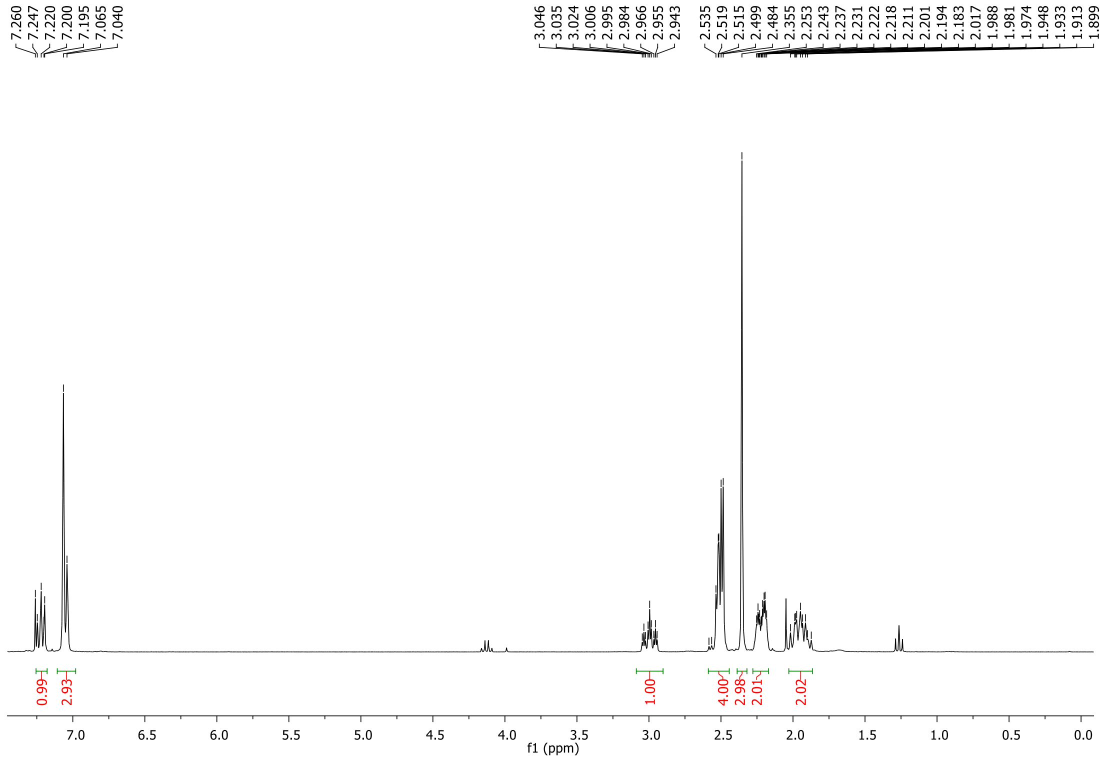

4-(3-(trifluoromethyl)phenyl)cyclohexan-1-one (**28**).

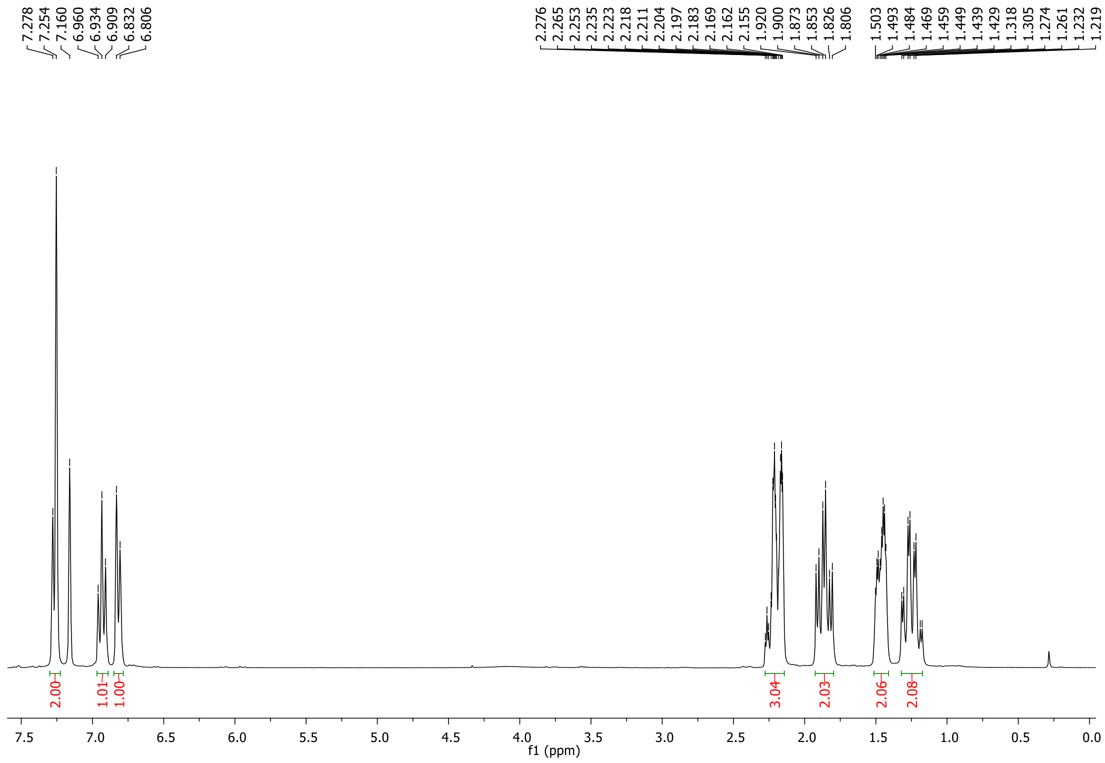

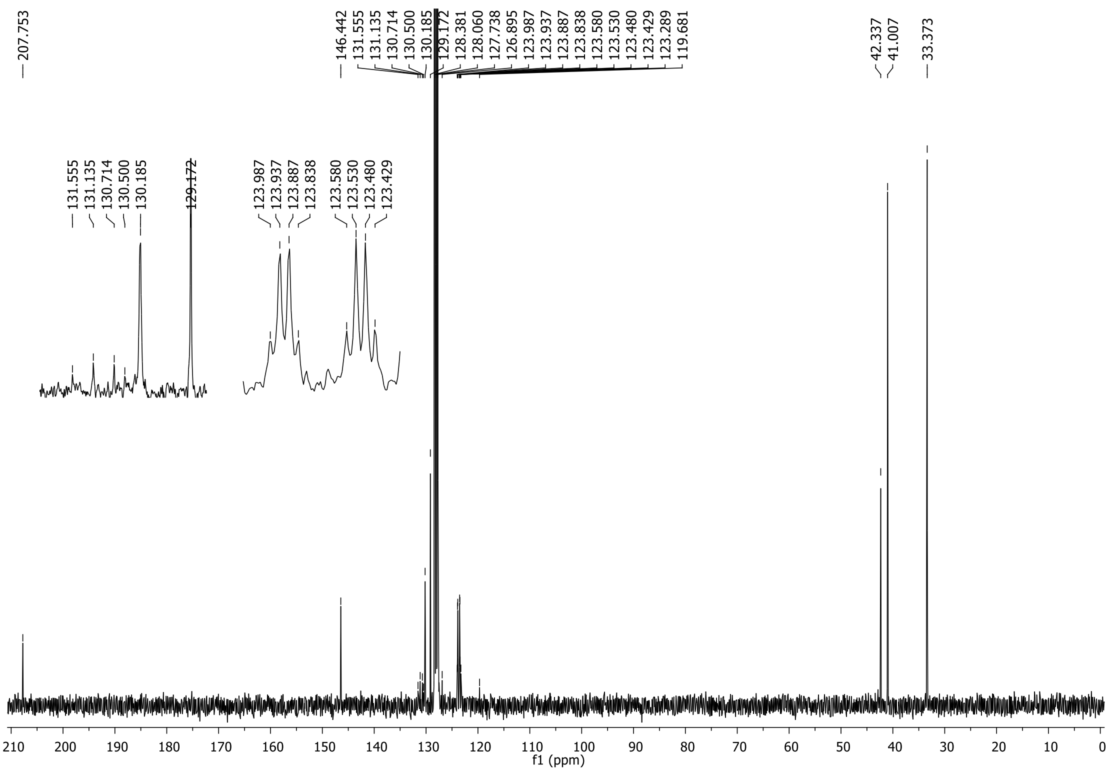

4-(2-(trifluoromethyl)phenyl)cyclohexan-1-one (**29**).

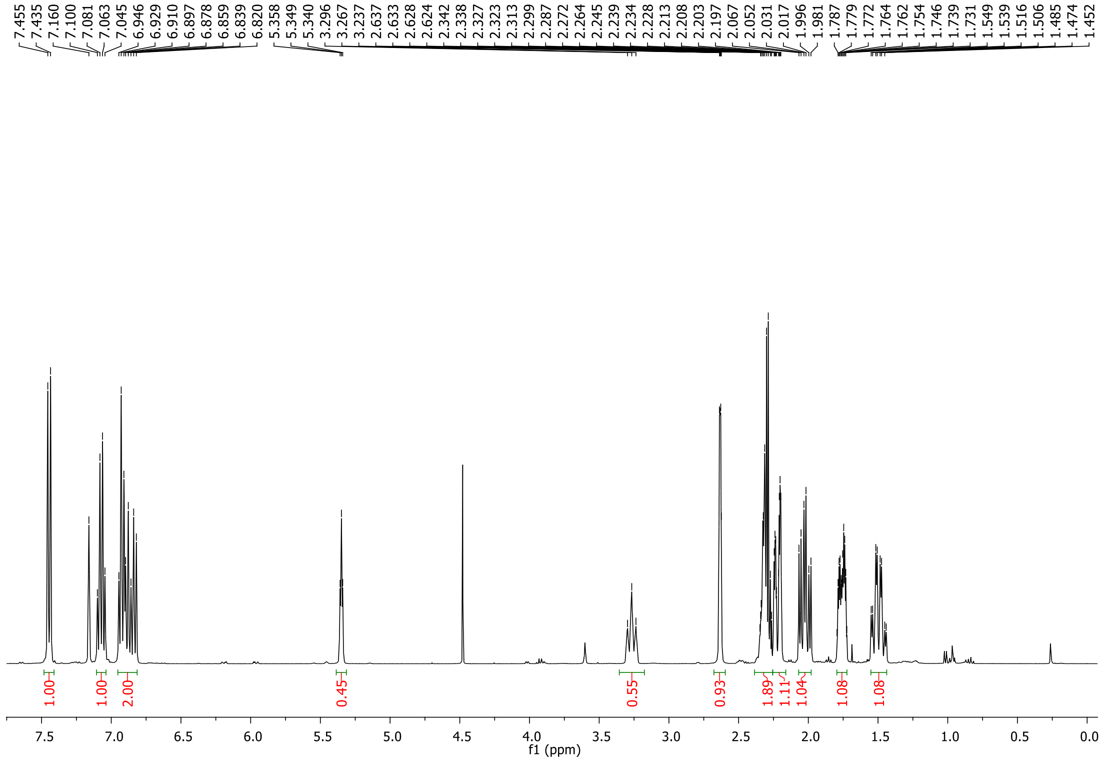

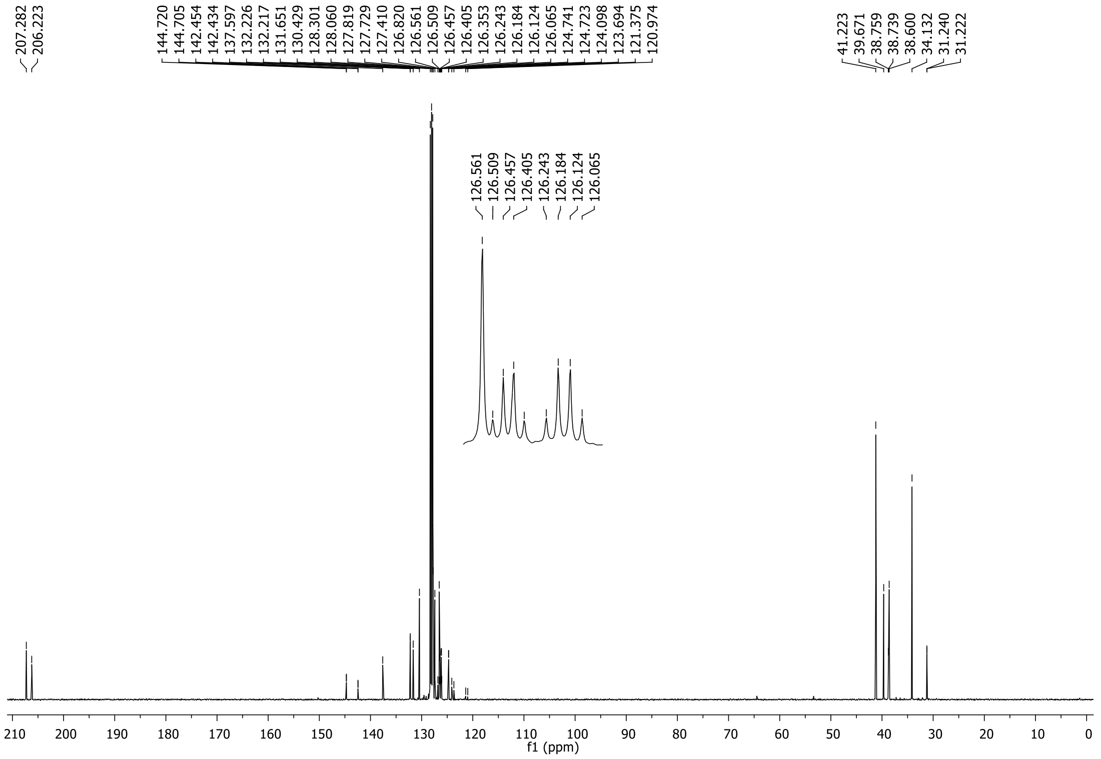

5-((4-((1s,4s)-4-(m-tolyl)cyclohexyl)piperazin-1-yl)methyl)pyridin-2(1H)-one (**30**).

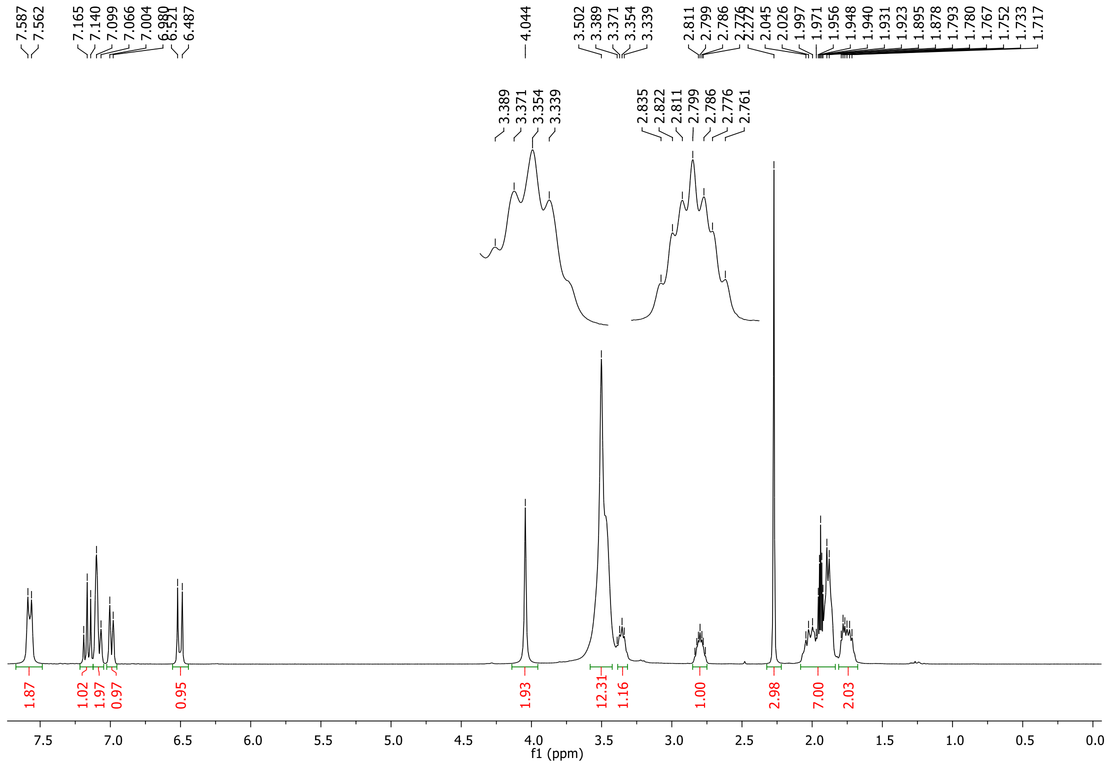

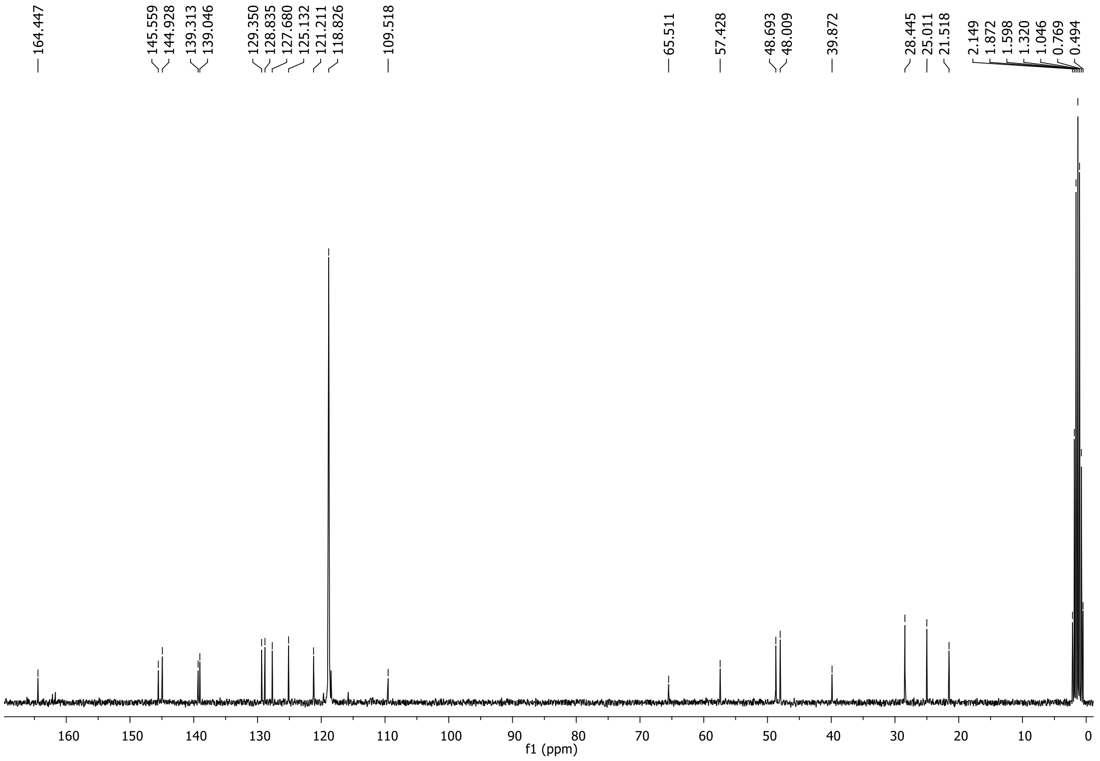
164.447
145.559
144.928
139.313
139.046
129.350
128.835
127.680
125.132
121.211
118.826
109.518
65.511
57.428
48.693
48.009
39.872
28.445
25.011
21.518
2.149
1.872
1.598
1.320
1.046
0.769
0.494
160 150 140 130 120 110 100 90 80 70 60 50 40 30 20 10 0
f1 (ppm)

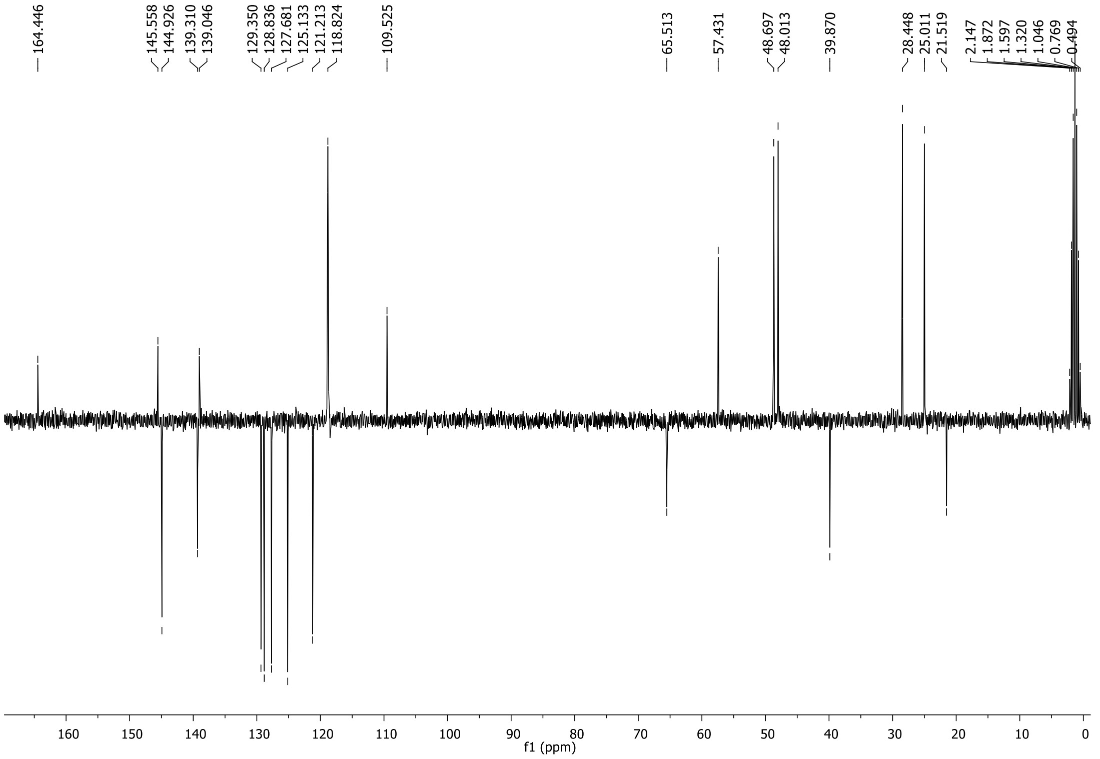
164.446
145.558
144.926
139.310
139.046
129.350
128.836
127.681
125.133
121.213
118.824
109.525
65.513
57.431
48.697
48.013
39.870
28.448
25.011
21.519
2.147
1.872
1.597
1.320
1.046
0.769
0.494
160 150 140 130 120 110 100 90 80 70 60 50 40 30 20 10 0
f1 (ppm)

5-((4-((1s,4s)-4-(3-(trifluoromethyl)phenyl)cyclohexyl)piperazin-1-yl)methyl)pyridin-2(1H)-one (**31**).

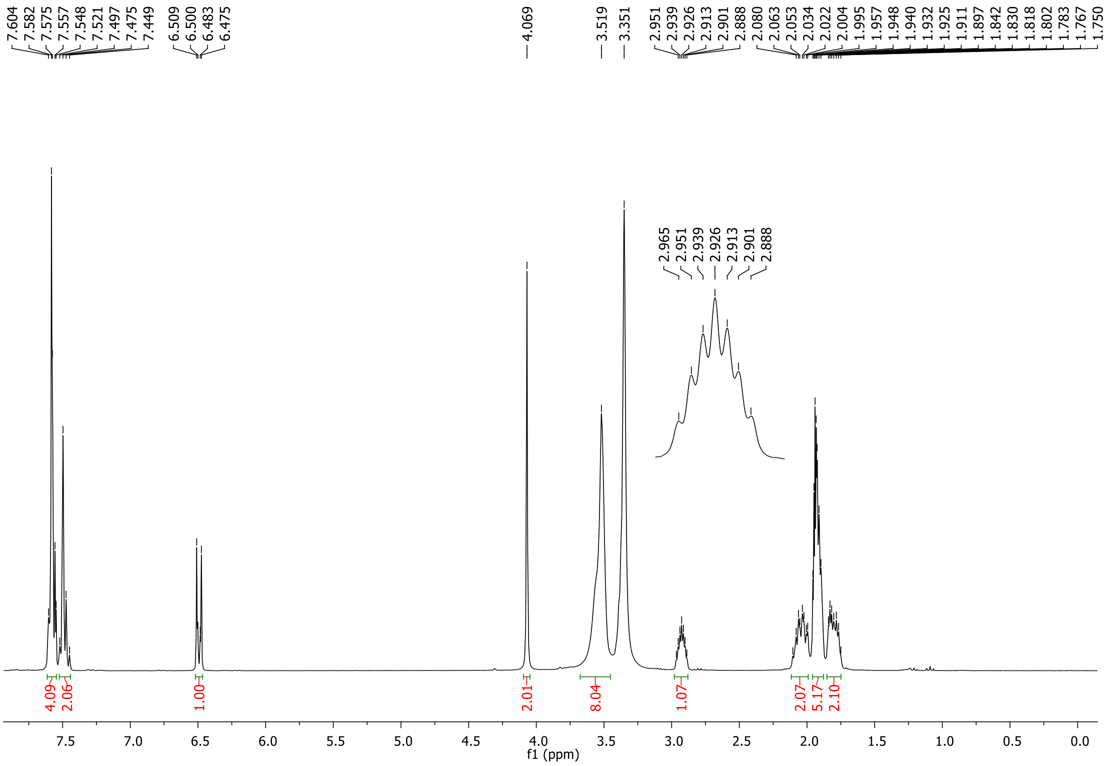

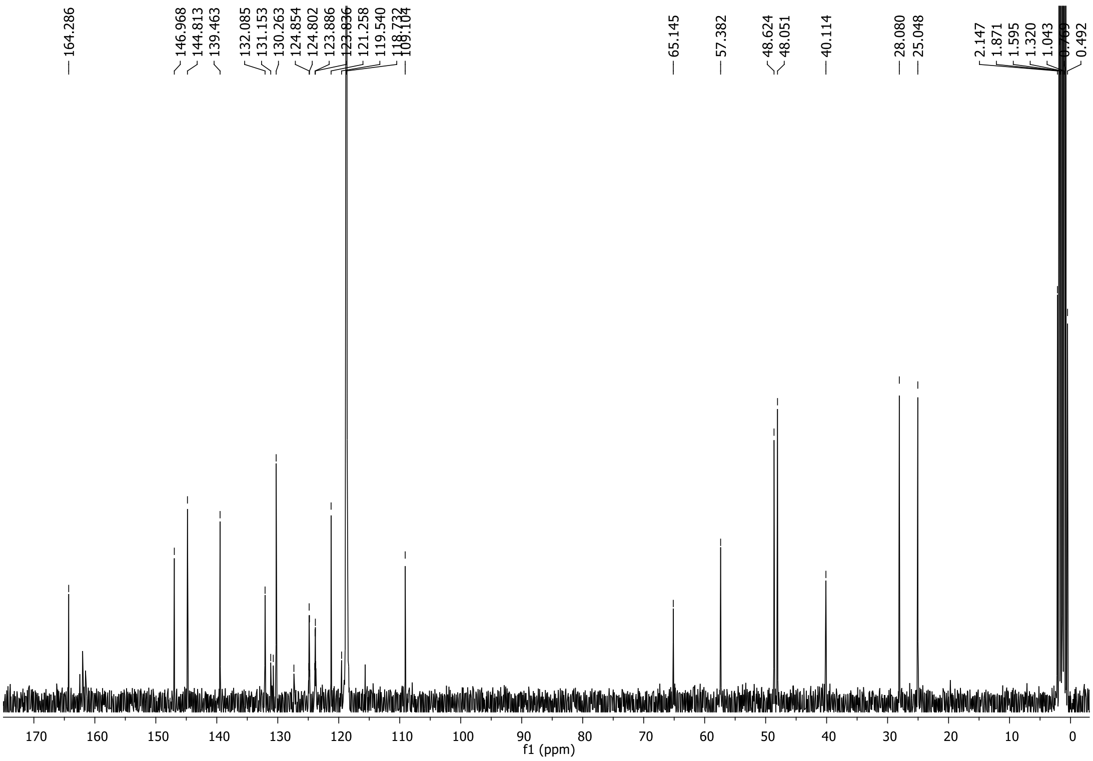

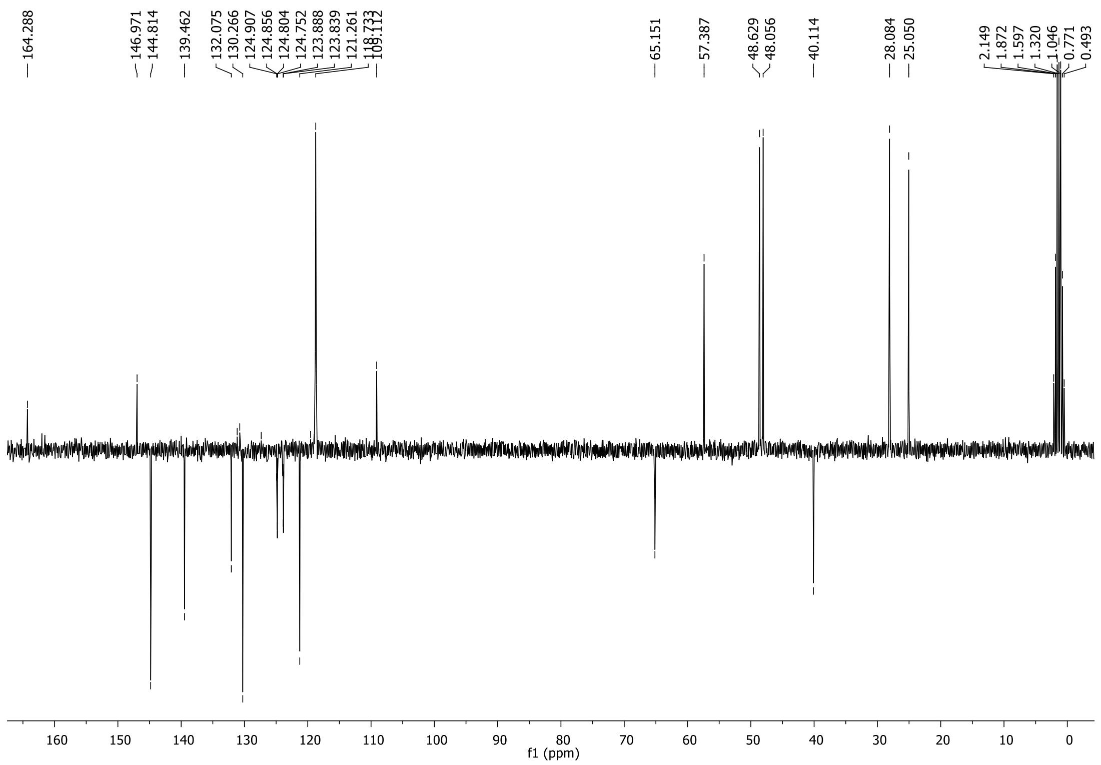

5-((4-((1s,4s)-4-(2-(trifluoromethyl)phenyl)cyclohexyl)piperazin-1-yl)methyl)pyridin-2(1H)-one (**32**).

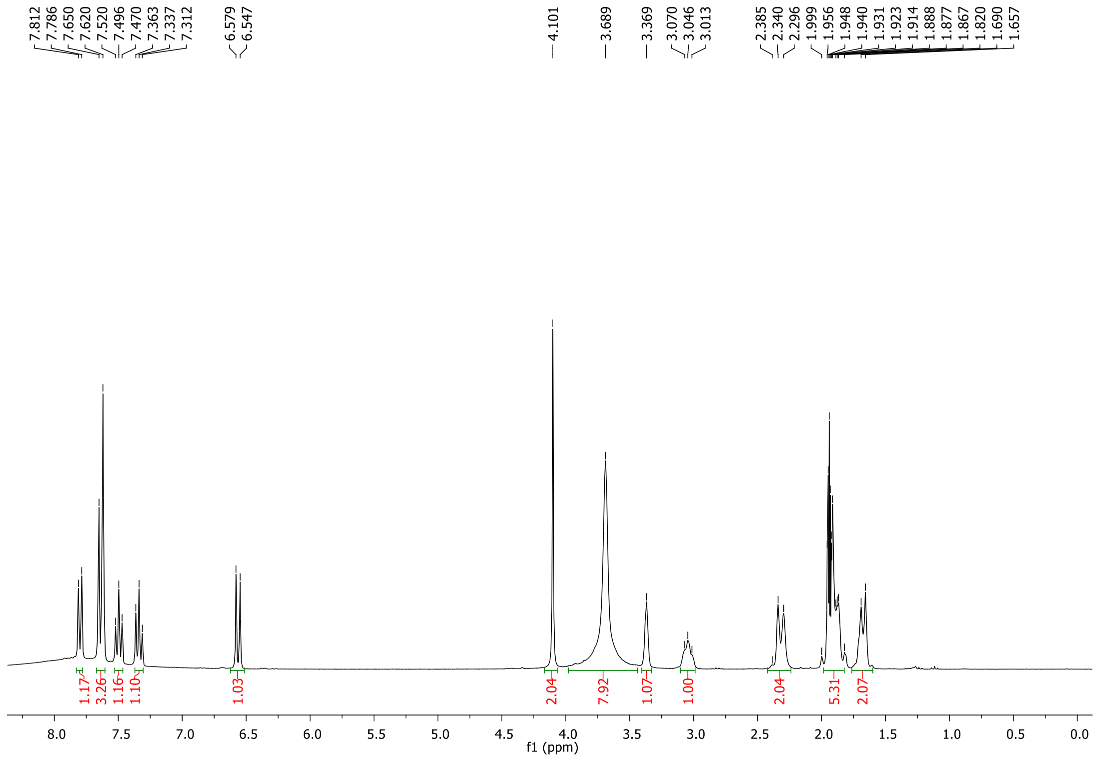

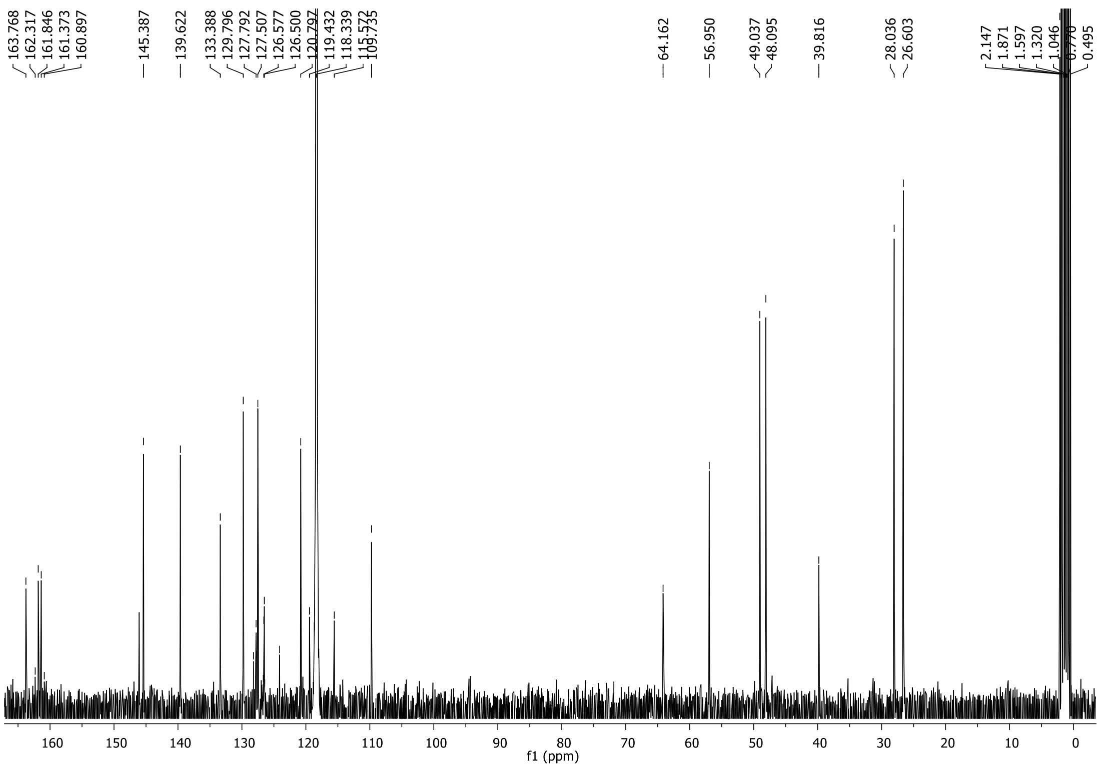
163.768
162.317
161.846
161.373
160.897
145.387
139.622
133.388
129.796
127.792
127.507
126.577
126.500
120.797
119.432
118.339
115.572
109.735
64.162
56.950
49.037
48.095
39.816
28.036
26.603
2.147
1.871
1.597
1.320
1.046
0.770
0.495
160 150 140 130 120 110 100 90 80 70 60 50 40 30 20 10 0
f1 (ppm)

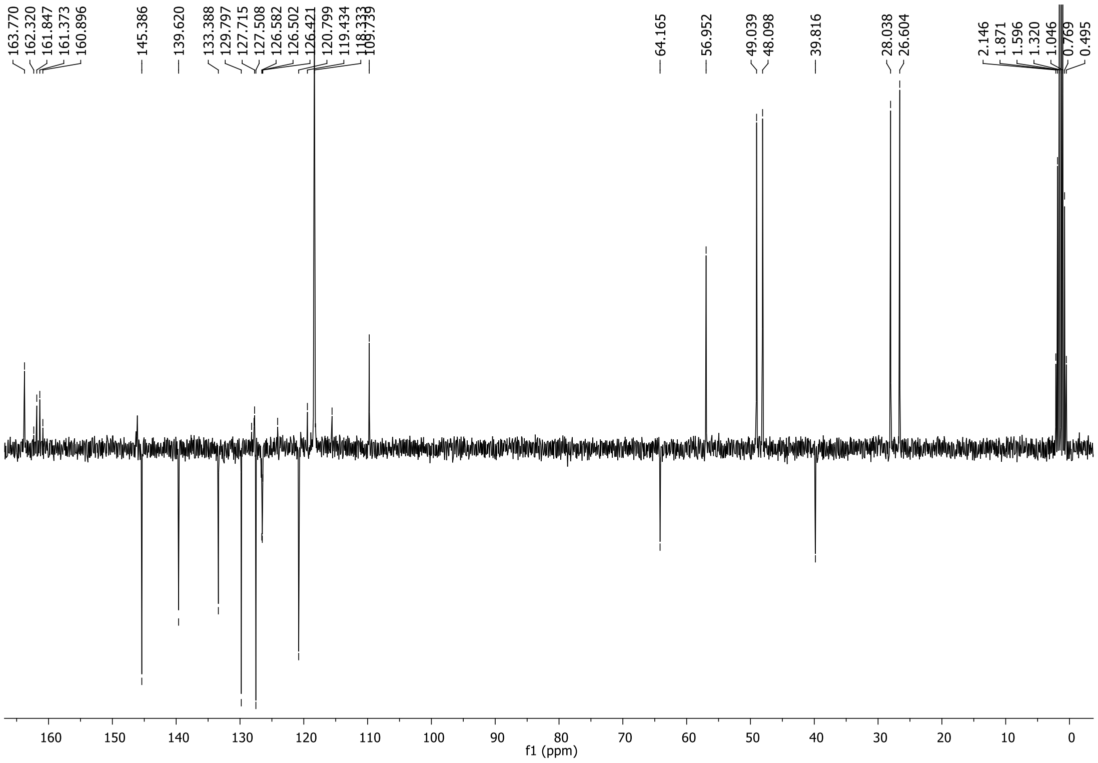
163.770
162.320
161.847
161.373
160.896
145.386
139.620
133.388
129.797
127.715
127.508
126.582
126.502
126.421
120.799
119.434
118.333
109.739
64.165
56.952
49.039
48.098
39.816
28.038
26.604
2.146
1.871
1.596
1.320
1.046
0.769
0.495
160 150 140 130 120 110 100 90 80 70 60 50 40 30 20 10 0
f1 (ppm)

4-hydroxy-4-(3-(trifluoromethyl)phenyl)cyclohexan-1-one (**37**).

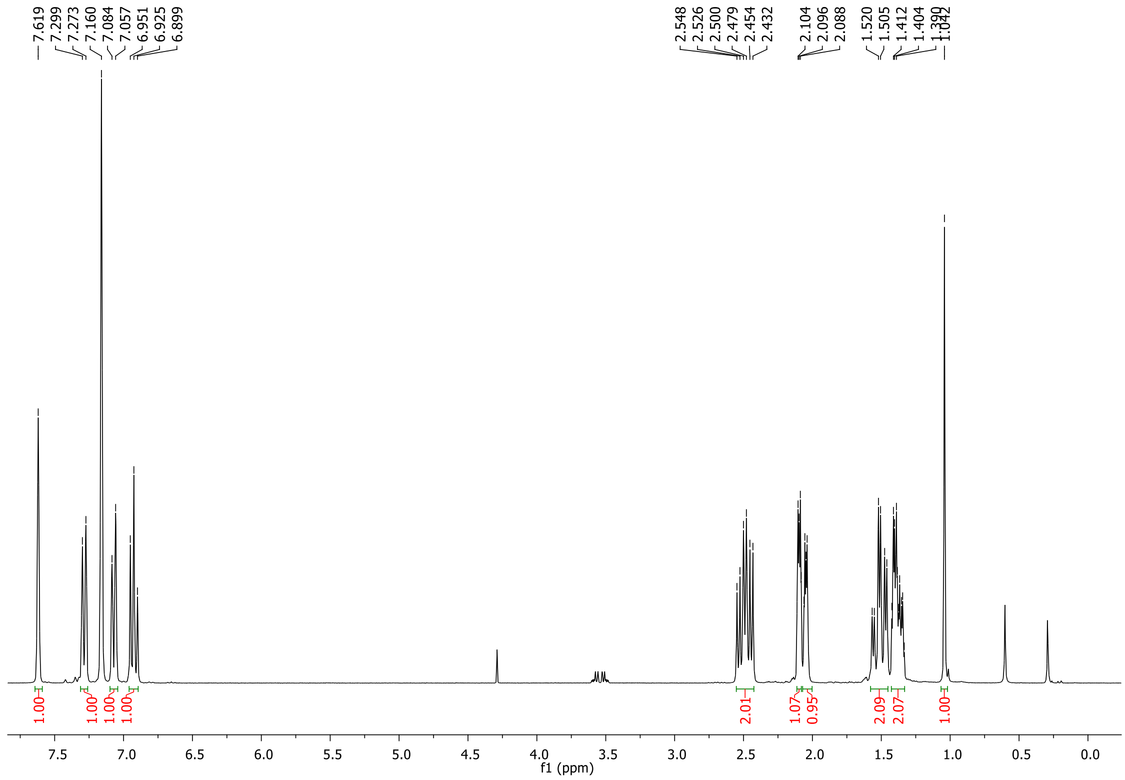

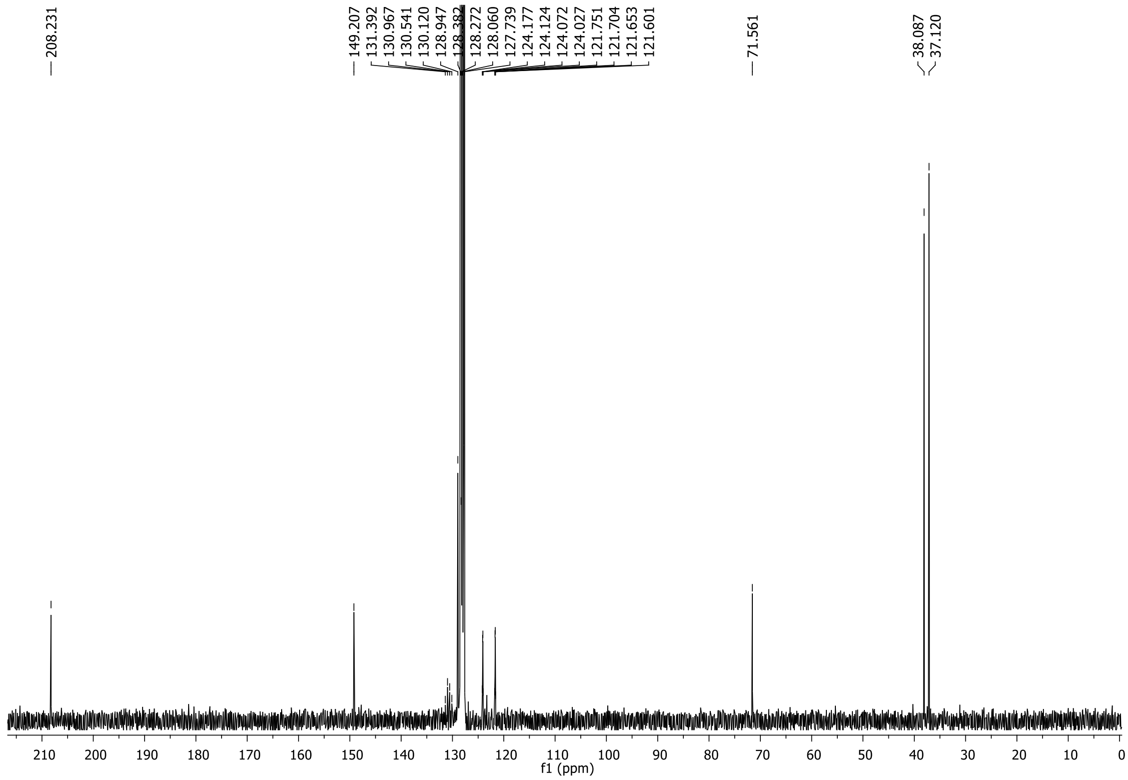

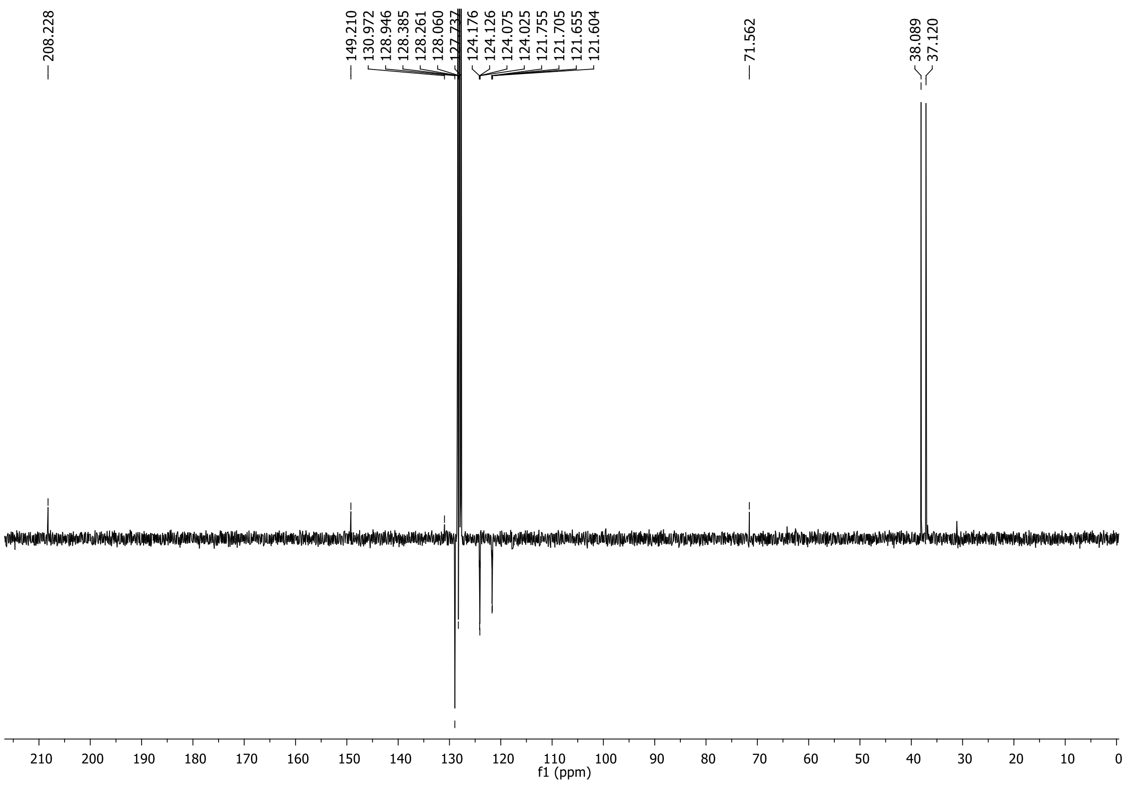
208.228
149.210
130.972
128.946
128.385
128.261
128.060
127.737
124.176
124.126
124.075
124.025
121.755
121.705
121.655
121.604
71.562
38.089
37.120
210
200
190
180
170
160
150
140
130
120
110
100
90
80
70
60
50
40
30
20
10
0
f1 (ppm)

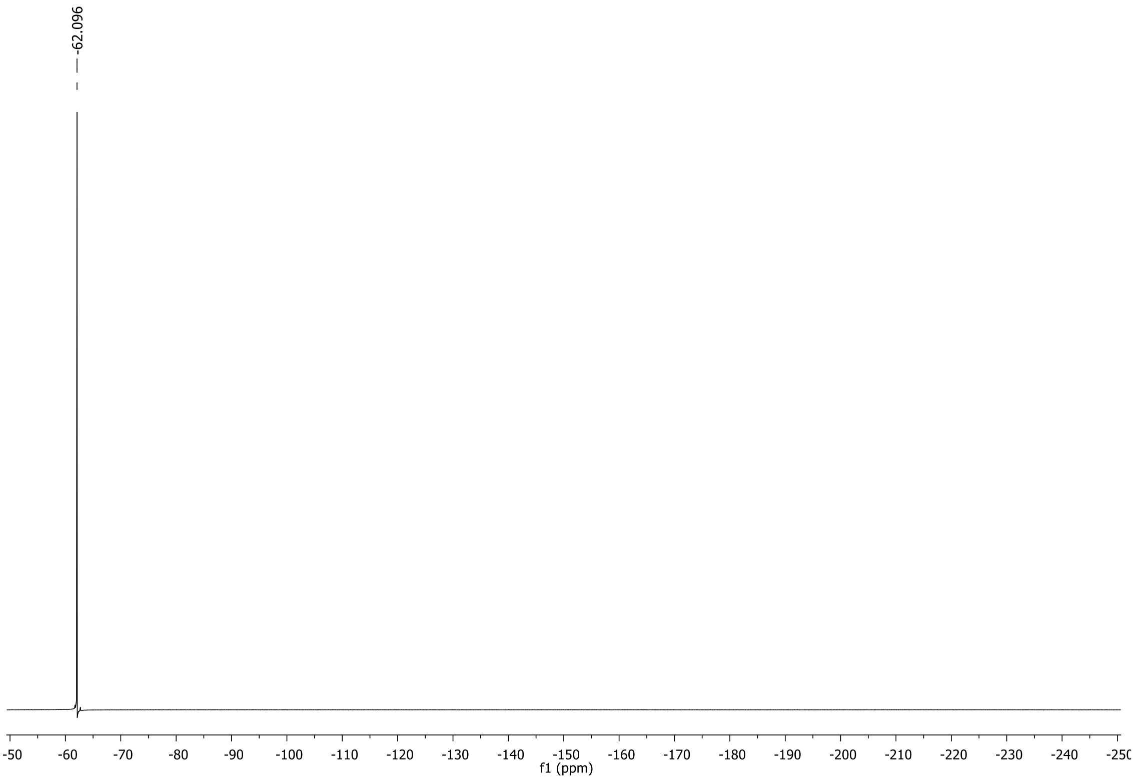
-62.096
-50
-60
-70
-80
-90
-100
-110
-120
-130
-140
-150
-160
-170
-180
-190
-200
-210
-220
-230
-240
-250
f1 (ppm)

4-hydroxy-4-(2-(trifluoromethyl)phenyl)cyclohexan-1-one (**38**).

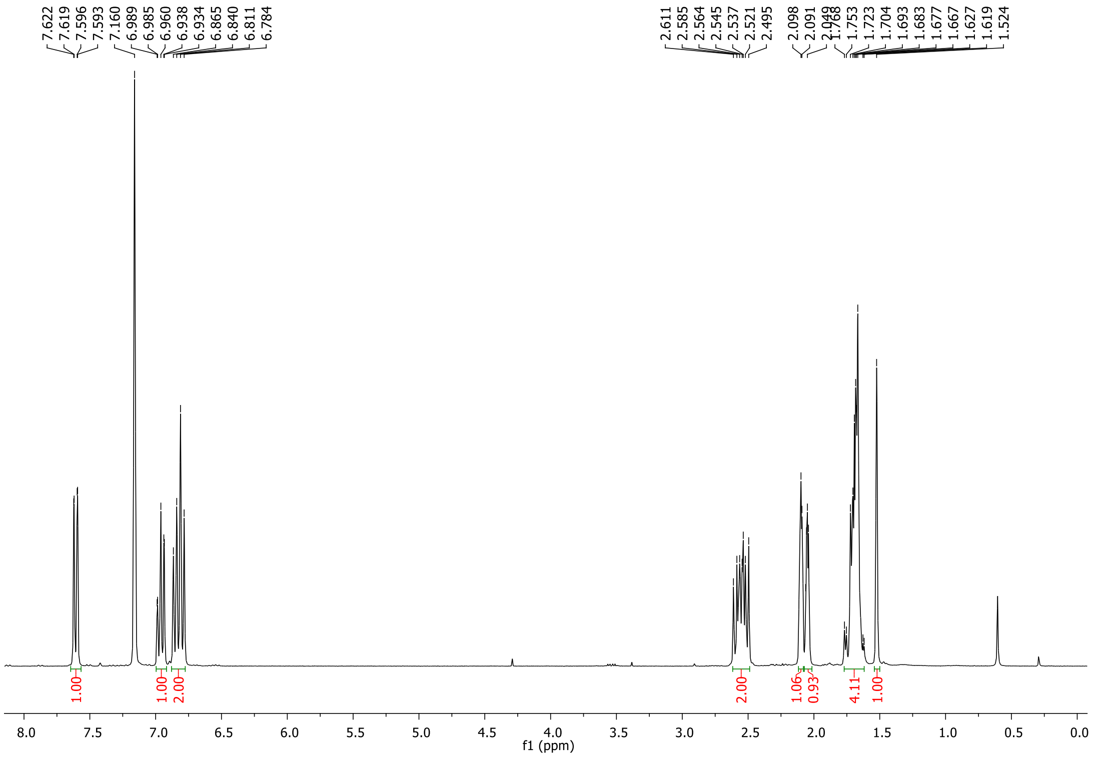

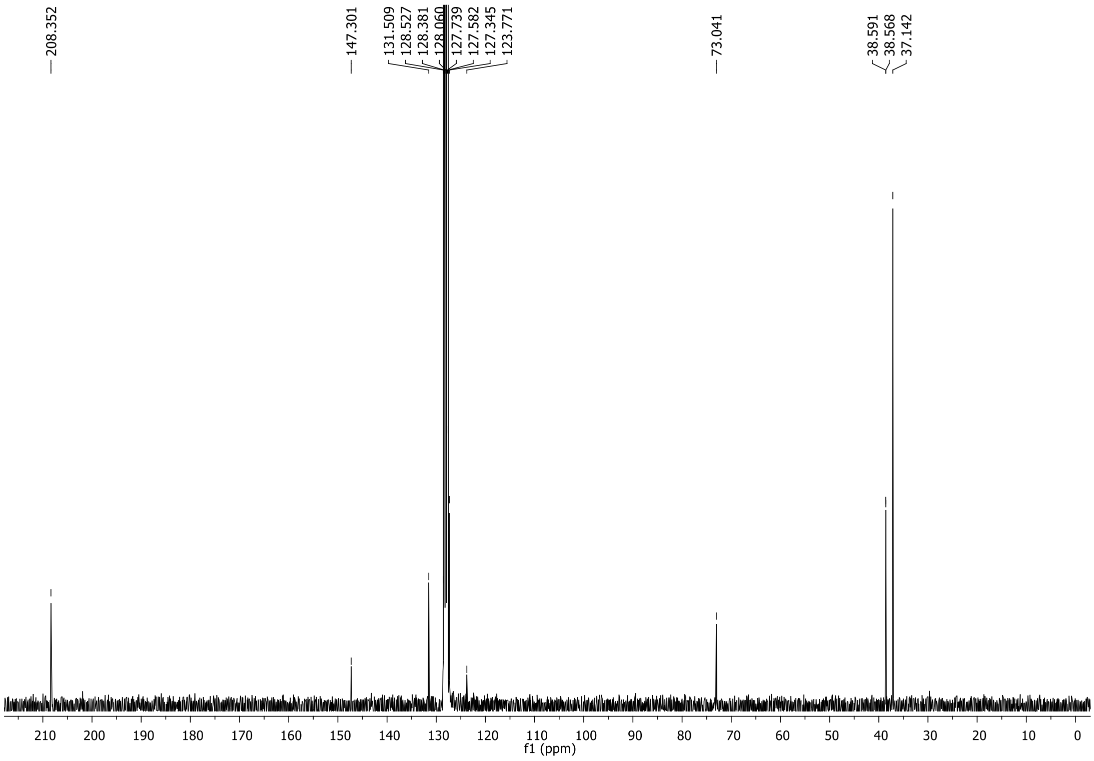

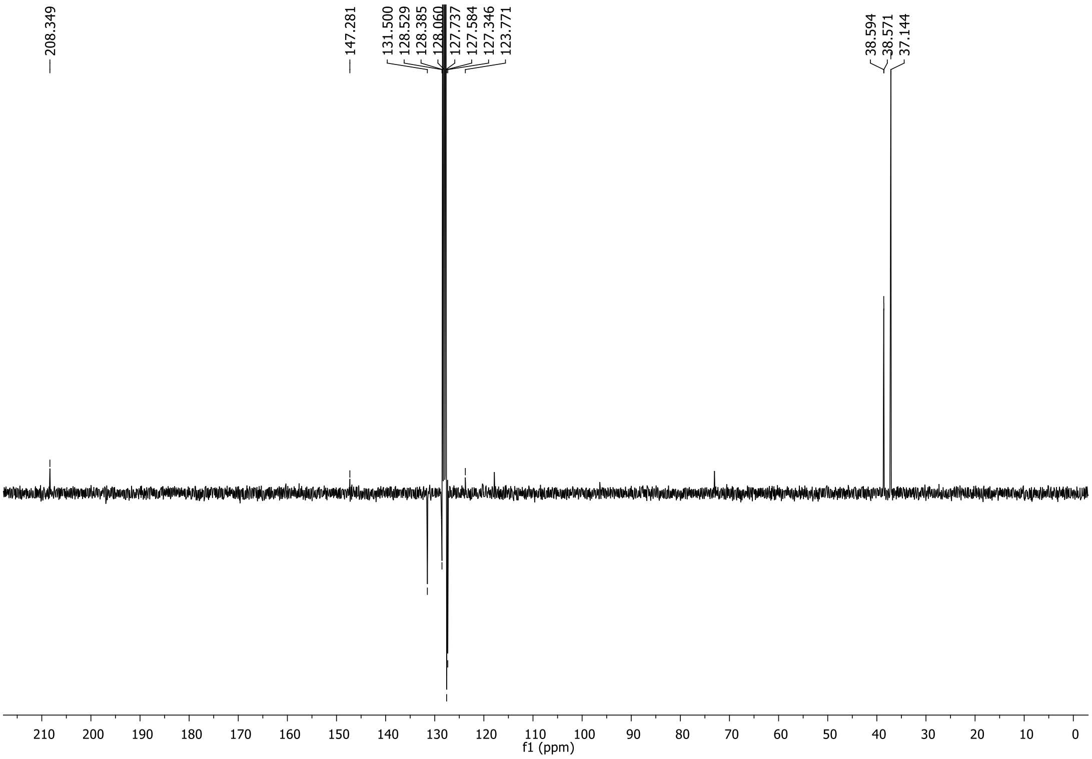
208.349
147.281
131.500
128.529
128.385
128.060
127.737
127.584
127.346
123.771
38.594
38.571
37.144
210 200 190 180 170 160 150 140 130 120 110 100 90 80 70 60 50 40 30 20 10 0
f1 (ppm)

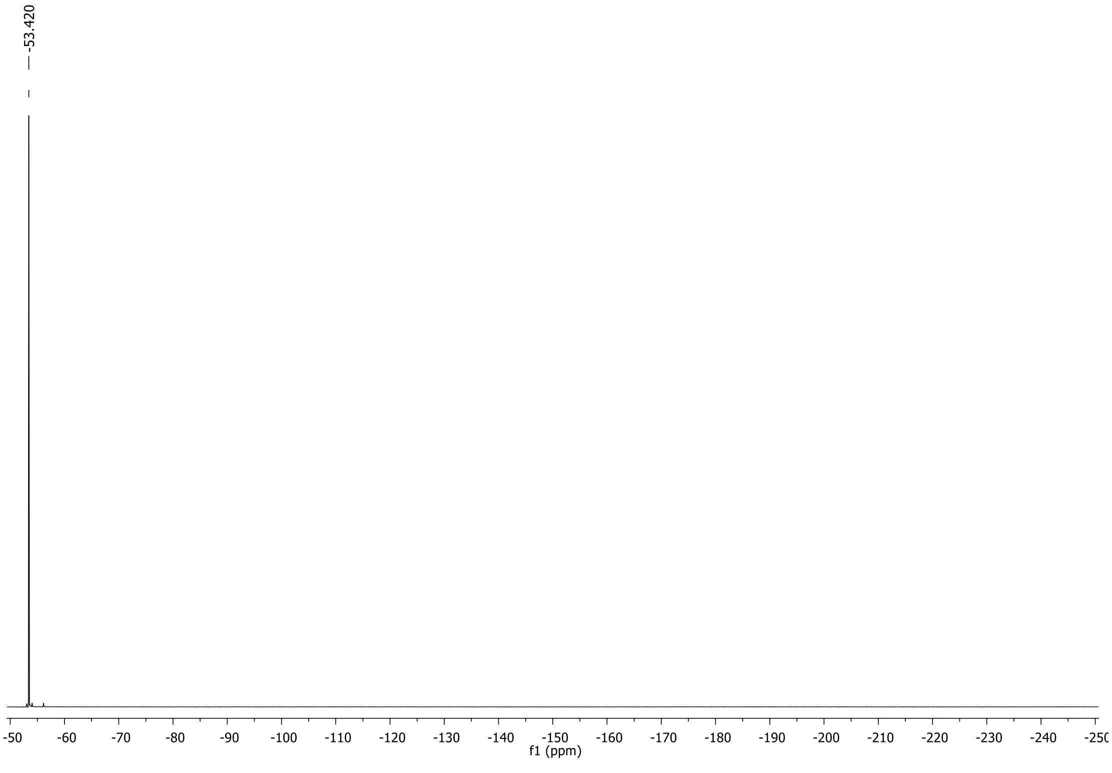
-53.420
-50 -60 -70 -80 -90 -100 -110 -120 -130 -140 -150 -160 -170 -180 -190 -200 -210 -220 -230 -240 -250
f1 (ppm)

5-((4-((1r,4r)-4-hydroxy-4-(3-(trifluoromethyl)phenyl)cyclohexyl)piperazin-1-yl)methyl)pyridin-2(1H)-one (**39,** 3OG).

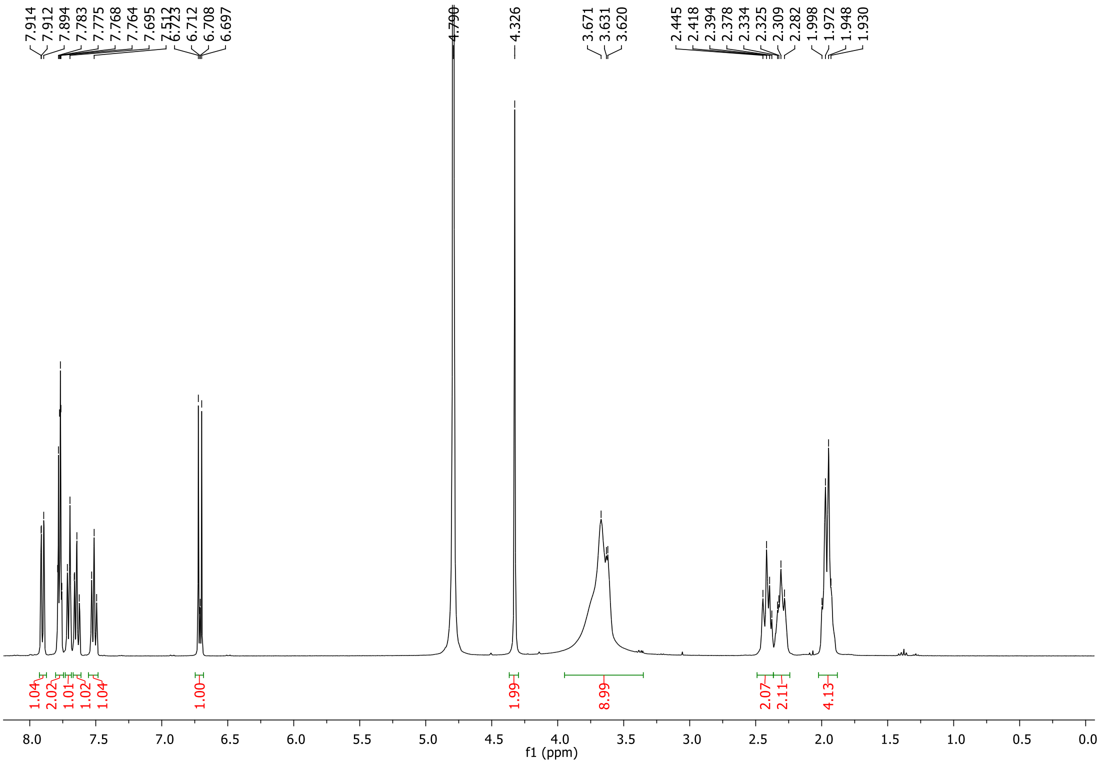

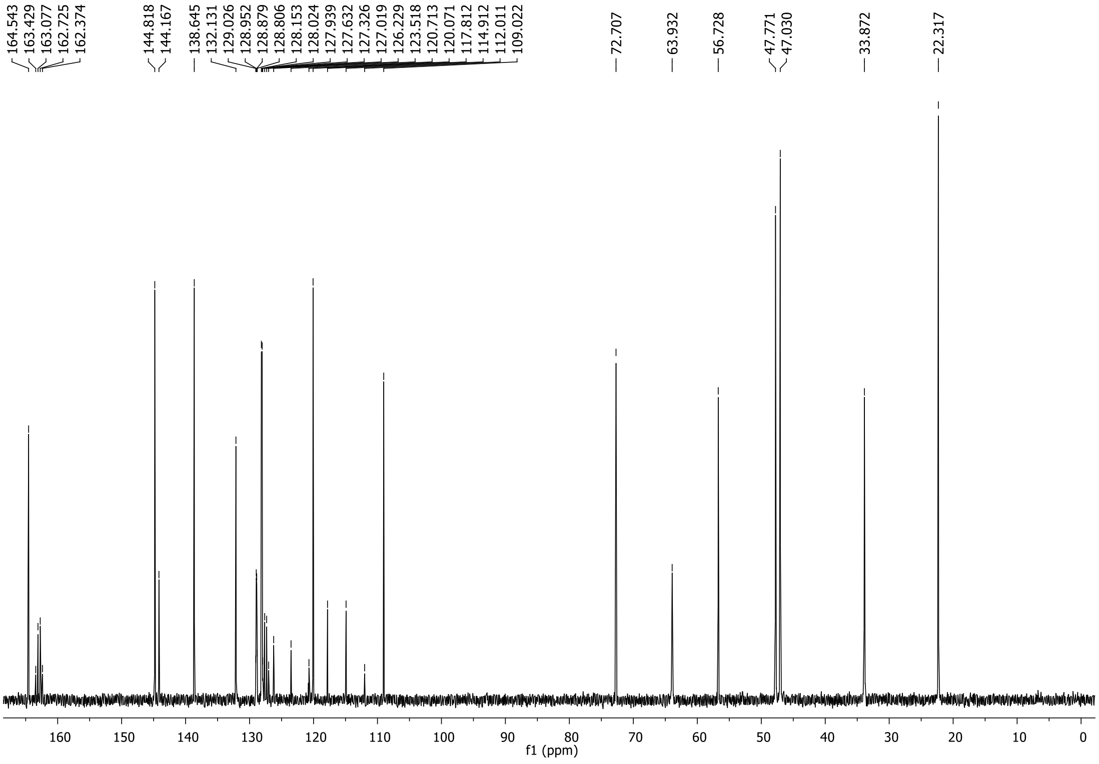

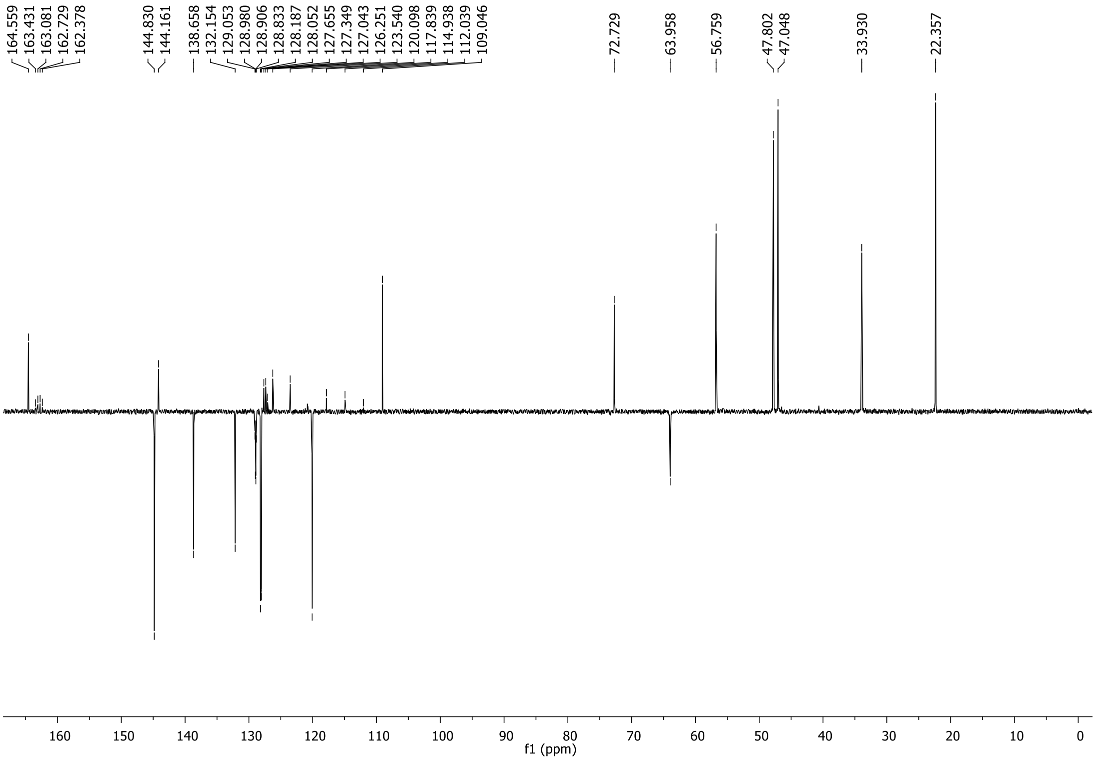
164.559
163.431
163.081
162.729
162.378
144.830
144.161
138.658
132.154
129.053
128.980
128.906
128.833
128.187
128.052
127.655
127.349
127.043
126.251
123.540
120.098
117.839
114.938
112.039
109.046
72.729
63.958
56.759
47.802
47.048
33.930
22.357
160 150 140 130 120 110 100 90 80 70 60 50 40 30 20 10 0
f1 (ppm)

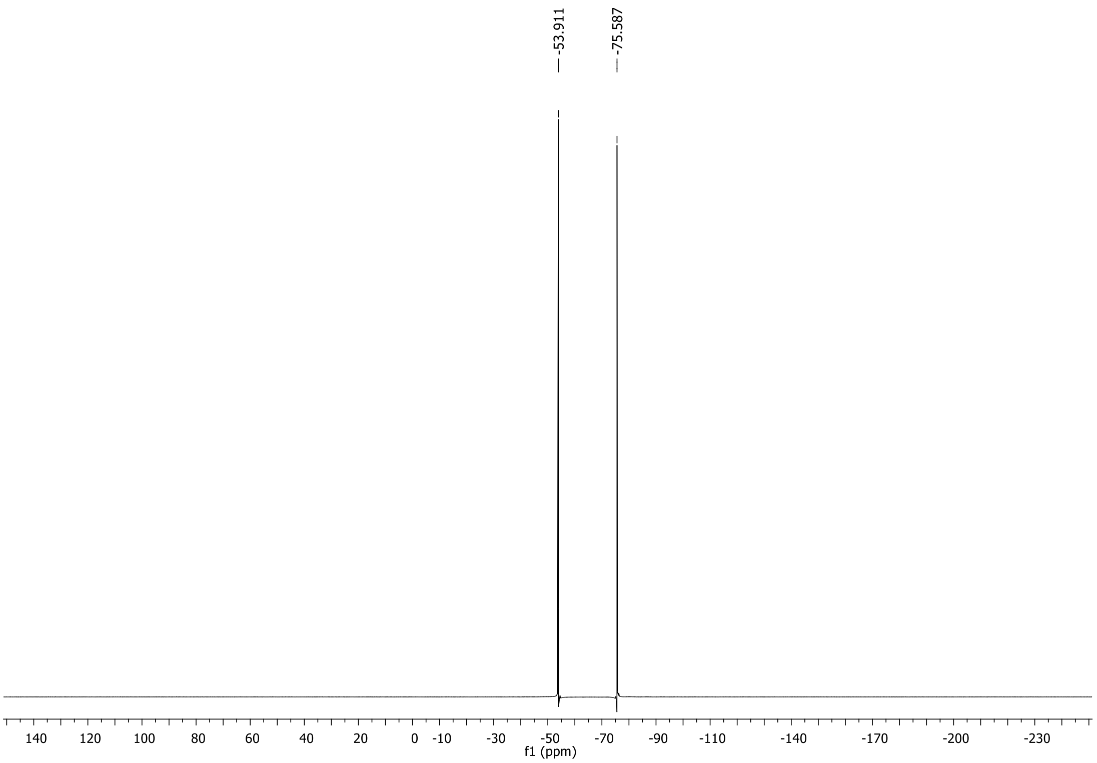
-53.911
-75.587
140 120 100 80 60 40 20 0 -10 -30 -50 -70 -90 -110 -140 -170 -200 -230
f1 (ppm)

5-((4-((1r,4r)-4-hydroxy-4-(2-(trifluoromethyl)phenyl)cyclohexyl)piperazin-1-yl)methyl)pyridin-2(1H)-one (**40**).

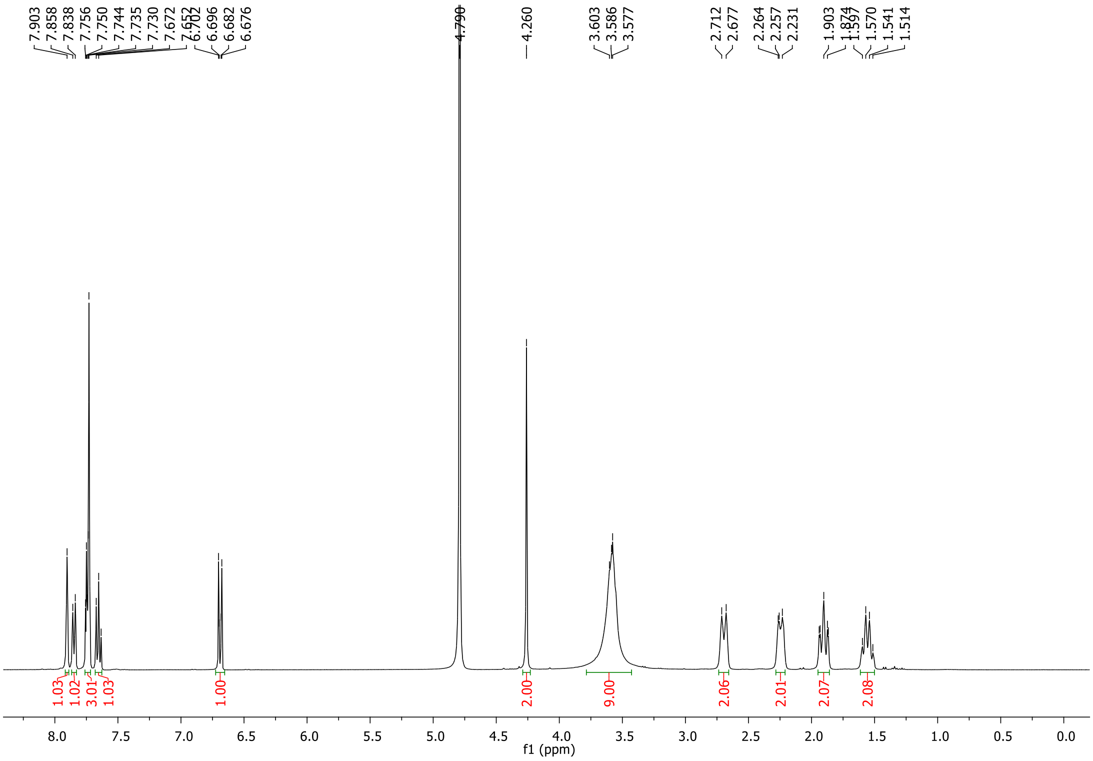

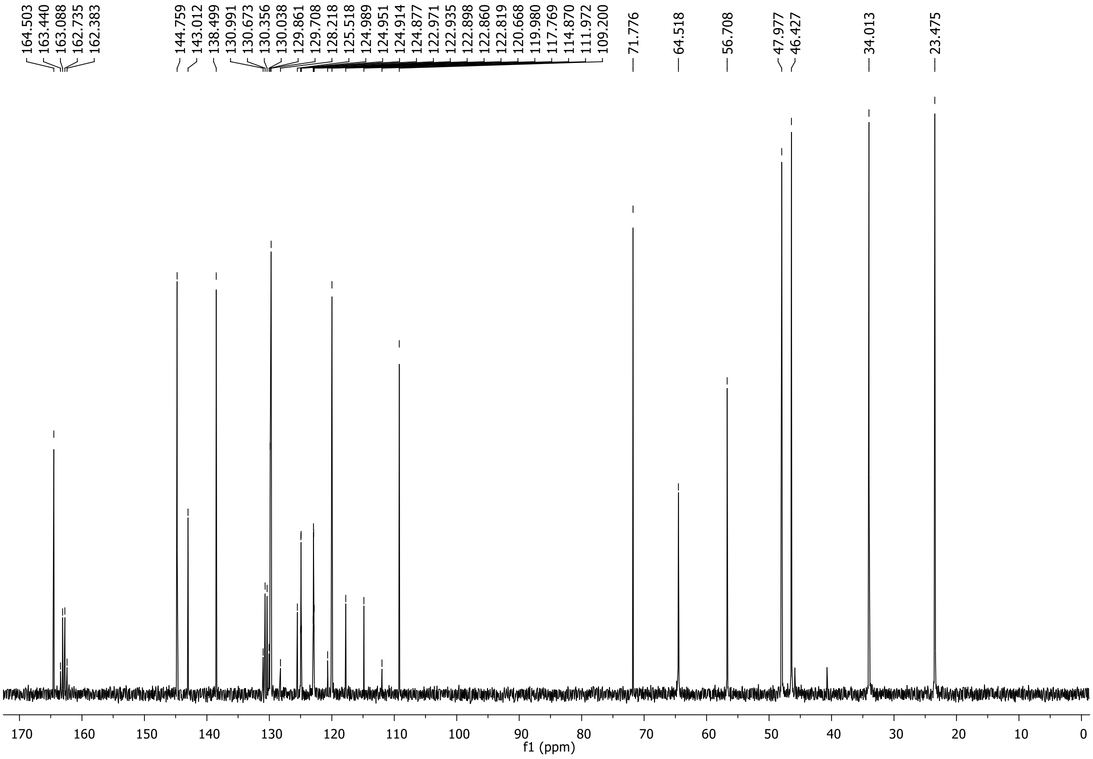

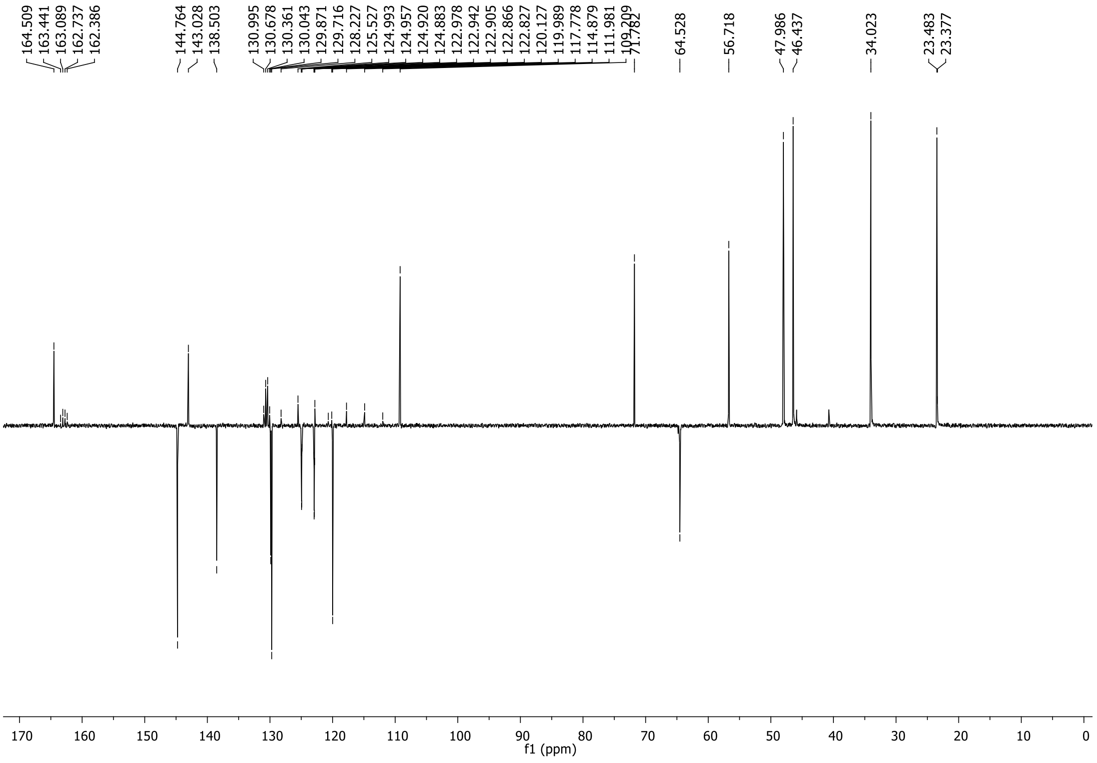
164.509
163.441
163.089
162.737
162.386
144.764
143.028
138.503
130.995
130.678
130.361
130.043
129.871
129.716
128.227
125.527
124.993
124.957
124.920
124.883
122.978
122.942
122.905
122.866
122.827
120.127
119.989
117.778
114.879
111.981
109.209
71.782
64.528
56.718
47.986
46.437
34.023
23.483
23.377
f1 (ppm)

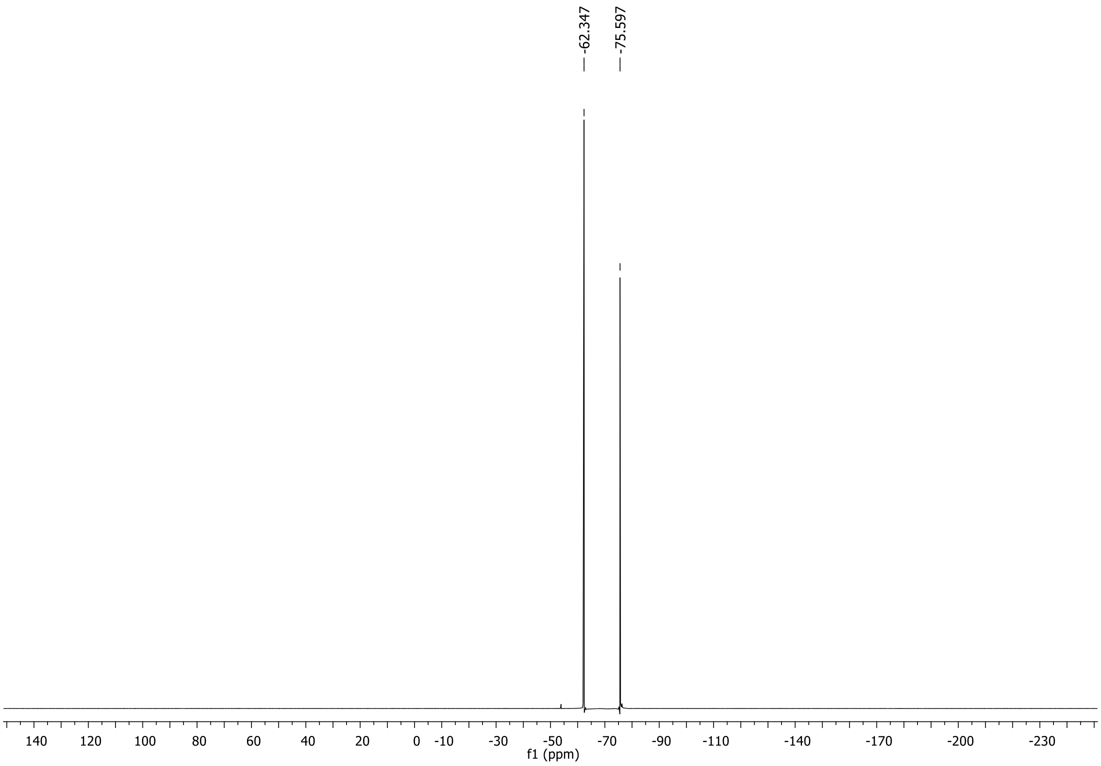
-62.347
-75.597
f1 (ppm)

(E)-N'-(4-hydroxy-3-methoxybenzylidene)hexane-1-sulfonohydrazide (**41**).

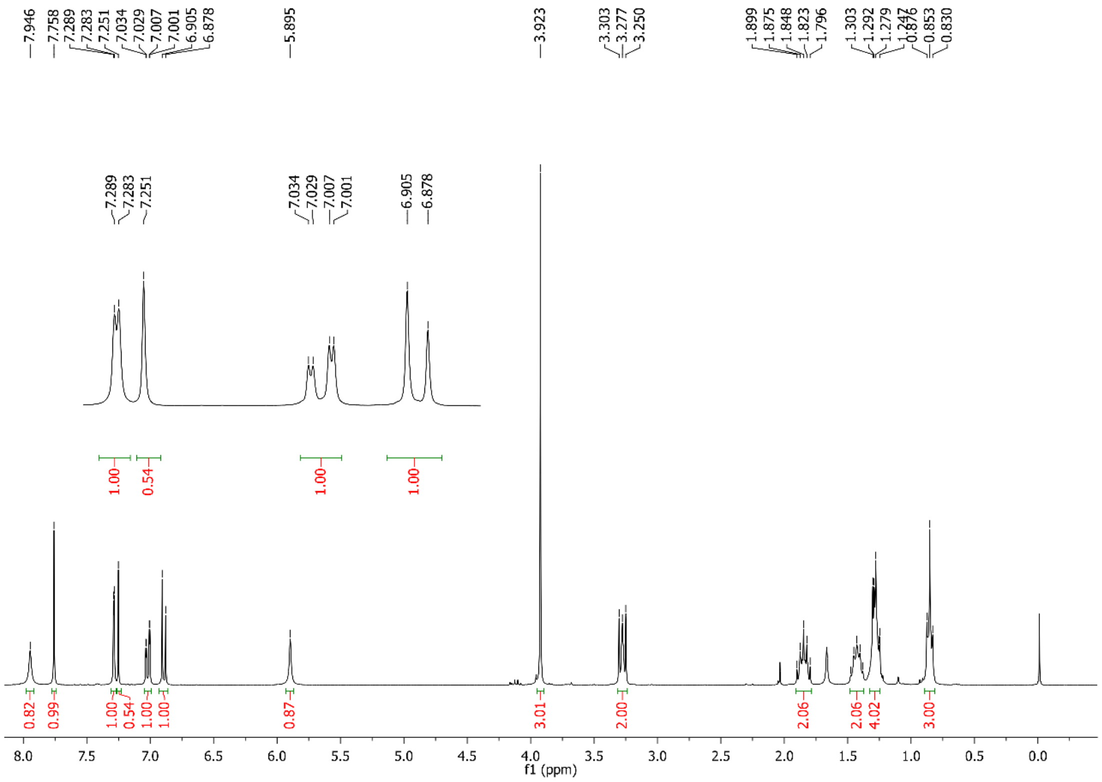

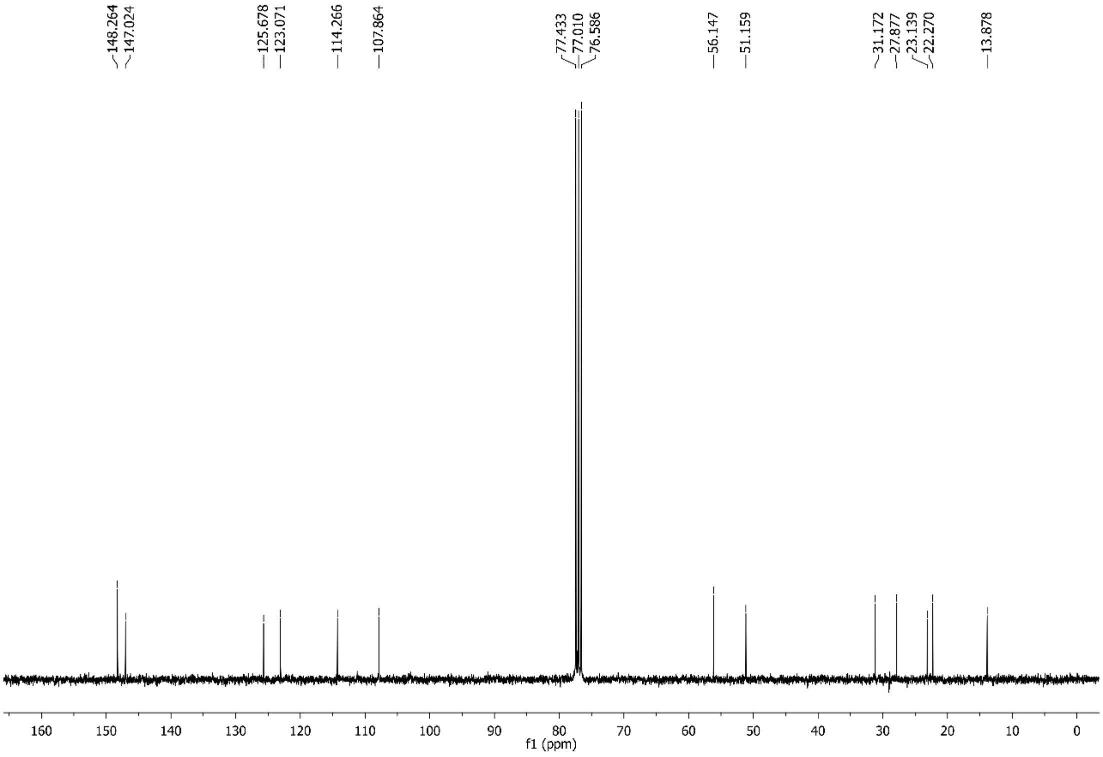

(E)-N'-(4-hydroxy-3-methoxybenzylidene)octane-1-sulfonohydrazide (**42**).

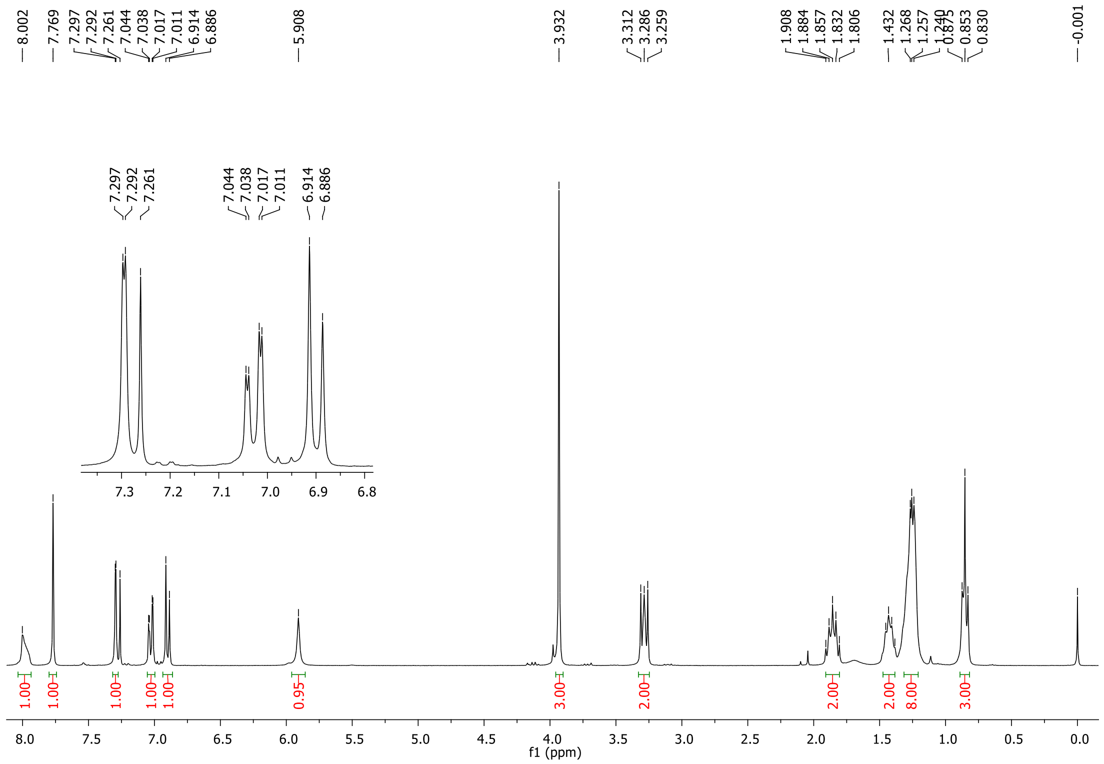

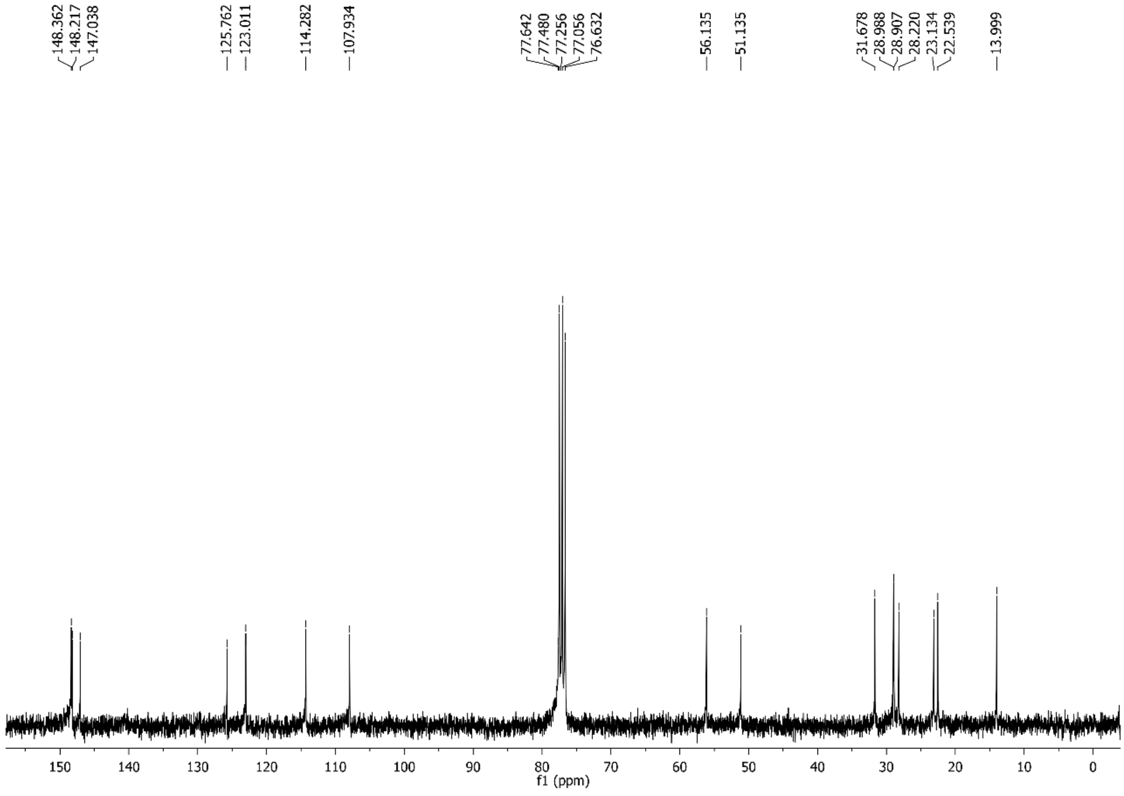

(E)-N'-(4-hydroxy-3-methoxybenzylidene)heptanehydrazide (**43**).

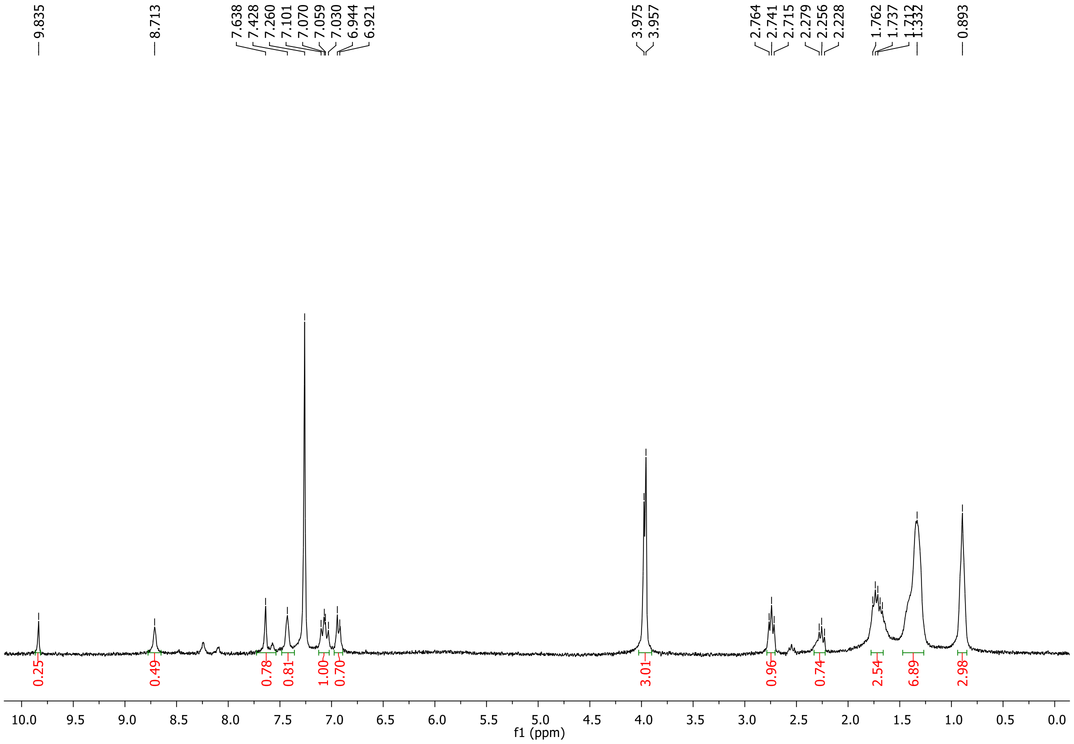

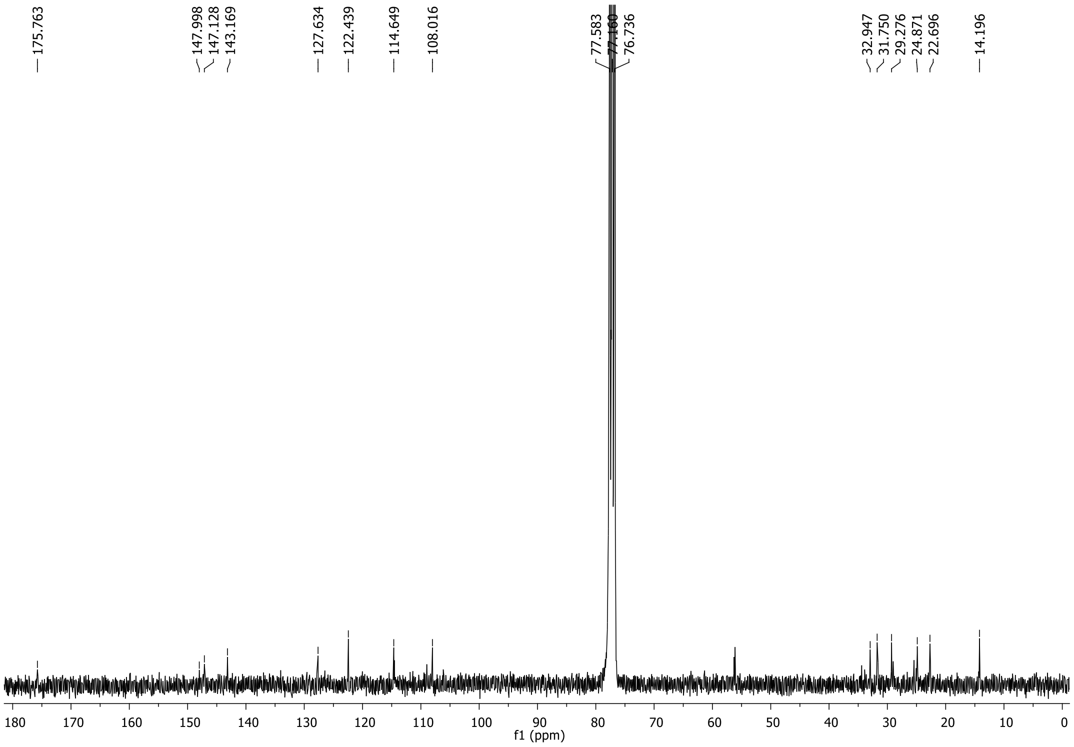

(E)-N'-(4-hydroxy-3-methoxybenzylidene)nonanehydrazide (**44**).

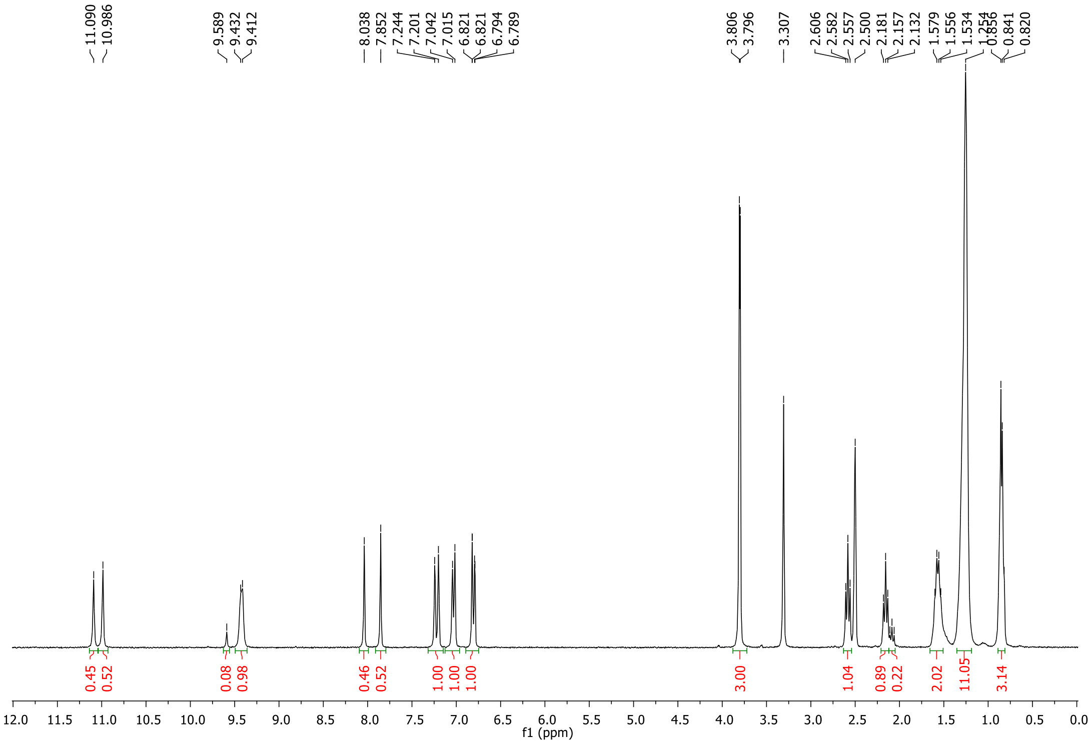

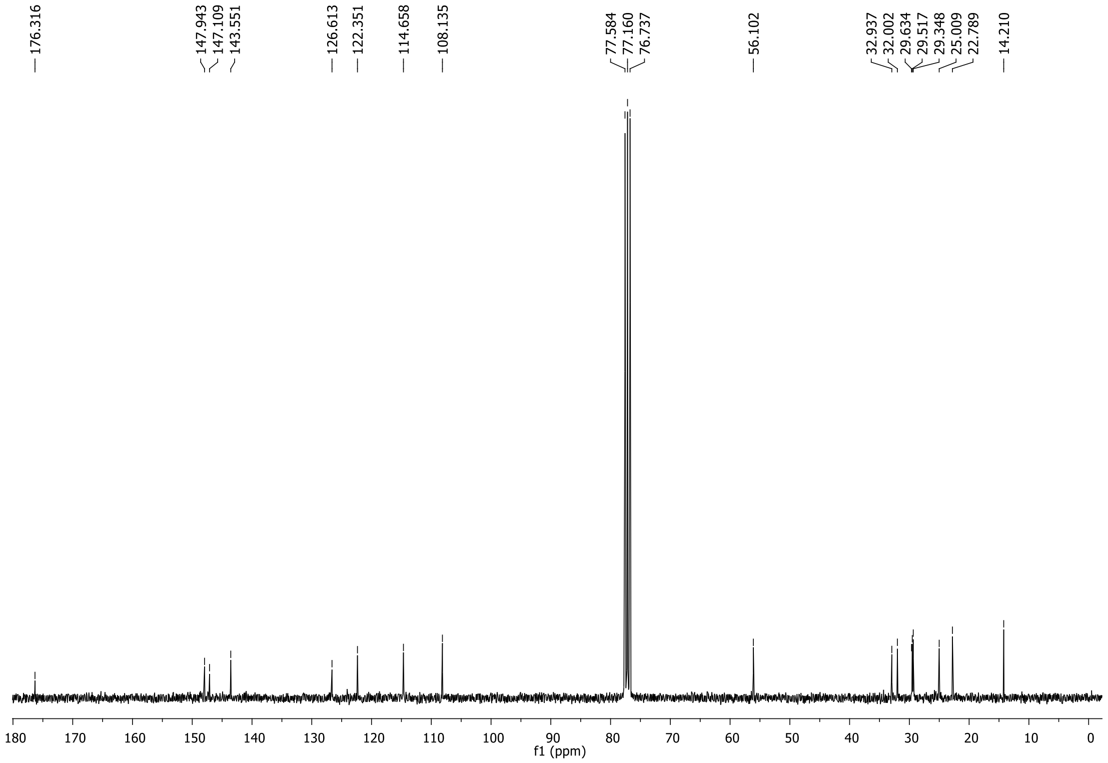

(E)-N-(2-(2-(4-hydroxy-3-methoxybenzylidene)hydrazineyl)-2-oxoethyl)hexane-1-sulfonamide (**45**).

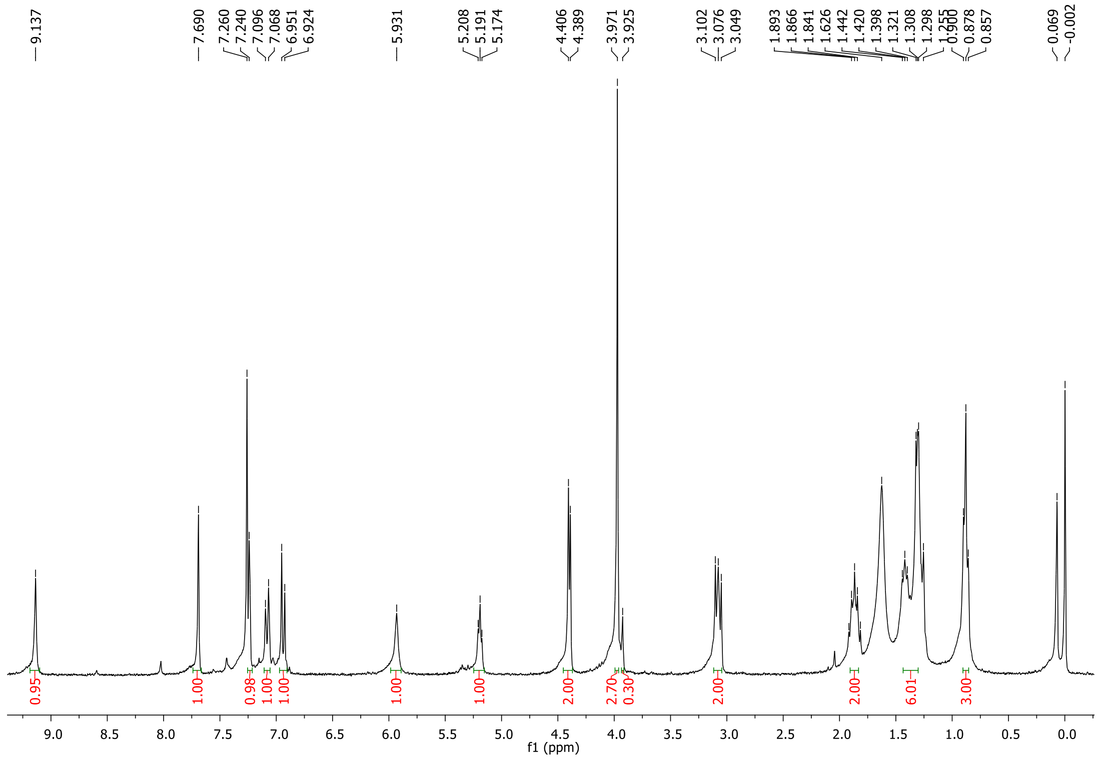

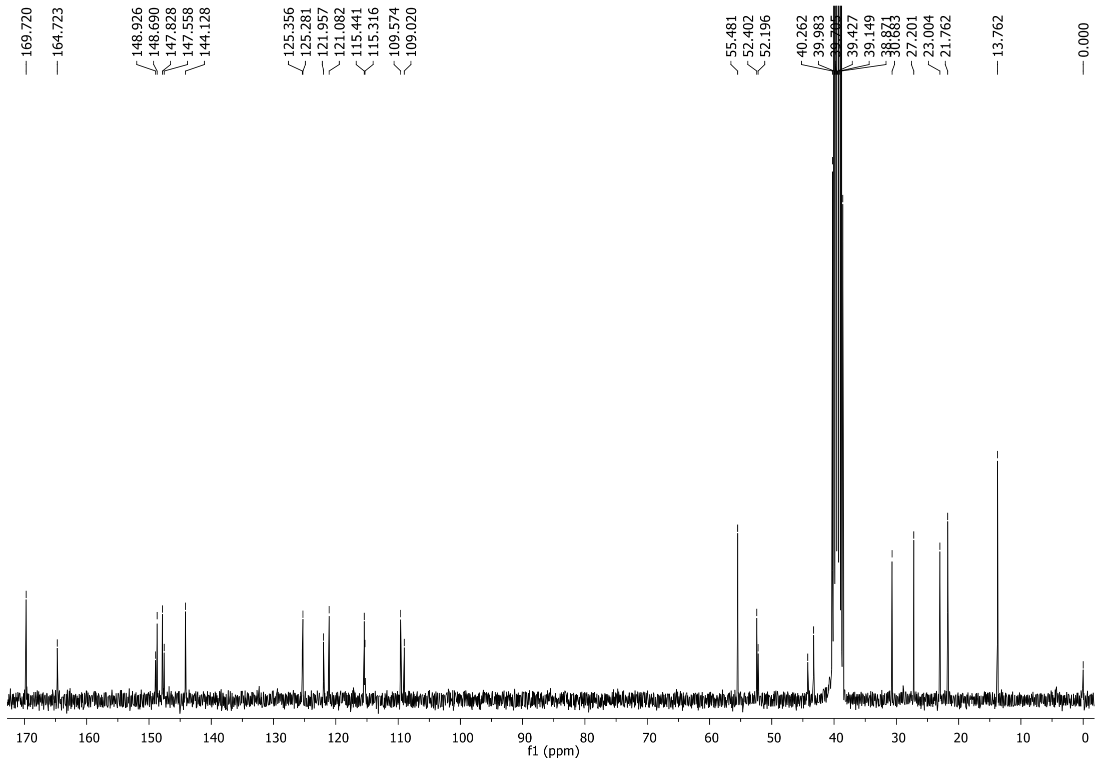

(E)-N-(2-(2-(4-hydroxy-3-methoxybenzylidene)hydrazineyl)-2-oxoethyl)octane-1-sulfonamide (**46**).

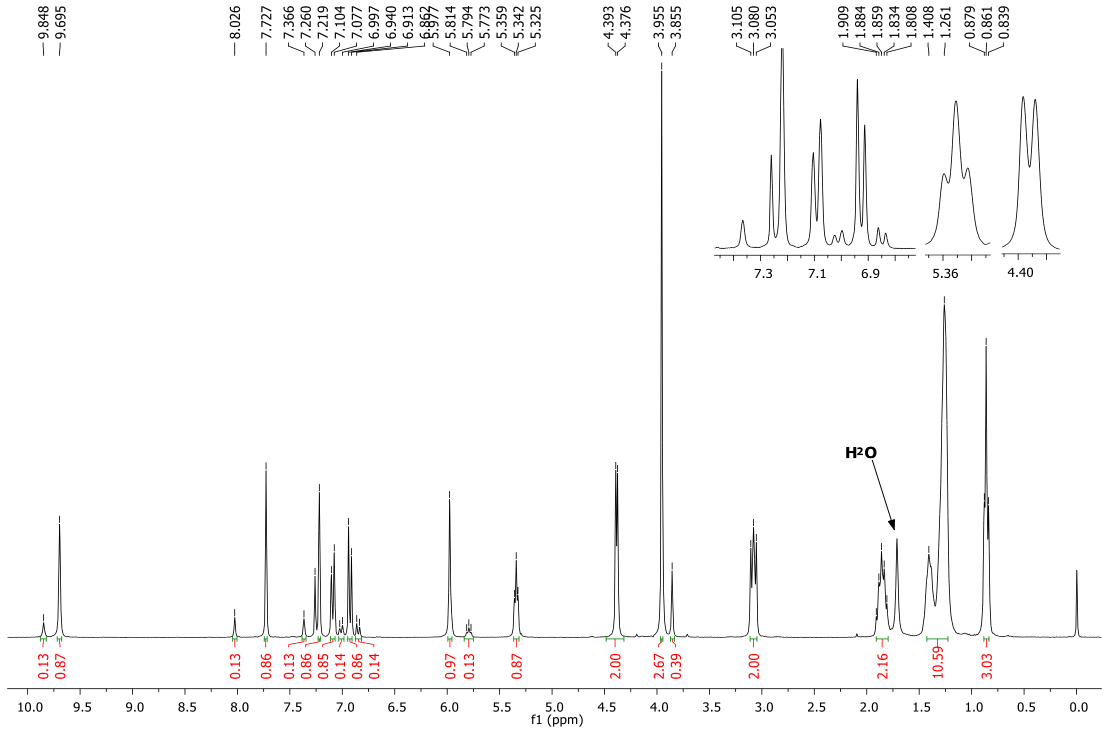

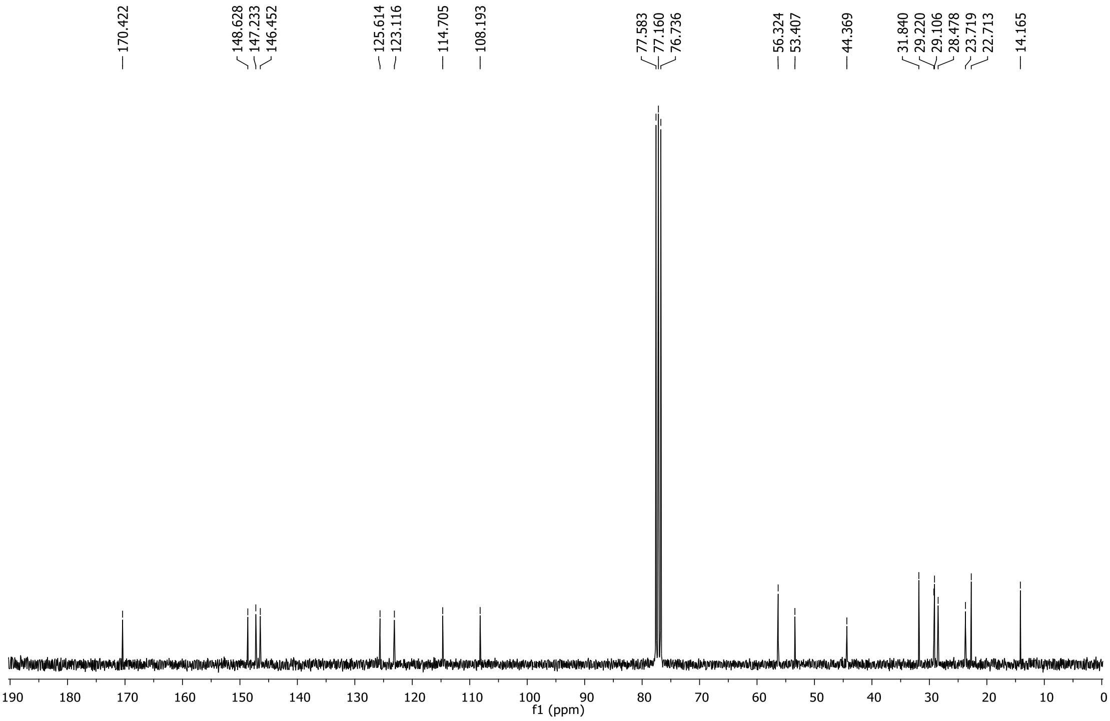

3. X-Ray crystal deposition

CCDC 1997204, 1997203, 1997202, 1997201, and CCDC 1997205 contain the supplementary crystallographic data for this paper. These data can be obtained free of charge from The Cambridge Crystallographic Data Centre via www.ccdc.cam.ac.uk/structures.

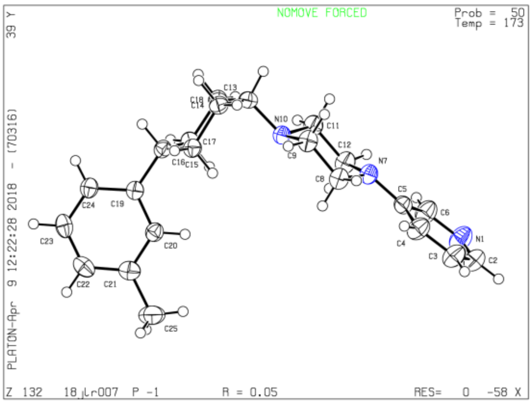

1

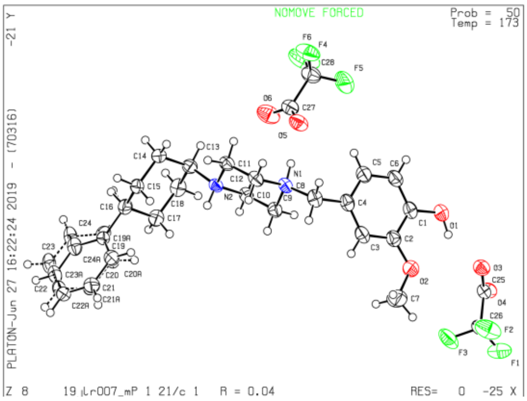

9

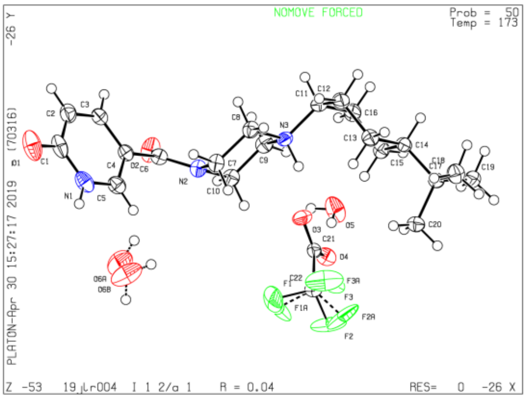

19

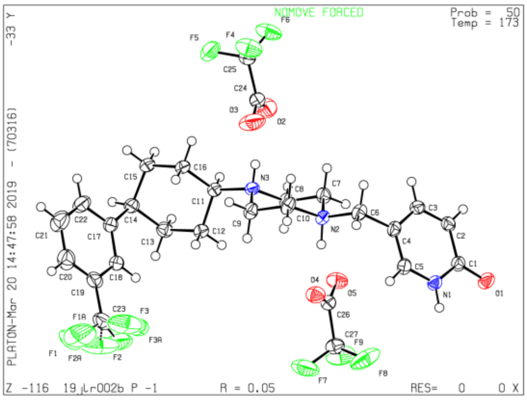

31

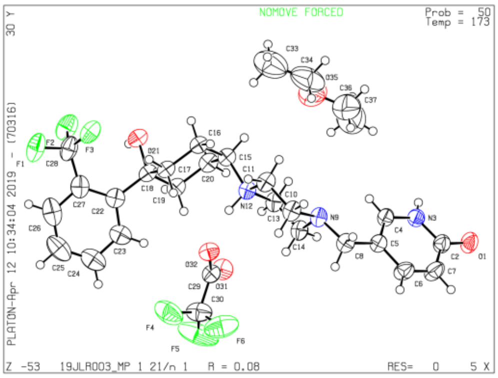

40

Fig S1. X-ray crystal structures of **1** (CCDC 1997204)**,** **9** (CCDC 1997203) **19** (CCDC 1997202)**,** **31** (CCDC 1997201)**,** and **40** (CCDC 1997205) shown as ORTEP with ellipsoids drawn at the 50% probability level. Hydrogen atoms (white spheres, arbitrary radius) were located in the difference Fourier map and refined freely. CCDC codes contains the supplementary crystallographic data for this paper.

4. Analytical purity for tested compounds

Table S1. Analytical RP-UHPLC purity compounds **6-40**

Compound	UPLC Purity (%)[a]	Compound	UPLC Purity (%)[a]
**6**	99%	**19**	99%
**7**	96%	**20**	> 99%
**8**	> 99%	**24**	> 99%
**9**	99%	**25**	98%
**11**	> 99%	**26**	96%
**12**	> 99%	**30**	> 99%
**13**	> 99%	**31**	99%
**14**	99%	**32**	99%
**16**	> 99%	**39**	> 99%
**17**	> 99%	**40**	> 99%

[a]UHPLC. Dionex ULTIMATE 3000 RSLC chromatography system; Column, Dionex Acclaim® RSLC 120 C18, 3.0 x 50 mm, particle size 2.2 μm, 120 Å pore size; UV Detection, ULTIMATE 3000 RS Photo diode array detector. Eluents, A: water with 0.05% TFA and D: Acetonitrile/water (9/1) with 0.05% TFA.

Table S2. Analytical RP-HPLC purity of compounds **41-46**

Compound	HPLC Purity (%)[a]
**41**	98%
**42**	96%
**43**	95%
**44**	97%
**45**	98%
**46**	97%

[a]HPLC. Shimadzu®-PROMINENCE system; Column, Waters®-μBondpak C18, 3.9 x 300 mm. UV Detection, Shimadzu® SPD-M10A VP Photo diode array detector. Eluents, A: water with 0.05% TFA and B: Acetonitrile with 0.05% TFA.

5. TRPV6 FLIPR assay

Calcium-5 was bought from Molecular Devices LLC. All other chemicals were purchased from Sigma-Aldrich.

*h*TRPV6 activity was measured using the HEK293 cell line stably overexpressing human TRPV6 as previously reported.[1,4] Stable cells were trypsinized and plated at 7.5 x 10^4 cells/well onto poly-D-lysine coated 96-well black plates with clear bottom using 100 µl DMEM supplemented with 10% FBS and 2 mM glutamine without antibiotics or phenol-red. After 16 h the medium was replaced with 90 µL of nominally calcium-free (NCF) loading buffer (modified Krebs buffer containing 117 mM NaCl, 4.8 mM KCl, 1 mM MgCl 2, 5mM D-glucose, 10 mM HEPES, and calcium-5 fluorescence dye (50 µL/mL loading buffer)). Cells were incubated in the NCF-loading buffer at 37 °C for 1 h. Fluorescence Cd^{2+} measurements were carried out using FLIPR^{TETRA} high throughput (Molecular Devices, LLC), fluorescence microplate reader. Cells were excited using a 470-495 nm LED module, and the emitted fluorescence signal was filtered with a 515-575 nm emission filter (manufacturer's guidelines). Stable Ca^{2+}-free baselines were established for 50 seconds before 10 µL of a 10X compound was added to the cells. Cells were incubated at 37 °C and fluorescence was monitored in the presence of compound for an additional 5 minutes before administration of 100 µL of $CdCl_2$ (final concentration: 50 µM). The activity of TPRV6 was measured by calculating the area under the curve of the Cd^{2+} entry traces.

Screening experiments were done with 3 to 6 repeats per group at 10 µM (**6-9, 11-14, 16-17, 19-20, 24-26, 30-32,** and **39-40**) or 50 µM (**41-46**). Fluorescence signals were analyzed using the ScreenWorks 3.1.1.8 software (Molecular Devices). Dose-response curves were generated (9-point curve, 6 repeats/concentration, 2-fold serial dilution starting at 10 µM), and the IC_{50} values were extrapolated from these plots for each compound (GraphPad® Prism, v. 5.0, San Diego, CA, US). Inhibition curves were obtained by non-linear regression using the built-in log(inhibitor) vs. response-variable slope function (four parameters).

6. TRPV5 FLIPR assay

*h*TRPV5 activity was measured in a similar experiment to as described above.[5] Briefly, HEK293 cells were trypsinized and plated at 1.5 x10^4 cells/well onto Corning® 96-well black polystyrene clear bottom microplates (CLS3603 Sigma-Aldrich) coated with 100 µg/mL poly-D-lysine (P6407 Sigma-Aldrich) using 100 µL phenol-red free DMEM with 10% FBS and 2mM glutamine without antibiotics. Cells were incubated at 37 °C for 24 h. on the following day, transfection was performed using 200 ng of pTagRFP-C1-hTRPV5 and 0.6 µL Lipofectamine 2000 reagent/well. Fluorescent ion measurements using FLIPR^{TETRA} were carried out 24h post-transfection. The FLIPR protocol for measuring *h*TRPV5 activity was identical to the one described for *h*TRPV6.

7. TRPV1 FLIPR assay

*h*TRPV1 activity was measured using HEK293T cells transiently overexpressing TRPV1 as previously reported.[6] Briefly, HEK293 cell were trypsinized and plated at 1.5 x104 cells/well onto Corning® 96-well black polystyrene clear bottom microplates (CLS3603 Sigma-Aldrich) coated with 100 µg/mL poly-D-lysine (P6407 Sigma-Aldrich) using 100 µL phenol-red free DMEM with 10% FBS and 2mM glutamine without antibiotics. Cells were incubated at 37 °C for 24 h. On the following day, transfection was performed using 200 ng of pcDNA 3.1 hTRPV1 and 0.6 µL Lipofectamine 2000 reagent/well. 24 h after the transfection, the medium was replaced with 90 µL of loading buffer (modified Krebs buffer containing 117 mM NaCl, 4.8 mM KCl, 1 mM $MgCl_2$, 5 mM D-glucose, 10 mM HEPES, 1.8 mM $CaCl_2$ and calcium-5 fluorescence dye (50 µL/mL loading buffer)). Cells were incubated in the loading buffer at 37 °C for 1 h in dark. Fluorescence Ca^{2+} measurements were carried out using FLIPR^{TETRA} high-throughput, fluorescence microplate reader as described before. Stable baselines were established for 50 s before 10 µL of a 10X solution of compound **39** or the TRPV1-inhibitor capsazepine (CPZ) prepared in 1.8 mM $CaCl_2$-containing Krebs buffer was robotically administered to the cells. Cells were incubated and fluorescence was monitored in the presence of compound for an additional 5 min before administration of the agonist in 1.8 mM $CaCl_2$-containing Krebs buffer (final concentration of capsaicin in the assay plate was 100 nM). The activity of TRPV1 was measured by quantifying the area under the curve (AUC) of the fluorescence intensity, following administration of the agonist.

The current HEK-*h*TRPV1 method employs a constant concentration of Ca^{2+} (1.8 mM) in the assay buffer during the whole experiment.[6] We observed that addition of compound **39** at 10.0 µM did not activated the channel, as the recorded baseline remained comparable to DMSO treatment. Upon addition of capsaicin (100 nM) the channel was activated and a high influx of Ca^{2+} was immediately recorded. The activation of the TRPV1 channel is supposed to occur through displacement of lipids in the transmembrane domain. This allosteric effect was investigated in depth through functional assays, by co-crystallization/cryo-EM methods, molecular dynamics simulations and mutagenesis.[7–9] The O-methylcathecol head and the amide linker of capsaicin are the pharmacophoric features responsible for its binding to TRPV1. The long hydrophobic tail mediates Van der Waals interactions with apolar residues, resembling the interactions found for the phospholipids.[10] We believe that the replacement of the O-methylcathecol to pyridine/pyridone and the amide to piperazine in compounds **1** and **39** explains their lack of activity on TRPV1.

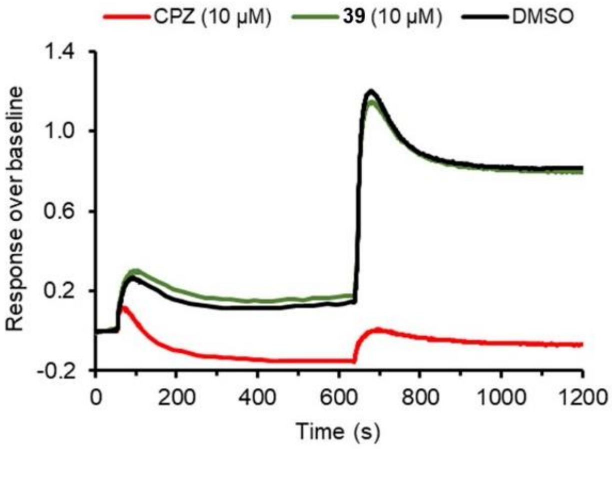

Fig S2. Average recording of Ca^{2+} entry in HEK-*h*TRPV1 cells pretreated for 5 min with either DMSO, capsazepine (CPZ) or **39**. Data shown is mean (n = 3) of a single experiment. Ca^{2+} influx was achieved by 10 min treatment of cells with 100 nM capsaicin in 1.8 mM $CaCl_2$ containing Krebs buffer. Quantification reveals that 10 µM of **39** inhibited 2% the Ca^{2+} influx through capsaicin-activated *h*TRPV1. The positive control CPZ inhibited 100% at 10 µM.

8. Electrophysiology

HEK293 cells (DSMZ, Germany) were cultivated in DMEM medium containing 10% FCS, 100 µg/ml streptomycin and 100 U/ml penicillin. 24 h before the experiments, a transient transfection with the peYFP-C1 TRPV6 plasmid was performed by utilising TransFectinTM Lipid Reagent (Bio-Rad). Electrophysiological experiments were conducted at room temperature (20 - 24°C) in the whole-cell configuration with an Ag/AgCl reference electrode. Voltage ramps over a time period of 200 ms from -90 to +90 mV were performed every 5 s with a holding potential of 50 mV. The internal pipette solution consisted of 145 mM Cs-methanesulfonate, 8 mM NaCl, 5 mM $MgCl_2$, 10 mM HEPES and 20 mM EGTA, pH 7.2. The extra cellular solution (10 mM Ca^{2+}) contained: 145 mM NaCl, 5 mM CsCl, 1 mM $MgCl_2$, 10 mM HEPES, 10 mM glucose and 10 mM $CaCl_2$, pH 7.4. The leak-correction of the currents were conducted by subtraction of the remaining leak currents after La^{3+} bock. The liquid junction potential was determined as 12 mV, though applied voltages were not corrected. All patch-clamp experiments were conducted at least on 2 different days. As a control the application of the equivalent amount (as of the inhibitor) of DMSO in 10 mM Ca^{2+} solution was used. Graphs and statistical analyses were performed with OriginPro software (version 9.1, for Windows, OriginLab, Northampton, MA). The unpaired t-test was conducted to statistically evaluate the effect of the inhibitors.

9. SOCE FLIPR assay

MDA-MB-231 cells were trypsinized and plated at 6 x 10^4 cells/well onto 96-well black plates with clear bottom using 100 µl phenol-red free RPMI medium supplemented with 10% FBS. SOCE activity was measured 16 h later using the previously described FLIPR assay.[11] After 16 h the medium was replaced with 50 µL of NCF loading buffer and the cells were incubated at 37 °C for 40 min, following which 50 µL of 2X drugs were manually applied (GSK-7975A, **1** or **39**) and the cells were incubated for another 20 min. The SOCE inhibitor GSK-7975A (cat. no. AOB4124-1) was purchased from Aobious, Gloucester, MA, USA. Stable Ca^{2+}-free baselines were established for 50 seconds before 50 µL of a 3X thapsigargin (Tg) was robotically administered to the cells. Cells were incubated at 37 °C and fluorescence was monitored in the presence of Tg for an additional 10 minutes before administration of 50 µL of 4X $CaCl_2$. The activity of SOCE was measured by calculating the area under the curve of the Ca^{2+} entry traces.

10. Confocal microscopy

HEK-hTRPV6 were trypsinized and plated at 1 x 104 cells/well onto poly-D-lysine coated Nunc Lab-Tek II 8-well chambered coverglass plates (Faust Laborbedarf AG, Schaffhausen) using 100 µl DMEM supplemented with 10% FBS and 2 mM glutamine without antibiotics or phenol-red. After 24 h of incubation at 37 °C, the medium was replaced by 200 µL/well of a mixture of Leadmiun Green (final concentration: 5 ng/µL), Hoechst 33258 (final concentration: 1 ng/µL) and wheat germ agglutinin Alexa Fluor® 594 conjugate (final concentration: 5 ng/µL) in NCF buffer. The chamber was covered with aluminum foil and was incubated at 37 °C for 30 min. Then, the buffer was removed, cells were washed twice with fresh NCF buffer (2 x 200 µL) and another 190 µL of NCF containing 10X of 39 was added. For control cells, 200 µL of NCF was added after washing. The chamber was mounted in the confocal microscope and 10 µL of CdCl2 (final concentration: 50 µM) was added right after the start of imaging (total duration: 30 min).

The cells were imaged at 100X lenses with a confocal, laser scanning microscope setup using a Nikon Eclipse TE2000-E fully automatized inverted, epifluorescence microscope outfitted with Nikon D-Eclipse C1 laser confocal optics. The system equipped with a violet-diode (405 nm) and a multiline Argon (457-515 nm) from Melles Griot, and a Helium/Neon (594 nm) lasers from JDS Uniphase. Nikon EZ-C1 3.6 confocal imaging software installed on an HP xw4400 workstation was used for image acquisition. Brightness and contrast were adjusted with ImageJ.

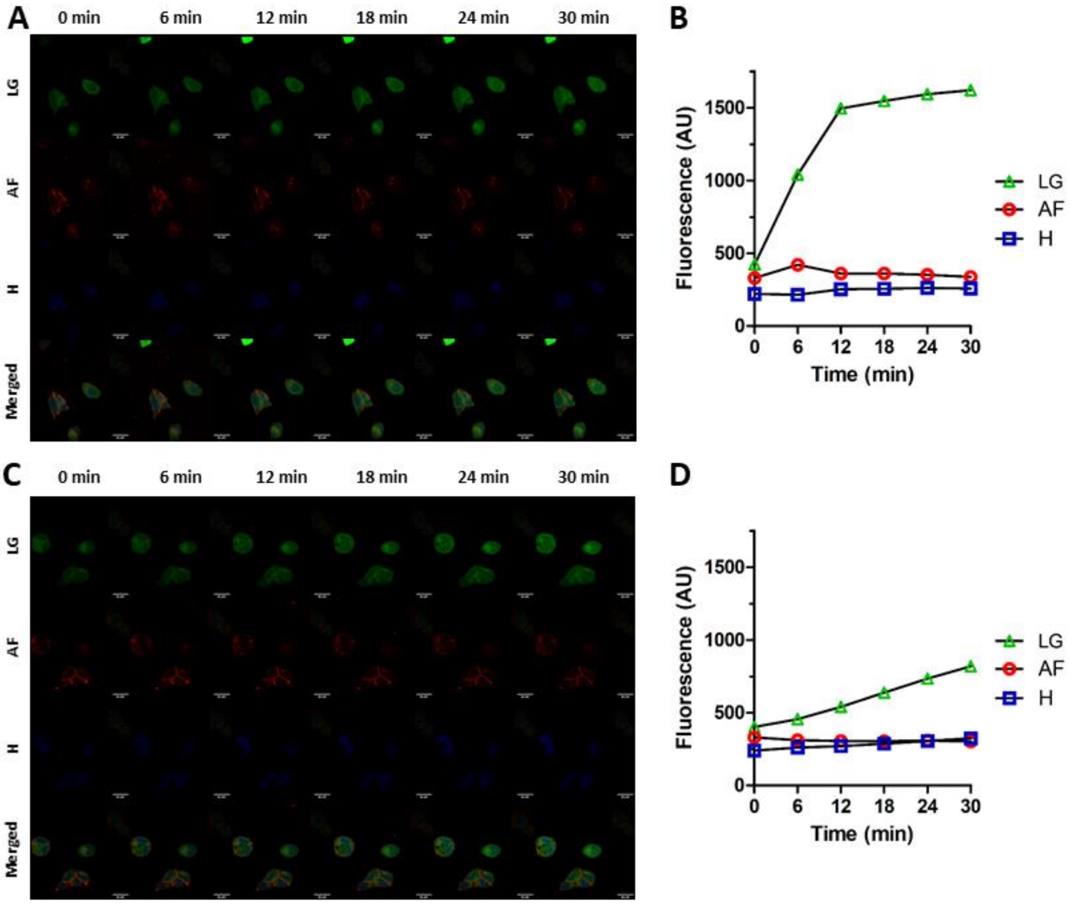

Fig S3. Time-course of Cd^{2+} uptake in HEK-hTRPV6 cells with Leadmiun Green (LG), wheat germ agglutinin Alexa Fluor® 594 conjugate (AF), and Hoechst 33258 (H) along 30 min. Images were collected using confocal microscopy (Nikon Eclipse TE2000-E, 100X). HEK-hTRPV6 cells were incubated with fluorescent dyes for 30 min at 37 °C. To these cells DMSO (A) or **39** (10 µM, C) was applied followed by a solution of Cd^{2+} (50 µM). The total fluorescence intensity for each channel was plotted in graphs (B) and (D), respectively for DMSO and compound **39**. Images were collected with excitation for LG at λ_{ex} = 488 nm and emission at λ_{em} = 520 nm, H at λ_{ex} = 352 nm and emission at λ_{em} = 461 nm, and AF at λ_{ex} = 590 nm and emission at λ_{em} = 617 nm. White bars denote 20 µm.

11. Cadmium toxicity

The XTT assay was used to evaluate cell toxicity. HEK293 wt and HEK-hTRPV6 cells were plated at a density of 1.5 x 104 cells/well in 96-well plates in DMEM medium supplemented with 10% FBS, 2mM L-Glutamine, 100 U/ml Penicillin, 100 µg/ml Streptomycin (Sigma) and 1% non-essential amino acids (Bioconcept, Switzerland) and were incubated at 37 °C for 24 h. On the following day, the medium was replaced with DMEM medium containing CdCl2 at concentrations ranging from 50 to 0.05 µM. 39 was used in the treatment group at either 1.0 and 10 µM. The blank consisted in medium containing only DMSO (0.01%). Cells were incubated for 24 h at 37 °C. Then, 25 µL of XTT (with 1.25 % of PMS) was added to each well and incubated at 37 °C for 2 h. Subsequently, the absorbance was read at 650 nm and subtracted from the absorbance of 450 nm by spectrophotometry (Vmax Kinetic Microplate Reader, Molecular Devices LLC). The resulting subtracted absorbance for each Cd2+ concentration (At) was expressed as a percentage of viable cells relative to the blank (Ab)

$$\%\ viable\ cells = \frac{A_t}{A_b} x100$$

Where A_t: treatment absorbance; A_b: blank absorbance.

The percentage of viable cells was plotted, and the inhibition curves obtained by non-linear regression using the built-in log(inhibitor) vs. response-variable slope function (GraphPad® Prism, v. 5.0, San Diego, CA, US).

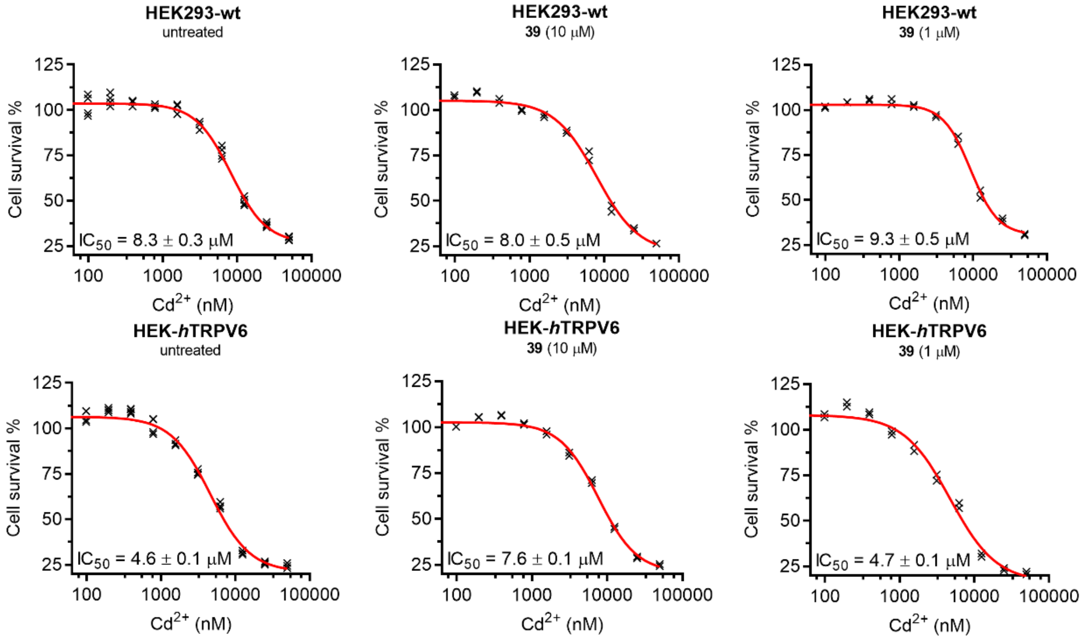

Fig S4. XTT cell viability curve of HEK293 wt and HEK-hTRPV6 under Cd^{2+} in presence or not of **39** (1.0 and 10 µM). Data shown represent each replicate (n = 4/concentration) from 2 independent experiments.

12. Antiproliferative activity

Unless specified cell culture reagents were obtained from Gibco, Life Technology, Switzerland. MCF-7 (human mammary adenocarcinoma), MDA-MB-231 (human mammary adenocarcinoma) were obtained from the ATCC cell bank. T47D (human mammary ductal carcinoma) cell line was obtained from NIH cell collection. These cells were grown in RPMI medium complemented with 10% FBS, 2mM L-Glutamine, 100 U/ml Penicillin and 100 µg/ml Streptomycin (Sigma) and 1% non-essential amino acids (Bioconcept, Switzerland). HEK293 (human embryonic kidney) and SKOV-3 (human ovary adenocarcinoma) cell lines were obtained from the ATCC cell bank. HEK-hTRPV6 and HEK293 and SKOV-3 were grown in DMEM medium supplemented with 10% FBS, 2mM L-Glutamine, 100 U/ml Penicillin and 100 µg/ml Streptomycin (Sigma) and 1% non-essential amino acids (Bioconcept, Switzerland).

The XTT assay was used to evaluate cell proliferation. Cells were plated at a density of 5 x 103 cells/well in 96-well plates and were incubated at 37 °C for 24 h. On the following day, the medium was carefully aspirated and replaced with 100 µL of a 39 or 1 at concentrations ranging from 100 µM to 0.4 µM. Doxorubicin (10 µM) and DMSO (0.01 %) were used as positive and negative controls, respectively. All the treatments used the original cell medium (RPMI or DMEM), depending on the cell line. Cells were incubated for a total of 6 days at 37 °C with the medium (treatment and controls) being replaced by a freshly prepared solution every 48 h. Then, 25 µL of XTT (with 1.25 % of PMS) was added to each well and incubated at 37 °C for 2 h. Subsequently, the absorbance was read at 650 nm and subtracted from the absorbance of 450 nm by spectrophotometry (Vmax Kinetic Microplate Reader, Molecular Devices LLC). The resulting subtracted absorbance for each compound concentration (At) was expressed as a percentage of viable cells relative to the negative control (Ab), and the percentage of viable cells was plotted and the inhibition curves obtained by non-linear regression using the built-in log(inhibitor) vs. response-variable slope function (GraphPad® Prism, v. 5.0, San Diego, CA, US).

Table S3. IC_{50} activity of **1** and **39** against several breast cancer and HEK293 cell lines

**Cell line**	**IC_{50} (µM)[a]**	
	**1**	**39**
T47D	71.4 ± 1.0	ND
MCF7	74.1 ± 1.0	ND
MDA-MB-231	> 100	ND
SKOV3	36.4 ± 1.5	ND
HEK293 wt	> 100	ND
HEK-*h*TRPV6	29.1 ± 0.6	ND

[a]IC_{50} values of **1** and **39** against a panel of cancer cell lines. Data shown are mean ± SEM (n = 3/concentration) of at least 3 independent experiments. ND = not determined, no inhibition of cell growth.

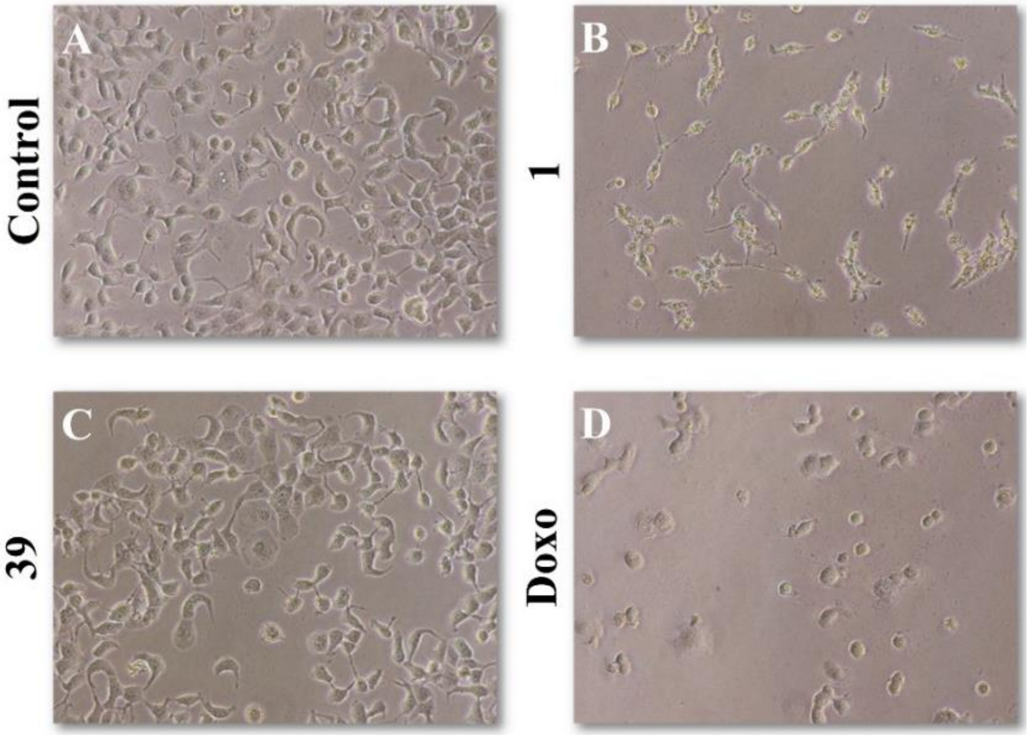

Fig S5. Photomicrographs show the morphological aspects of the control cultures of T47D cells (A), and treated with **1** (100 µM, B); **39** (100 µM, C); doxorubicin (10 µM, D). Cells were imaged at 20X with an inverted microscope (Nikon Eclipse TiU).

13. References

1 M. R. Cunha, R. Bhardwaj, S. Lindinger, C. Butorac, C. Romanin, M. A. Hediger and J.-L. Reymond, Photoswitchable Inhibitor of the Calcium Channel TRPV6, *ACS Med. Chem. Lett.*, 2019, **10**, 1341–1345.

2 T. F. Silva, W. Bispo Júnior, M. S. Alexandre-Moreira, F. N. Costa, C. Monteiro, F. Furlan Ferreira, R. C. R. Barroso, F. Noël, R. T. Sudo, G. Zapata-Sudo, L. M. Lima and E. Barreiro, Novel Orally Active Analgesic and Anti-Inflammatory Cyclohexyl-N-Acylhydrazone Derivatives, *Molecules*, 2015, **20**, 3067–3088.

3 A. Lopes, E. Miguez, A. Kümmerle, V. Rumjanek, C. Fraga and E. Barreiro, Characterization of Amide Bond Conformers for a Novel Heterocyclic Template of N-acylhydrazone Derivatives, *Molecules*, 2013, **18**, 11683–11704.

4 C. Simonin, M. Awale, M. Brand, R. Van Deursen, J. Schwartz, M. Fine, G. Kovacs, P. Häfliger, G. Gyimesi, A. Sithampari, R. P. Charles, M. A. Hediger and J. L. Reymond, Optimization of TRPV6 calcium channel inhibitors using a 3D ligand-based virtual screening method, *Angew. Chem. - Int. Ed.*, 2015, **54**, 14748–14752.

5 G. Kovacs, N. Montalbetti, M.-C. Franz, S. Graeter, A. Simonin and M. A. Hediger, Human TRPV5 and TRPV6: Key players in cadmium and zinc toxicity, *Cell Calcium*, 2013, **54**, 276–286.

6 G. J. V. Pereira, M. T. Tavares, R. A. Azevedo, B. B. Martins, M. R. Cunha, R. Bhardwaj, Y. Cury, V. O. Zambelli, E. G. Barbosa, M. A. Hediger and R. Parise-Filho, Capsaicin-like analogue induced selective apoptosis in A2058 melanoma cells: Design, synthesis and molecular modeling, *Bioorg. Med. Chem.*, 2019, **27**, 2893–2904.

7 L. Darré and C. Domene, Binding of Capsaicin to the TRPV1 Ion Channel., *Mol. Pharm.*, 2015, **12**, 4454–2265.

8 E. Cao, M. Liao, Y. Cheng and D. Julius, TRPV1 structures in distinct conformations reveal activation mechanisms, *Nature*, 2013, **504**, 113–118.

9 F. Yang and J. Zheng, Understand spiciness: mechanism of TRPV1 channel activation by capsaicin, *Protein Cell*, 2017, **8**, 169–177.

10 Y. Gao, E. Cao, D. Julius and Y. Cheng, TRPV1 structures in nanodiscs reveal mechanisms of ligand and lipid action., *Nature*, 2016, **534**, 347–351.

11 R. Bhardwaj, B. Augustynek, E. Ercan-Herbst, P. Kandasamy, M. Seedorf, C. Peinelt and M. A. Hediger, Ca2+/Calmodulin Binding to STIM1 Hydrophobic Residues Facilitates Slow Ca2+-Dependent Inactivation of the Orai1 Channel, *Cell. Physiol. Biochem.*, 2020, **54**, 252–270.
